# Giant Fully Fused Tetrapodal Rylenimides: Design, Synthesis and Optoelectrochemical Characterization via Alkyl Chain and Core Engineering Strategies

**DOI:** 10.1021/acs.orglett.5c02655

**Published:** 2025-08-25

**Authors:** Matías J. Alonso-Navarro, Fátima Suárez-Blas, José Ignacio Martínez, M. Mar Ramos, José L. Segura

**Affiliations:** † Department of Organic Chemistry, Complutense University of Madrid, Faculty of Chemistry, Madrid 28040, Spain; ‡ Chemical and Environmental Technology Department, Univ. Rey Juan Carlos, Móstoles 28933, Spain; § Department of Low-Dimensional Materials, Institute of Materials Science of Madrid (ICMM-CSIC), Madrid 28049, Spain

## Abstract

In this work, we report the synthesis and comprehensive characterization of a new series of fully fused three-dimensional rylenimide-based derivatives featuring extended π-conjugation through core-fusion strategies and tailored side-chain engineering at the imide nitrogen, resulting in molecular architectures with up to 19 fused rings. The effects of π-extension and alkyl/aryl imide substituents on the optical and electrochemical behavior were systematically studied using UV–vis spectroscopy, cyclic voltammetry, and density functional theory calculations. The results demonstrate that combining π-extension with bulky solubilizing groups effectively suppresses undesired π–π interactions, enhances electronic delocalization, and improves device-relevant properties. This molecular design strategy offers a promising platform for the development of next-generation functional *n-*type organic semiconductors.

## Graphical abstract

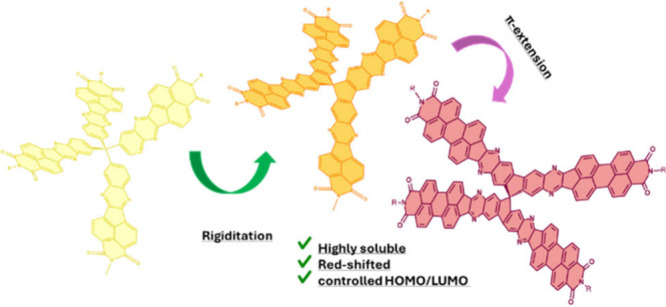

The development of *n*-type organic semiconductors has attracted growing interest due to their potential in devices such as OFETs, OPVs, and OLEDs.^1^ Unlike *p*-type materials, *n*-type systems have historically faced limitations in stability, electron mobility, and processability. However, recent advances in electron-deficient molecular and polymeric systems have led to significant improvements in device performance.^2^ Among current strategies, the design of three-dimensional π-conjugated architectures has emerged as a promising approach to enhance charge transport, solubility, and thermal stability.^3^ Nonetheless, their synthesis remains challenging, and achieving uniform film formation can be difficult. Extended π-conjugation in *n-*type materials is of particular interest, as it can significantly enhance electronic properties by promoting delocalized charge transport,^4^ thereby leading to higher electron mobility and improved overall device performance.^5^ Nevertheless, excessive π–π stacking can promote aggregation and phase separation, negatively impacting film uniformity. Therefore, balanced molecular design is crucial to achieve high-performance materials with good processability. In parallel, side-chain engineering has proven to be a powerful approach to fine-tuning material properties. The introduction of flexible side chains not only improves solubility and enables compatibility with solution-based deposition techniques such as spin-coating or inkjet printing,^6^ but also allows modulation of the (opto)­electronic behavior through noncovalent interactions within the molecular framework.^7^ Within this framework, rylene mono- and diimides have emerged as particularly attractive building blocks due to their excellent electron-accepting properties, high thermal and chemical stability, and tunable photophysical characteristics, making them well-suited for a wide range of advanced applications in organic electronics.^8^ In recent work, Segura’s group has successfully developed a series of functional 3D materials based on these cores, demonstrating their applicability in areas such as organic photovoltaics,^9^ environmental remediation and photoanodes for water-splitting.^10^ These studies underscore the importance of tuning dimensionality to achieve a deeper understanding of structure-performance relationships.

Building on these foundations, we report here the design and synthesis of a new series of naphthalimide and perylenimide derivatives. We aim to systematically evaluate: (i) the impact of core-fusion strategies compared to previously reported unfused semiconductors, (ii) the enhanced photophysical behavior of three-dimensional assemblies relative to their one-dimensional molecular analogues, (iii) the influence of various alkyl chain substitutions at the imide nitrogen atom, and (iv) the role of π-extension in the fully-fused core on their optical and electrochemical properties. The synthesis of these novel 3D rylenimide-based semiconductors (Scheme 1) begins with the preparation of the corresponding naphthalimide and perylenimide 1,2-dione derivatives (**NID** and **PID** respectively), both previously reported by the group.^11^ These electroactive moieties have been selectively functionalized with different alkyl/aryl chain at the nitrogen atom of the imide group to achieve two main objectives: (i) to obtain processable materials and (ii) to assess the impact of these solubilizing groups on the optical and electrochemical properties of the resulting assemblies. In parallel, the central 9,9′-spirobi­[fluoren]-2,2′,3,3′,6,6′,7,7′-octaamine hydrochloride **1** was synthesized following the method previously reported by Pyka et al.^12^ The condensation reaction between the electron-deficient imides and the spiro-compound yields the corresponding pyrazine-based fully fused tetrapodal rylenimides **4NIPBSP** and **4PIPBSP** in good yields. This synthetic approach not only yields rigid structures but also facilitates an efficient extension of conjugation in the rylenimide moieties, resulting in N-doped polycyclic aromatic dicarboximide (PADI) systems with 13 or 19 fused rings. In addition to these three-dimensional π-extended semiconductors, we also synthesized the corresponding 1D analogues, **NIPB** and **PIPB**, to evaluate the effect of π-extension on the opto-electrochemical properties of these new assemblies.

**Scheme 1:**
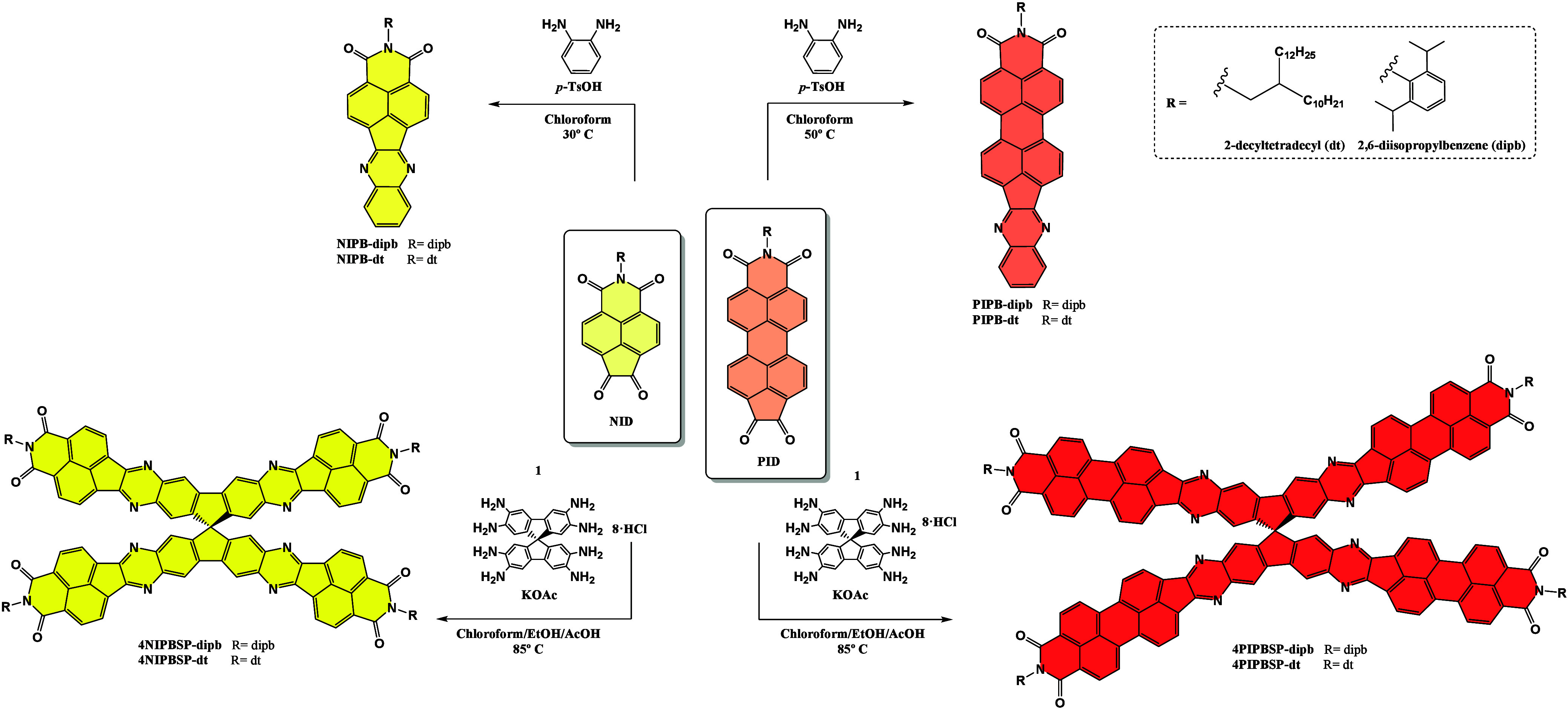
Synthetic Routes Developed for the Obtention of These Tridimensional Rylenimide Semiconductors

The design and synthesis of organic semiconductors which possess planar, π-extended and conjugated skeletons often face challenges due to aggregation phenomena or low solubility, which typically hinders processability and limit their application in organic electronics.^13^ However, the strategic incorporation of long, branched alkyl chains, combined with attenuated π interactions in three-dimensional core-engineered derivatives, enables the production of fully fused and π-conjugated materials that are both processable and exhibit high solubility.^14^ These materials demonstrate solubilities exceeding 20 mg/mL in typical organic solvents such as dichloromethane, chloroform, toluene or THF at room temperature. Consequently, the rylenimide-based molecular assemblies have undergone comprehensive characterization via nuclear magnetic resonance (^1^H and ^13^C NMR), Fourier-transform infrared spectroscopy (FT-IR) and high-resolution mass spectrometry (HRMS) techniques. Notably, for the **PIPB-dipb** derivative, the incorporation of bulky 2,5-diisopropylaniline at the imide position was insufficient to enhance the solubility of this nine-ring fused assembly, and only the addition of small amounts of deuterated trifluoroacetic acid (99%, 0.1 mL) allowed proper characterization.^15^ On the other hand, the ^13^C NMR spectra of **4NIPBSP-dt** could only be obtained by adding TFA-d and using a 700 MHz spectrometer due to its high tendency to aggregate (See ESI for further details). Unfortunately, the ^13^C NMR of both **4PIPBSP-dt** and **4PIPBSP-dipb** could not be properly acquired due to the large and fully fused nature of their structures. To assess stability, thermal analyses under nitrogen (Figures S26–S27) showed decomposition only above 400 °C and no phase transitions between 30–300 °C, confirming suitability for device fabrication. The primary challenge of this work lies in the synthesis and characterization of the tetrapodal-rylenimide derivatives presented in this article. We anticipate extending the π-conjugation in these 3D assemblies will significantly enhance not only their solubility and processability, but also their optical and electrochemical properties, making this strategy a promising approach for the development of new organic functional materials. In addition to these experimental efforts, we conducted a series of quantum-based calculations to investigate the chemical structure, geometry and key electronic properties of these compounds, as detailed below. The chemical structure of these three-dimensional semiconductors was optimized by density-functional theory (DFT) with the all-electron B3LYP/6-311G** functional basis set implemented in the Gaussian16 atomistic simulation package.^16^ As depicted in Figure 1a and Figure S28, all molecular analogues exhibit fully planar and conjugated structures. For the three-dimensional semiconductors, the planarity and conjugation are extended in comparison to their smaller counterparts, resulting in completely perpendicular arms due to the sp^3^-hybridized carbon in the spiro moiety, thereby forming three-dimensional X-shape structures. To rationalize possible molecular interactions, we theoretically predicted the formation of various dimeric structures at the all-electron B3LYP/6-311G** level, considering both parallel and antiparallel configurations (Figure 1b and Figures S28 and S29).

**Figure 1:**
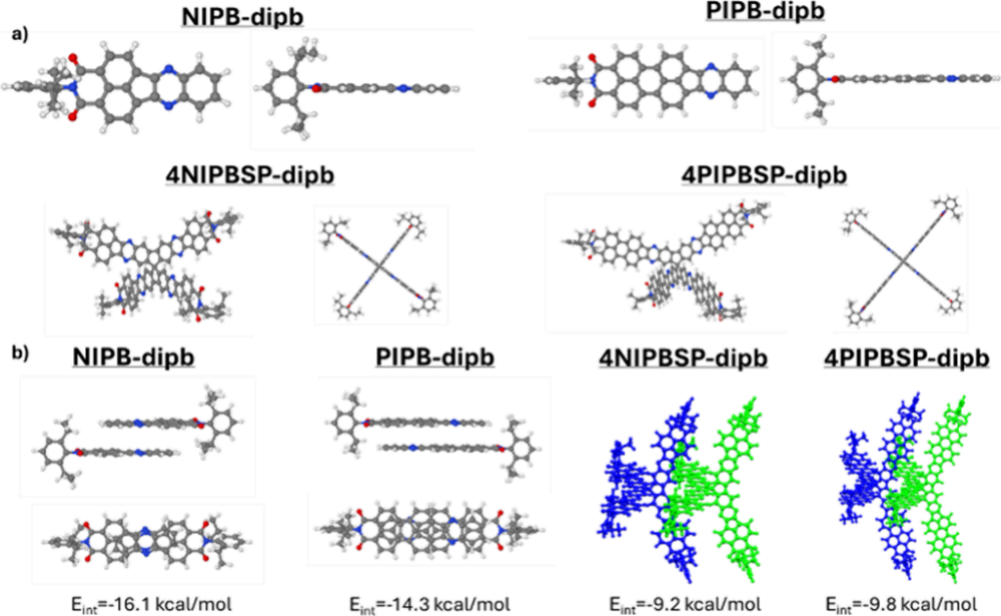
a) Optimized geometries and b) dimeric species for NIPB-dipb, PIPB-dipb, 4NIPBSP-dipb and 4PIPBSP-dipb.

Notably, significant differences were observed between the molecular analogues depending on the substitution of the nitrogen atom of the imide group. When the 2,6-diisopropylbenzene unit was used, only antiparallel conformations were predicted, with similar energy values for both **NIPB** and **PIPB** derivatives. However, when a longer and branched alkyl chain (2-decyltetradecyl) was introduced, both configurations were sufficiently stable due to van der Waals interactions between the alkyl chains.^17^ In this case, the antiparallel conformation was found to be the most stable.

This trend was also observed for the more extended **PIPB** derivative, which was more stable in the antiparallel conformation compared to the parallel one, likely due to the extended π-surface. These π-π interactions are consistent with experimental observations, reinforcing our hypothesis that effective chemical strategies must be developed to minimize such interactions when using π-extended semiconductors. To further investigate this hypothesis, we performed a battery of theoretical in silico experiments on the three-dimensional assemblies. Interestingly, no π-π interactions were detected due to the central spiro unit, with only lateral C–H-π interactions between the rylene units. Additionally, it is noteworthy that the presence of alkyl chains, as compared to the aryl unit, plays a crucial role in enhancing the stability of the dimers, presumably due to interactions between the alkyl chains, as shown in figure S31. This behavior aligns well with the strong aggregation effects observed during the characterization of the three-dimensional 2-decyltetradecyl-based semiconductors, in contrast to the aryl-based assemblies and the molecular analogues.

The study of the photophysical properties using UV–vis spectroscopy (Table S2, Figures 3, S34–S36) reveals the characteristic signatures of these pyrazine-based building blocks. In all cases, two main absorption bands are observed around 334 and 346 nm for **NIPB-dipb** and **NIPB-dt**, and three main bands centered around 464, 497, and 535 nm in the cases of **PIPB-dipb** and **PIPB-dt**. These bands correspond to the principal electronic transitions, as described for similar assemblies published by our group.^11^ As shown in Figure 2, we first compared the effect of introducing the 9,9’-spirobi­[fluorene] unit on the absorption and emission properties of these semiconductors, independent of the solubilizing chain attached to the nitrogen atom of the imide. As depicted in Figure 2a and 2b, the introduction of three-dimensionality results in more red-shifted λ^max^ values, 46 nm for **4NIPBSP** and 20 nm for **4PIPBSP**, compared to their corresponding one-dimensional molecular analogues, **NIPB** and **PIPB**. Additionally, a significant increase in the molar extinction coefficient (ε) is observed for the extended derivatives. Regarding the solubilizing pendant chains, the only substantial difference in solution occurs with the more extended materials, **4PIPBSP-dipb** and 4**PIPBSP-dt**, where the introduction of the bulkier 2,6-diisopropylbenzene unit prevents the formation of aggregated species at 10^–5^ M. This aggregation phenomenon occurs when the 2-decyltetradecyl alkyl chain is used. Due to the large, fully fused and π-extended structure of these materials, concentration-dependent experiments were conducted to assess whether these materials were prone to aggregation in solution, potentially altering their optical properties, as previously observed for other three-dimensional perylenimide assemblies.^9^ As shown in Figure S34, only **4PIPBSP-dt** showed evidence of aggregation within the selected concentration range, which is further supported by thin-film measurements and solvent-dependent experiments (see below and Figures S35 and S36). In addition to these experimental observations, we also performed a series of quantum-based theoretical calculations to predict the absorption properties of these semiconductors.

**Figure 2:**
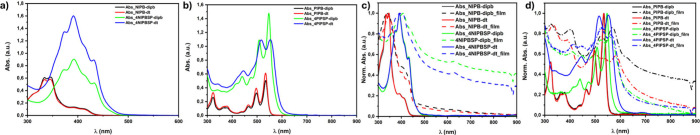
a,b) UV–vis absorption profiles in chloroform solutions and c,d) comparison between chloroform solution (solid) and thin-film (dashed) absorption spectra carried out for all mono and tridimensional semiconductors.

As shown in Figures S32 and S33, the predicted behavior of these organic architectures closely matches the experimental data, with similar trends in λ^max^ shifts corresponding to the chemical modifications made in each molecule. All absorption maxima are associated with HOMO→LUMO electronic transitions, following the same trends observed for similar three-dimensional analogues previously described by our group.^9,10^ To evaluate the influence of different nitrogen functionalization and potential aggregation in the solid state, thin-film absorption measurements were conducted using the drop-casting method, with the results shown in Figure 3c and 3d. For the naphthalimide-based materials **NPIB** and **4NIPBSP**, both core and alkyl chain engineering strategies prove effective in preventing undesirable aggregation phenomena in both solution and solid state. However, in the π-extended derivatives **PIPB** and **4PIPBSP** (Figure 2d), solid-state measurements reveal a clear inversion of the vibronic pattern for both 2-decyltetradecyl derivatives, **PIPB-dt** and **4PIPBSP-dt**. In contrast, the introduction of both three-dimensional core and bulky pendant chains results in nonaggregated species even in the solid state, as observed with **PIPB-dipb** and **4PIPBSP-dipb**, thus confirming our initial hypothesis. The results suggest that, although the 2,6- diisopropylbenzene chain induces significant steric hindrance that can inhibit aggregation, its effectiveness is limited in highly extended π-conjugated systems. In such cases, strong π-π interactions can overcome this steric barrier, as observed for **PIPB**, leading to reduced solubility, an issue already discussed in the characterization section.

Similarly, core modulation strategy encounters a comparable limitation: while it enables the synthesis of more extended architectures, the incorporation of long alkyl chains to improve processability can promote aggregation in solution, as evidenced in **4PIPBSP-dt** (Figure 2b). Therefore, by combining both strategies in **4PIPBSP-dipb**, undesired π-π interactions are effectively suppressed in both solution and solid state. This dual approach enhances the processability of these π-extended systems and facilitates a more accurate investigation of their photophysical properties. In addition to these studies, solvent-dependent experiments were carried out to assess the influence of solvent polarity on the optical properties of the 1D and 3D π-extended assemblies. As shown in Figure S35, all semiconductors exhibit variations in their molar extinction coefficient (ε) across the selected solvents, which correlate well with solvent polarity and the extent of solvent–solute interactions.^18^ A slightly bathochromic shift is observed with decreasing solvent polarity, indicating a reduced stabilization of the excited state in nonpolar environments. This behavior is consistent with that reported for other rylenimide-based systems,^19^ and, in this case, results in an inversion of the absorption maxima in toluene solutions. For **4NIPBSP-dt**, **4PIPBSP-dt** and **4PIPBSP-dipb**, their limited solubility in ethyl acetate leads to an inversion in the λ^max^ and red-shifted tails, suggesting the formation of strong aggregates in this polar medium. Finally, analysis of the emission spectra from the solvent-dependent emission experiments (Figure S37) reveals that significant polarity effects are observed only in the smallest derivatives, **NIPB-dipb** and **NIPB-dt**. In these cases, increasing solvent polarity induces both a hypsochromic shift and an inversion in the λ_em_^max^. This behavior is attributed to the high sensitivity of the excited states to the solvent environment,^20^ leading to notable changes in the emission profile due to the involvement of different possible electronic transitions, a phenomenon previously reported for other rylenimide semiconductors.^18^ In contrast, for the rest of the assemblies, only moderate hypsochromic shifts are detected upon increasing solvent polarity, as shown in Figure S37c-h, following similar trends to the smaller one-dimensional analogues. It is also worth noting that, in the case of the three-dimensional derivatives, both naphthalene and perylene imides exhibit a progressive loss of the structured emission observed in their smaller counterparts. From the cyclic voltammetry experiments (Figures 3a, S39–S46) the highest occupied molecular orbital (HOMO) and the lowest unoccupied molecular orbital (LUMO) of the fully fused π-extended rylenimide semiconductors were estimated. As shown in Figure 3b, the expansion of the conjugation through the 9,9’-spirobi­[fluorene] unit exerts a stronger influence on the HOMO than on the LUMO, a trend supported by DFT calculations (Figure 3b) and consistent with the electron-donating nature of this central core. In all cases, the HOMO is destabilized by at least 0.1 eV in the **4NIPBSP** derivatives, whereas for the **4PIPBSP** assemblies, the destabilization reaches up to 0.18 eV, presumably due to the more extended π-system of the perylenimide units (Table S3). However, in the smaller derivatives, this effective conjugation between the two naphthalimide moieties also impacts on the LUMO energy level, which is lower in the **4NIPBSP** compounds compared to the one-dimensional analogue **NIPB**. These results are in good agreement with the values and trends obtained from the DFT-based calculations, in which both the HOMO and the LUMO exhibit variations depending on the chemical modifications introduced in each semiconductor (Table S3). As shown in Figure 3c, in all molecular analogues, the HOMO and LUMO orbitals are delocalized across the entire π-conjugated backbone. In contrast, for the three-dimensional assemblies, the LUMO is localized on only one of the conjugated arms, while the HOMO remains delocalized over the whole structure, with a major contribution from the central core. Additionally, reorganization energies were calculated and are summed in Table S1. These values highlight the effectiveness of the synthetic strategies used in this work, which allow for fine control over the stabilization of both electrons and holes. Specifically, in relation to the extension of the π-conjugation in the molecular analogues, the π-extension enhances the reorganization energies, a trend that is also evident upon introducing three-dimensional structures.

**Figure 3:**
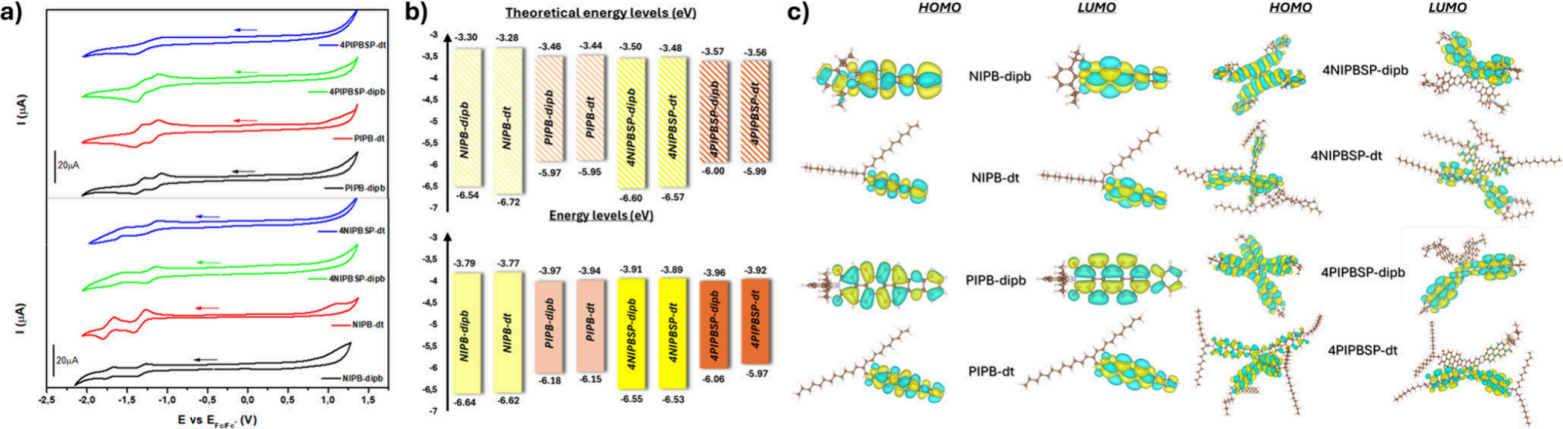
a) Cyclic voltammetry measurements for top: NIPB-dipb (black), NIPB-dt (red), 4NIPSP-dipb (green) and 4NIPSP-dt (blue) and bottom: PIPB-dipb (black), PIPB-dt (red), 4PIPSP-dipb (green) and 4PIPBSP-dt (blue), b) theoretical (top) and experimental (bottom) energy level diagrams and c) HOMO and LUMO topologies from the optimized geometries for all molecular analogues (left) and tridimensional semiconductors (right).

To sum up, we have synthesized a new class of fully fused three-dimensional rylenimide-based semiconductors that exhibit enhanced solubility, thermal stability, and tunable optoelectronic properties compared to other multidimensional rylenimide derivatives. The incorporation of a spiro-centered core and extended π-conjugation enables modulation of the frontier orbital energies, while, in addition to bulky side-chain functionalization at the imide nitrogen, both effectively suppress undesired π–π aggregation. Although cyclic voltammetry indicates independent redox centers with no direct intramolecular electronic communication upon reduction, the overall molecular design allows for fine-tuning of electronic structure and reorganization energies. These findings highlight a versatile strategy for the development of high-performance *n*-type organic materials suitable for solution-processed electronic devices.

## Supplementary Material



## Data Availability

The data underlying this study are available in the published article and its Supporting Information.

## Notes

Experimental details, ^1^H NMR, ^13^C NMR, FT-IR, HRMS characterization for all compounds, thermal analysis, photophysical measurements and theoretical calculations (PDF)
