# Supplementary material for: Giant Fully Fused Tetrapodal Rylenimides: Design, Synthesis and Optoelectrochemical Characterization via Alkyl Chain and Core Engineering Strategies
Source: Org Lett. 2025 Aug 25;27(35):9613–8. doi: 10.1021/acs.orglett.5c02655 (PMC12418488; doi:10.1021/acs.orglett.5c02655)
Supplement: Supplementary file 1 [file ol5c02655_si_001.pdf]

# Electronic Supporting Information

## Giant fully-fused tetrapodal rylenimides: Design, synthesis and optoelectrochemical characterization via alkyl chain and core engineering strategies.

*Matías J. Alonso-Navarro,[a,b,*] Fátima Suárez-Blas,[a,b] José Ignacio Martínez,[c] M. Mar Ramos[b] and José L. Segura.[a,*]*

[a]Matías J. Alonso-Navarro, Fátima Suárez-Blas and José L. Segura.

Department of Organic Chemistry, Complutense University of Madrid, Faculty of Chemistry.

Madrid 28040, Spain.

E-mail: segura@quim.ucm.es

[b]Matías J. Alonso-Navarro, Fátima Suárez-Blas and M. Mar Ramos.

Chemical and Environmental Technology Department. Univ. Rey Juan Carlos.

Móstoles 28933, Spain.

E-mail: matiasjesus.alonso@urjc.es

[c]José Ignacio Martínez.

Department of Low-dimensional Materials, Institute of Materials Science of Madrid (ICMM-CSIC),

Madrid 28049, Spain.

**Table of contents**

1. General information

2. Synthesis of compounds and characterization

3. DFT calculations

4. UV-Vis and Electrochemical data

5. References

## 1. General Information.

All the chemicals were purchased from commercial suppliers and used without further purification. Compounds **NID**,[1, 2] **PID**,[2, 3] **1**[4] and **2**[5] were obtained as previously described or with some modifications. $^1$H-NMR and $^{13}$C-NMR spectra were recorded on a Bruker Avance 300 MHz and 700 MHz spectrometers. Chemical shifts are reported in ppm and referenced to the residual non-deuterated solvent frequencies ($CDCl_3$: δ 7.26 ppm for $^1$H, δ 77.0 ppm for $^{13}$C, TFA-*d*: δ 11.50 ppm for $^1$H, δ 164.2 and 116.6 ppm for $^{13}$C). UV–vis absorption spectra of the compounds in HPLC chloroform, dichloromethane, ethyl acetate, toluene and THF solutions at 20 ºC were recorded on a Varian Cary 50 UV–vis spectrophotometer and fluorescence spectra were recorded on a Perkin-Elmer LS 55 fluorescence spectrometer. Mass spectra were recorded on a Bruker Reflex 2 (MALDI-TOF). FTIR spectra were carried out in a Shimadzu FTIR 8300 spectrophotometer. Cyclic voltammograms were recorded under an inert atmosphere using a Metrohm Autolab PGSTAT-302 electrochemical workstation at a scan rate of 100 $mV\cdot s^{-1}$ and a temperature of 20 °C. The measurements were performed in dichloromethane containing tetrabutylammonium hexafluorophosphate (TBAHFP, 0.1 $mol\cdot L^{-1}$) as the supporting electrolyte. A polymer-precoated platinum electrode was used as the working electrode, a platinum wire as the auxiliary electrode, and a $Ag/Ag^+$ electrode as the reference. All potentials were referenced to the ferrocene/ferrocenium ($Fc/Fc^+$) redox couple. Prior to each experiment, the working electrode was polished using a 0.05 µm alumina slurry on a polishing pad, rinsed thoroughly with deionized water and ethanol, and dried under a stream of argon. The electrolyte solutions were deoxygenated by bubbling high-purity argon through the solution for 15 minutes before measurements. All voltammograms are plotted following the IUPAC convention, where increasing potentials are shown toward the right on the x-axis and the starting point is 0 V.

## 2. Synthesis of compounds and characterization

General Procedure for the synthesis of the **NIPB** and **PIPB** compounds:

Over 10 mL of an anhydrous chloroform solution of an equimolar mixture (0,170 mmol) of **NID/PID** and 1,2-diaminobenzene was added under an argon atmosphere and a catalytic amount of p-TsOH (10%). The mixture was stirred at 50 ºC overnight in an aluminum bath and once the reaction time is complete, the reaction was cooled down and an excess of methanol was added until a yellow (NIPB) or red (PIPB) solid precipitates. The obtained solid was purified by chromatography column to give the corresponding products.

**NIPB-dt:** After column chromatography (100% Dichloromethane), we obtained 78mg of a pale-yellow solid (84% yield). The product's characterization is according to our previously published results.[6]

$^1$H-NMR (300 MHz, $CDCl_3$): δ (ppm) = 8.70 (d, J = 7.3 Hz, 2H), 8.50 (d, J = 7.3 Hz, 2H), 8.18 (dd, J = 6.3, 3.5 Hz,), 7.81 (dd, *J* = 6.3, 3.4 Hz,, 2H), 4.15 (m, 2H), 2.00 (m, 1H), 1.21 (m, 41H), 0.91 – 0.72 (m, 6H).

$^{13}$C-NMR (75 MHz, $CDCl_3$): δ (ppm) = 163.9, 154.3, 141.5, 132.8, 130.7, 130.2, 124.6, 122.7, 44.9, 36.9, 32.1, 31.9, 30.2, 29.88º, 29.82, 29.5, 26.7, 22.8, 14.3. (some aliphatic signals overlap).

**NIPB-dipb:** After column chromatography (100% Dichloromethane), we obtained 65 mg of a yellow solid (87% yield).

$^1$H-NMR (300 MHz, $CDCl_3$): δ (ppm) = 8.67 (d, J = 7.3 Hz, 2H), 8.46 (d, J = 7.3 Hz, 2H), 8.18 (dd, J = 6.3, 3.5 Hz, 2H), 7.81 (dd, J = 6.3, 3.4 Hz, 2H), 4.14 (d, J = 7.3 Hz, 2H), 2.04 – 1.95 (m, 1H), 1.21 (m, 40H), 0.87-0.83 (m, 6H).

$^{13}$C-NMR (75 MHz, $CDCl_3$): δ (ppm) = 163.6, 154.3, 145.9, 141.6, 137.5, 135.3, 133.4, 130.9, 130.3, 129.8, 124.5, 124.3, 122.9, 29.3, 24.2.

FTIR (ATR, $CHCl_3$): υ ($cm^{-1}$): 835, 1153, 1187, 1234, 1244, 1338, 1441, 1468, 1700, 1762, 1820, 2922.

HRMS (MALDI-TOF) m/z: [M+H]+ Calcd for $C_{32}H_{26}N_3O_2$ 484.2025; Found 484.2015

**PIPB-dt:** After column chromatography (100% Dichloromethane), we obtained 55 mg of a red solid (78% yield). The product's characterization is according to our previously published results.[3]

$^1$H-NMR (300 MHz, $CDCl_3$): δ (ppm) = 8.36 (d, J = 7.5 Hz, 2H), 8.18 (m, 4H), 8.01(d, J = 7.4 Hz, 2H), 7.87 (dd, J = 6.1, 3.4 Hz, 2H), 7.58 (dd, J = 6.2, 3.3 Hz, 2H), 4.00 (2H, d, J = 7.2 Hz), 1.96 (1H, m), 1.25 (40H, m), 0.84 (6H, m).

$^{13}$C-NMR (75 MHz, CDCl3): δ (ppm) = 163.0, 151.9, 139.9, 135.3, 133.5, 131.5, 130.5, 130.4, 129.1, 128.9, 128.6, 125.2, 123.2, 123.1, 121.9, 121.7, 121.0, 44.4, 36.8, 31.9, 36.1, 30.2, 29.7, 29.4, 26.4, 22.7, 14.1 (some aliphatic signals overlap).

**PIPB-dipb:** After column chromatography (100% Dichloromethane), we obtained 25 mg of a light red solid (84% yield).

$^1$H-NMR (300 MHz, $CDCl_3$): δ (ppm) = 8.75 (d, J = 7.9 Hz, 2H), 8.69 (d, J = 7.7 Hz, 2H), 8.65 (d, J = 7.9 Hz, 2H), 8.54 (d, J = 7.6 Hz, 2H), 8.24 (dd, J = 6.3, 3.6 Hz, 2H), 7.80 (dd, J = 6.3, 3.5 Hz, 2H), 7.50 (m, 1H), 7.36 (d, J = 7.8 Hz, 2H), 2.82 – 2.75 (m, 2H), 1.21-1.19 (m, 12H).

$^1$H-NMR (300 MHz, $CDCl_3$+TFA-*d*): δ (ppm) = 8.85 – 8.70 (m, 8H), 8.44 (dd, J = 6.3, 3.4 Hz, 1H), 7.99 (dd, J = 6.3, 3.3 Hz, 1H), 7.57 – 7.49 (m, 1H), 7.38 (d, J = 7.7 Hz, 1H), 2.74 (m, 2H), 1.20 (d, J = 6.8 Hz, 12H).

$^{13}$C-NMR (75 MHz, $CDCl_3$): δ (ppm) = 164.4, 159.8, 159.3, 145.7, 137.1, 135.3, 133.0, 132.6, 130.3, 128.5, 127.2, 127.0, 125.5, 124.6, 123.9, 123.2, 116.5, 112.7, 29.5, 24.1.

FTIR (ATR, $CHCl_3$): υ ($cm^{-1}$): 2961, 2871, 2371, 1921, 1855, 1559, 1469, 1319, 1062, 844, 587, 569.

HRMS (MALDI-TOF) m/z: [M+H]+ Calcd for $C_{42}H_{30}N_3O_2$ 608.2338; Found 608.2340

General Procedure for the synthesis of the **4NIPBSP** and **4PIPBSP** compounds:

Under Ar atmosphere, **NID** or **PID** (0,165 mmol), 4,4′,4″,4‴-Methanetetrayltetrakis[1,2-benzenediamine] octahydrochloride **1** (0,027 mmol) were suspended in 6 mL of dry chloroform, 3 mL EtOH and 0,3 mL glacial acetic acid. The solution is stirred at 60 ºC overnight in an aluminum bath and once the time has concluded, methanol is added, and the obtained precipitate was filtered and washed with water, methanol and hot methanol to acquire the corresponding solid.

**4NIPBSP-dt:** 52 mg of an orange solid (72% yield).

$^1$H-NMR (300 MHz, $CDCl_3$+TFA-*d*): δ (ppm) = 9.54 – 9.43 (bs, 4H), 8.98 – 8.73 (m, 12H), 8.51 (bs, 4H), 8.11 (bs, 4H), 4.28 – 4.12 (m, 8H), 2.06 – 1.87 (m, 4H), 1.39 – 1.10 (m, 196H), 0.93 – 0.81 (m, 24H).

$^{13}$C-NMR (75 MHz, $CDCl_3$+TFA-*d*): δ (ppm) = 164.5, 164.4, 153.8, 152.8, 143.3, 140.3, 135.8, 134.2, 134.0, 126.1, 125.4, 125.3, 122.1, 45.8, 38.3, 37.0, 32.1, 31.7, 31.4, 30.2, 29.9, 29.81, 29.78, 29.77, 29.5, 26.5, 22.8, 14.2.

FTIR (ATR, $CHCl_3$): υ ($cm^{-1}$): 2904, 2251, 1985, 1835, 1817, 1684, 1673, 1456, 1337, 1277, 1070, 744, 720.

HRMS (MALDI-TOF) m/z: [M]+ Calcd for $C_{177}H_{220}N_{12}O_8$ 2641.7177; Found 2641.7116

**4NIPBSP-dipb:** 46 mg of a yellow precipitate (87% yield).

$^1$H-NMR (300 MHz, $CDCl_3$): δ (ppm) = 9.03 (s, 4H), 8.86 (d, J = 7.4 Hz, 4H), 8.70 (d, J = 7.4, 4H), 8.68 (d, J = 7.4, 4H) 8.35 (d, J = 7.4 Hz, 8H), 7.89 (s, 4H), 7.56 – 7.41 (m, 4H), 7.34-7.32 (m, 8H), 2.77 (m, 8H), 1.20 – 1.06 (m, 48H)

$^{13}$C-NMR (75 MHz, $CDCl_3$): δ (ppm) = 163.5, 155.1, 154.5, 152.3, 145.8, 142.9, 142.6, 142.5, 137.1, 137.0, 135.6, 133.5, 133.3, 130.7, 129.9, 126.4, 126.2, 124.8, 124.7, 124.3, 123.3, 122.8, 122.7, 77.2, 29.3, 24.1.

FTIR (ATR, $CHCl_3$): υ ($cm^{-1}$): 2947, 2859, 2099, 1973, 1820, 1709, 1607, 1549, 1446, 1335, 1228, 1156, 1056, 838, 713, 560.

HRMS (MALDI-TOF) m/z: [M+H]+ Calcd for $C_{129}H_{93}N_{12}O_8$ 1937.7239; Found 1937.7244

**4PIPBSP-dt:** 55 mg of a red precipitate (65% yield).

$^1$H-NMR (300 MHz, $CDCl_3$+TFA-*d*): δ (ppm) = 9.76 – 9.61 (bs, 4H), 9.06 – 8.71 (m, 32H), 8.29 (bs, 4H), 4.34 – 4.19 (m, 8H), 2.57 – 2.39 (m, 4H), 1.44 – 1.07 (m, 164H), 0.98 – 0.76 (m, 12H), 0.23 – 0.05 (m, 24H).

FTIR (ATR, $CHCl_3$): υ ($cm^{-1}$): 2914, 2200, 1912, 1765, 1674, 1588, 1519, 1464, 1352, 1148, 1242, 1081, 840, 744, 628.

HRMS (MALDI-TOF) m/z: [M+H]+ Calcd for $C_{217}H_{237}N_{12}O_8$ 3138.8507; Found 3138.8421

**4PIPBSP-dipb:** 47 mg of a light red precipitate (71% yield).

$^1$H-NMR (300 MHz, $CDCl_3$+TFA-*d*): δ (ppm) = 9.62 (bs, 4H), 9.14 – 8.66 (m, 32H), 8.21 (bs, 4H), 7.55 – 7.49 (m, 4H), 7.35 (d, J = 7.8 Hz, 8H), 2.65 (m, 8H), 1.30 – 1.09 (m, 48H).

FTIR (ATR, $CHCl_3$): υ ($cm^{-1}$): 2936, 2315, 2058, 1976, 1935, 1830, 1690, 1494, 1410, 1579, 1347, 1245, 1163, 1082, 833, 735, 614.

MALDI-HRMS (m/z): calculated for $C_{169}H_{108}N_{12}O_8$: 2432.8413, found ($M^+$ +1): 2433.8406

HRMS (MALDI-TOF) m/z: [M+H]+ Calcd for $C_{169}H_{109}N_{12}O_8$ 2433.8491; Found 2433.8406

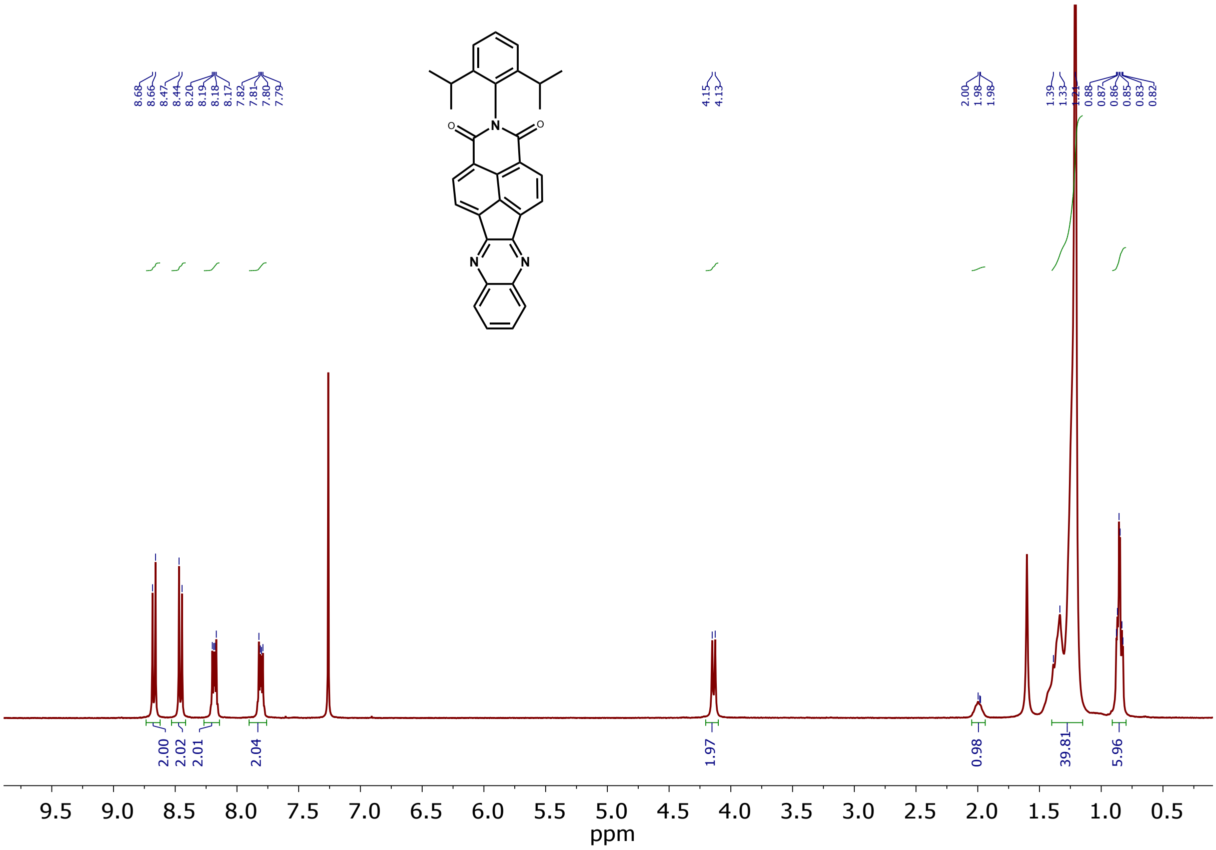

**Figure S1.** $^{1}$H-NMR spectrum (300 MHz) of **NIPB-dt** in $CDCl_3$

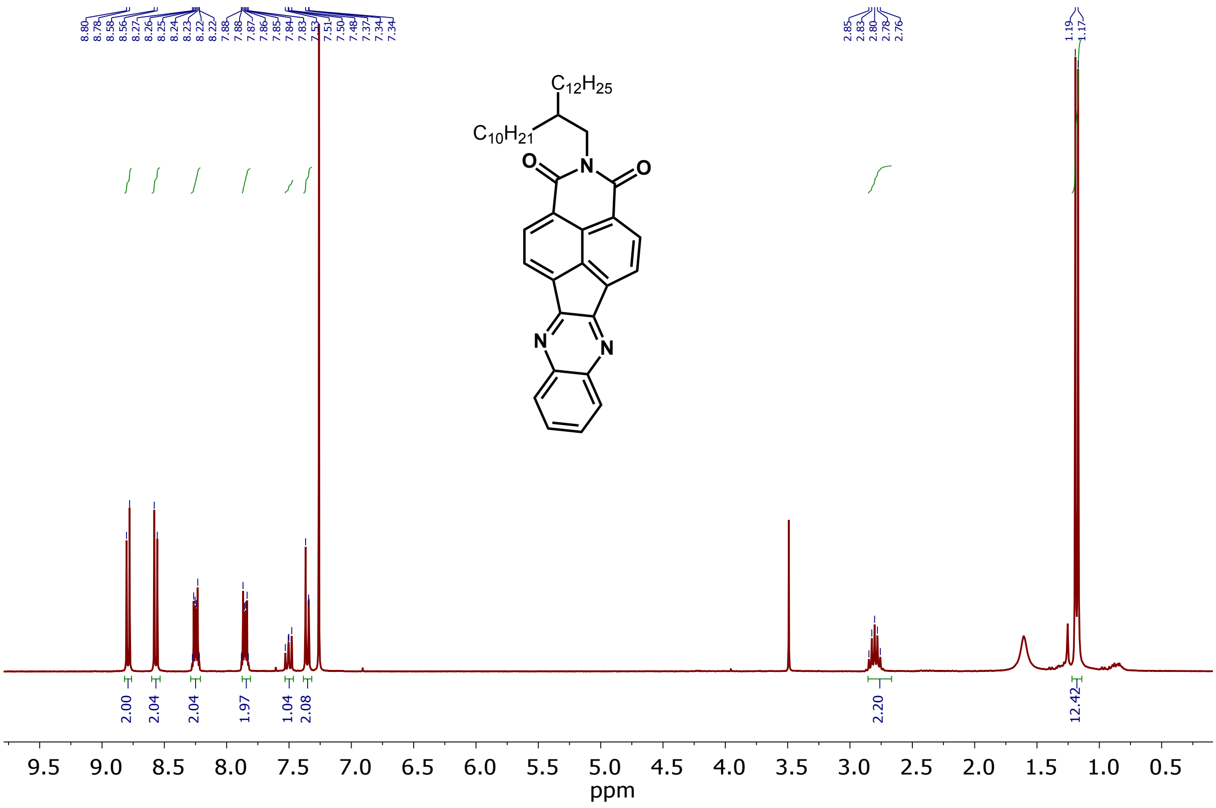

**Figure S2.** $^{1}$H-NMR spectrum (300 MHz) of **NIPB-dipb** in $CDCl_3$

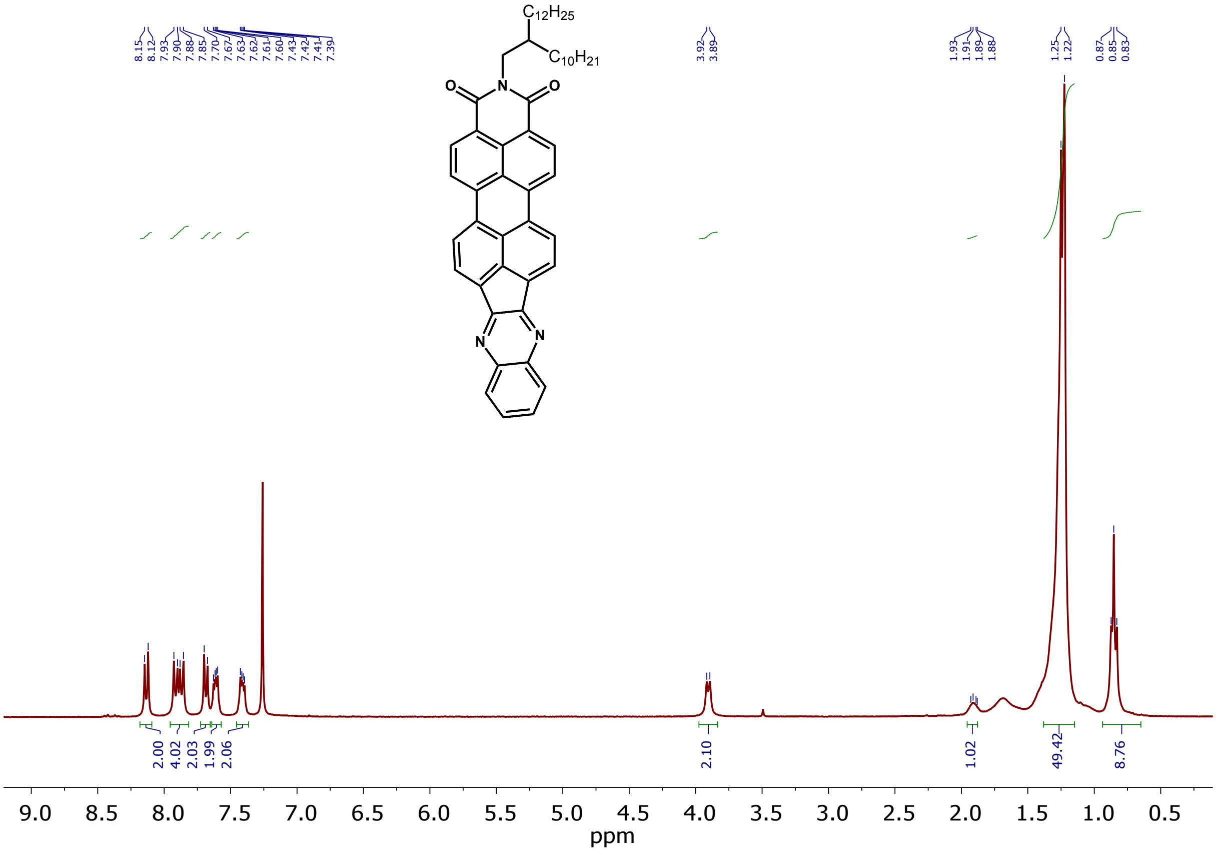

**Figure S3.** $^{1}$H-NMR spectrum (300 MHz) of **PIPB-dt** in $CDCl_3$

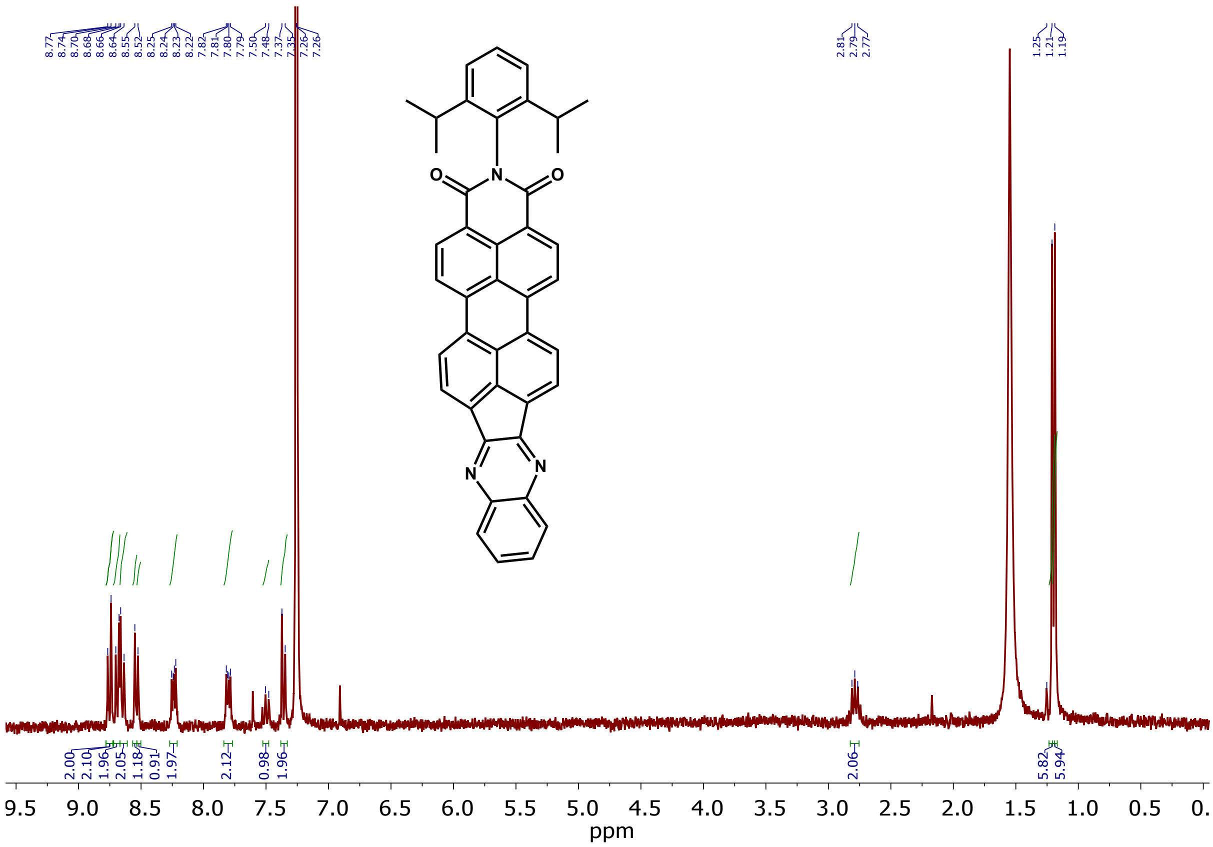

**Figure S4.** $^{1}$H-NMR spectrum (300 MHz) of **PIPB-dipb** in $CDCl_3$

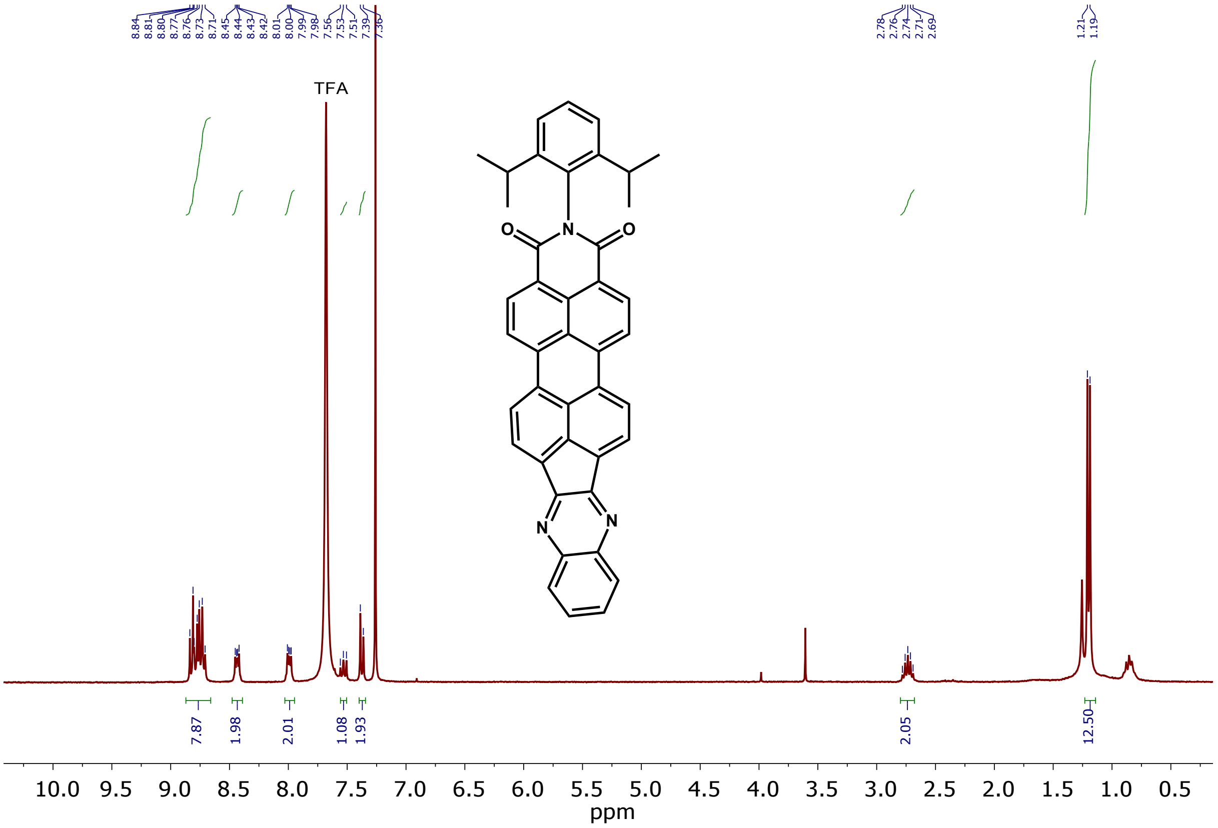


**Figure S5.** $^{1}$H-NMR spectrum (300 MHz) of **PIPB-dipb** in $CDCl_3$+TFA-*d*

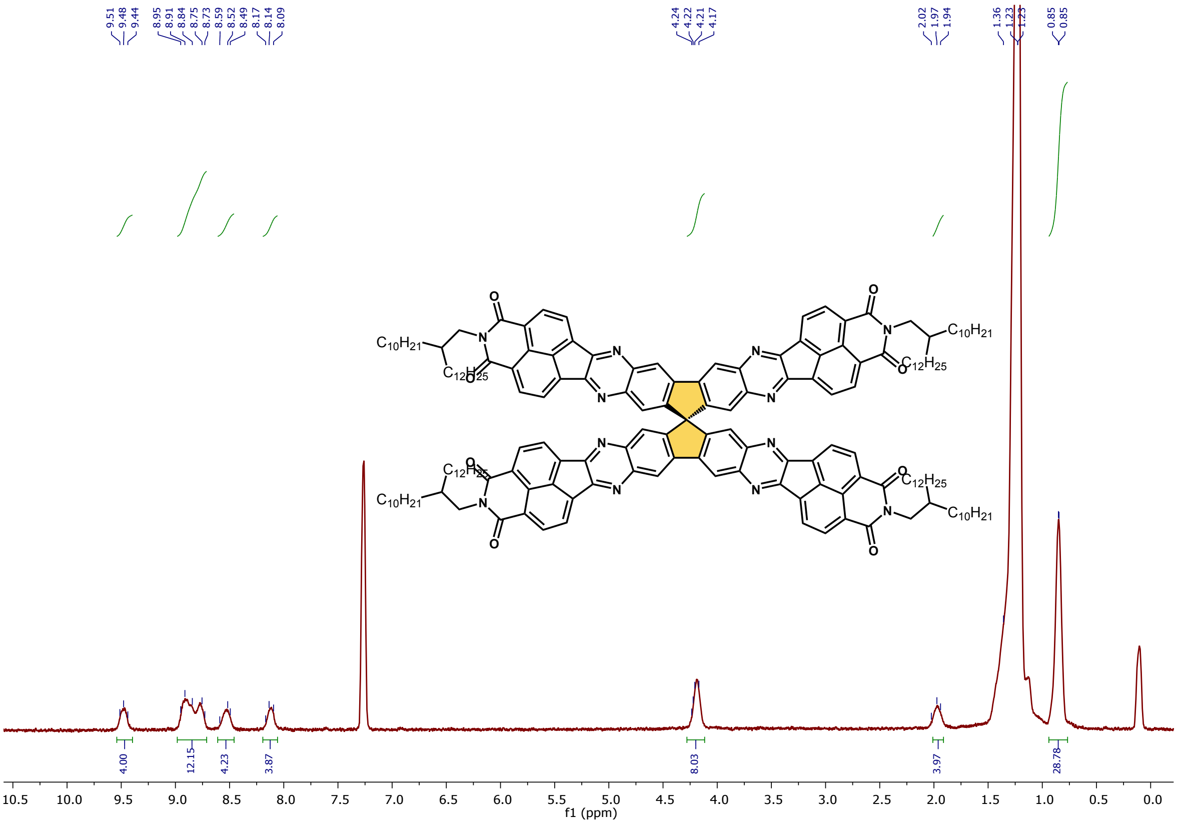


**Figure S6.** $^{1}$H-NMR spectrum (700 MHz) of **4NIPBSP-dt** in $CDCl_3$+TFA-*d*.

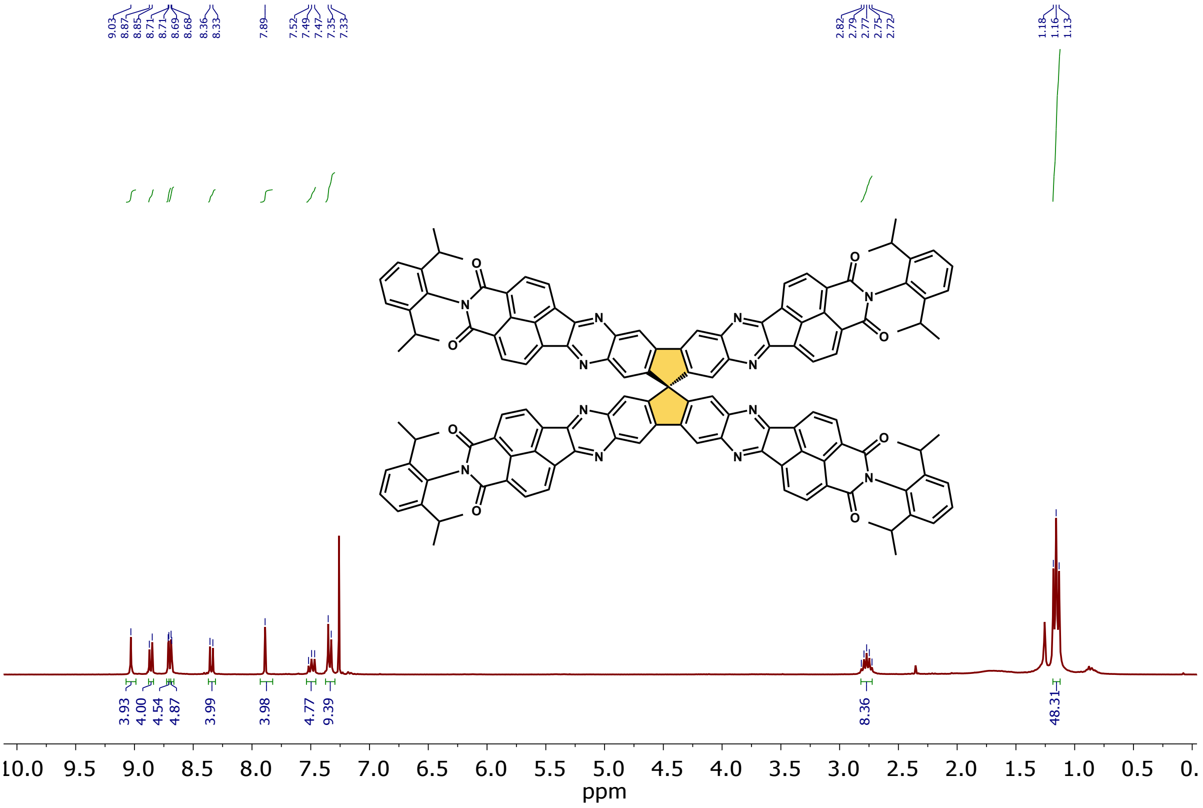

**Figure S7.** $^{1}$H-NMR spectrum (300 MHz) of **4NIPBSP-dipb** in $CDCl_3$

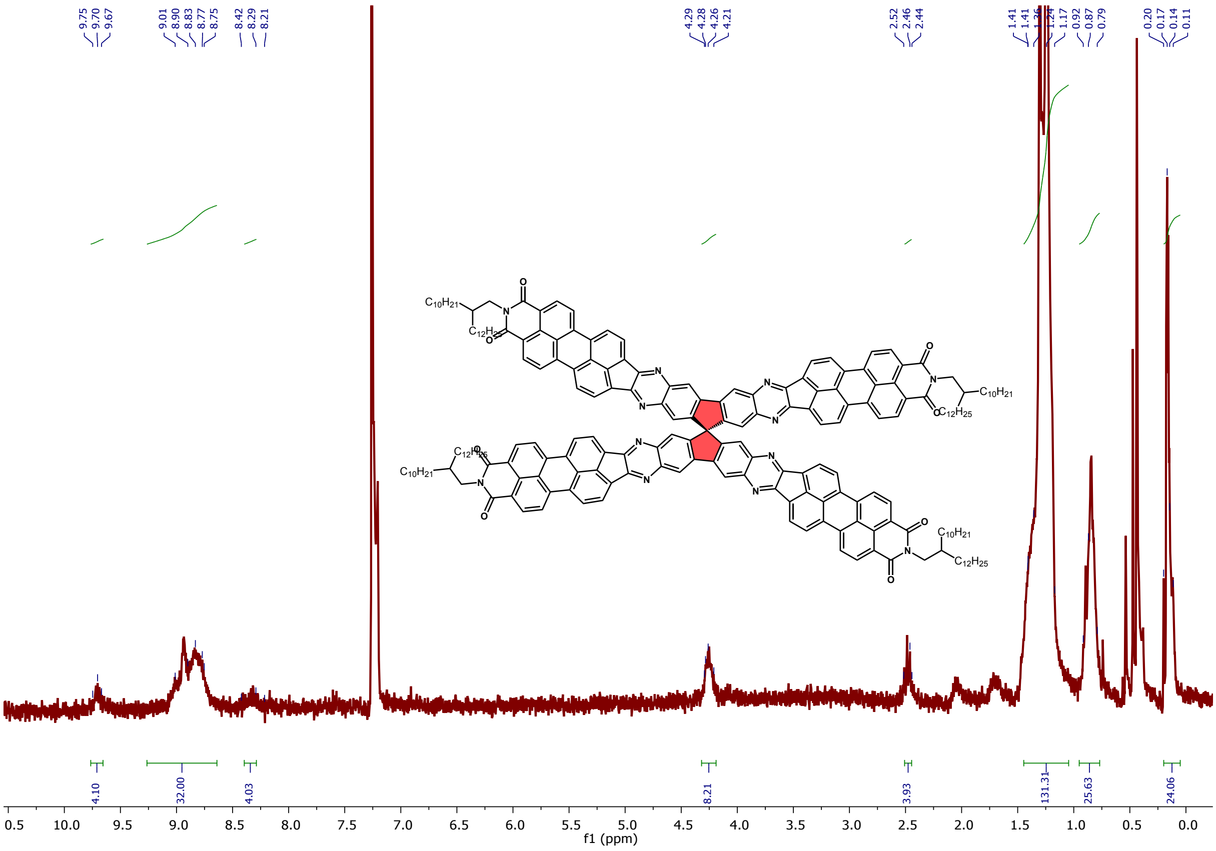

**Figure S8.** $^{1}$H-NMR spectrum (300 MHz) of **4PIPBSP-dt** in $CDCl_3$+TFA-*d*.

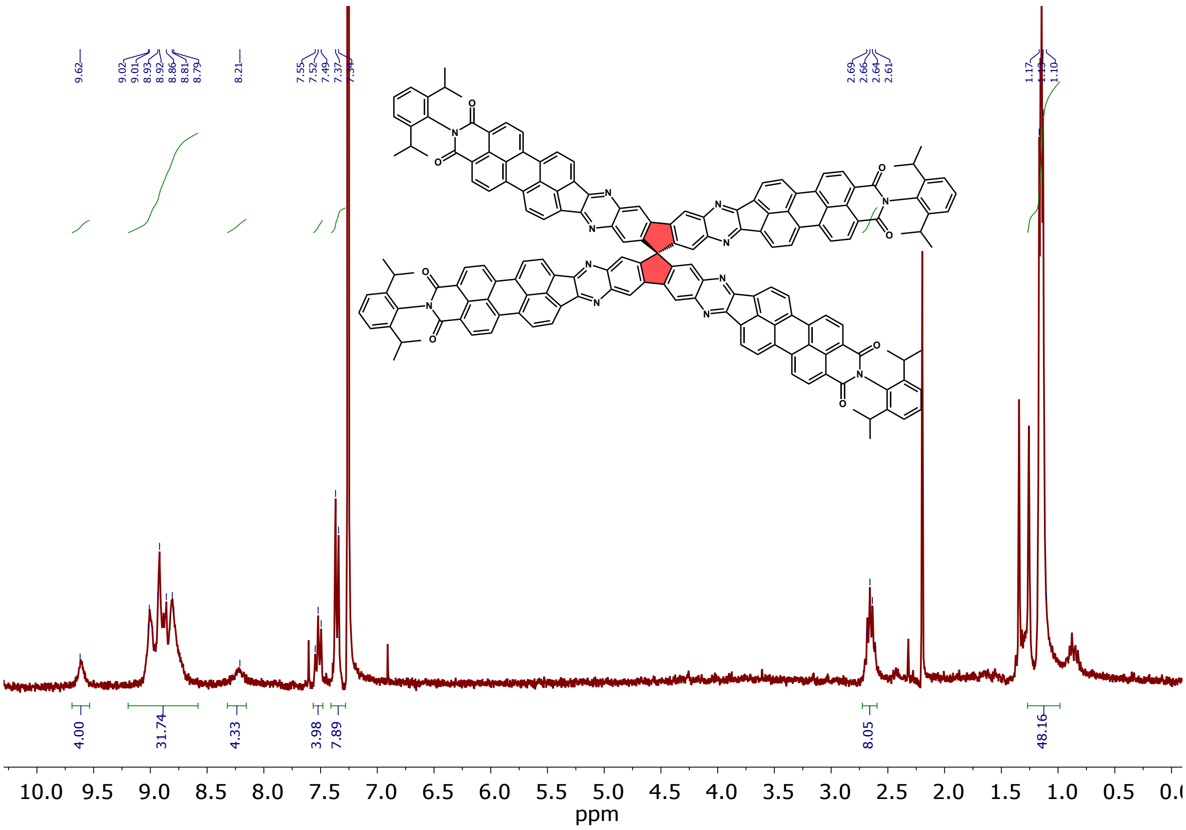


**Figure S9.** $^1$H-NMR spectrum (300 MHz) of **4PIPBSP-dipb** in $CDCl_3$+TFA-*d*.

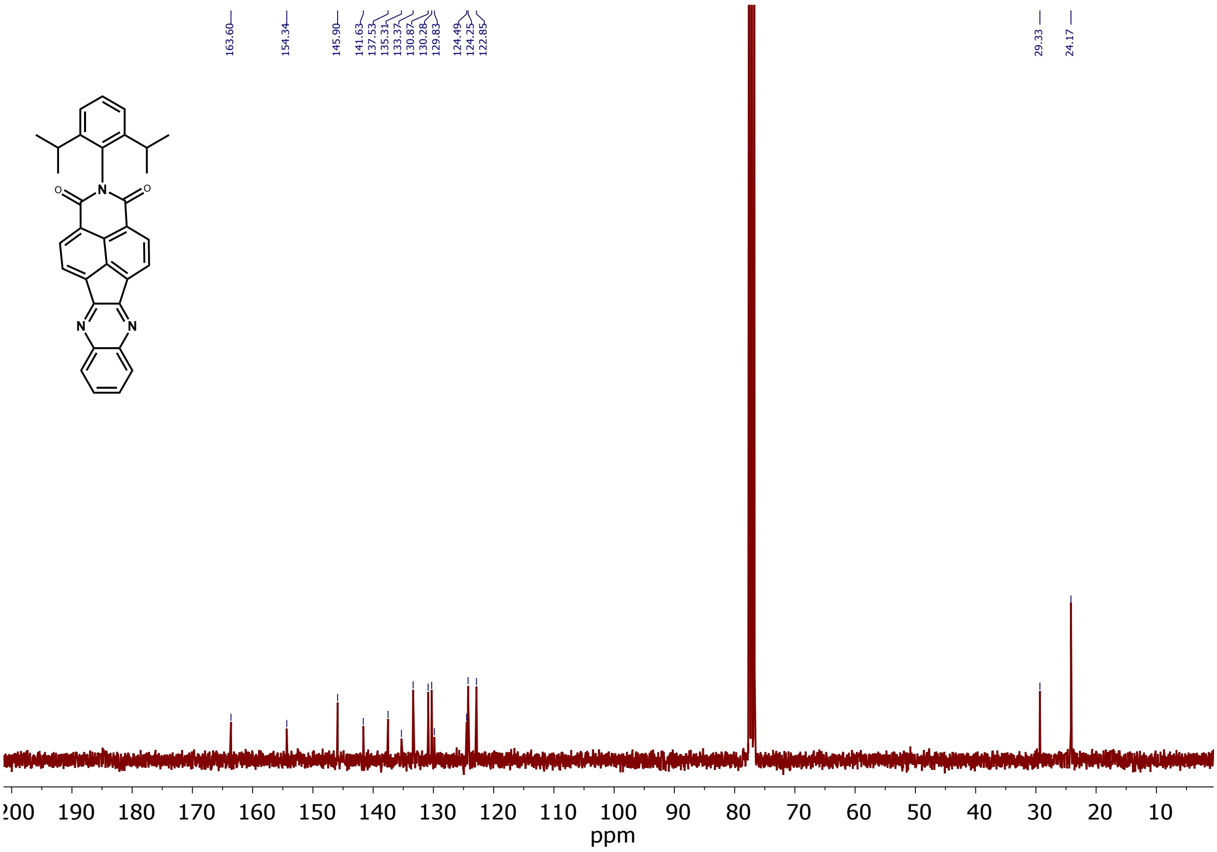


**Figure S10.** $^{13}$C-NMR spectrum (75 MHz) of **NIPB-dipb** in $CDCl_3$

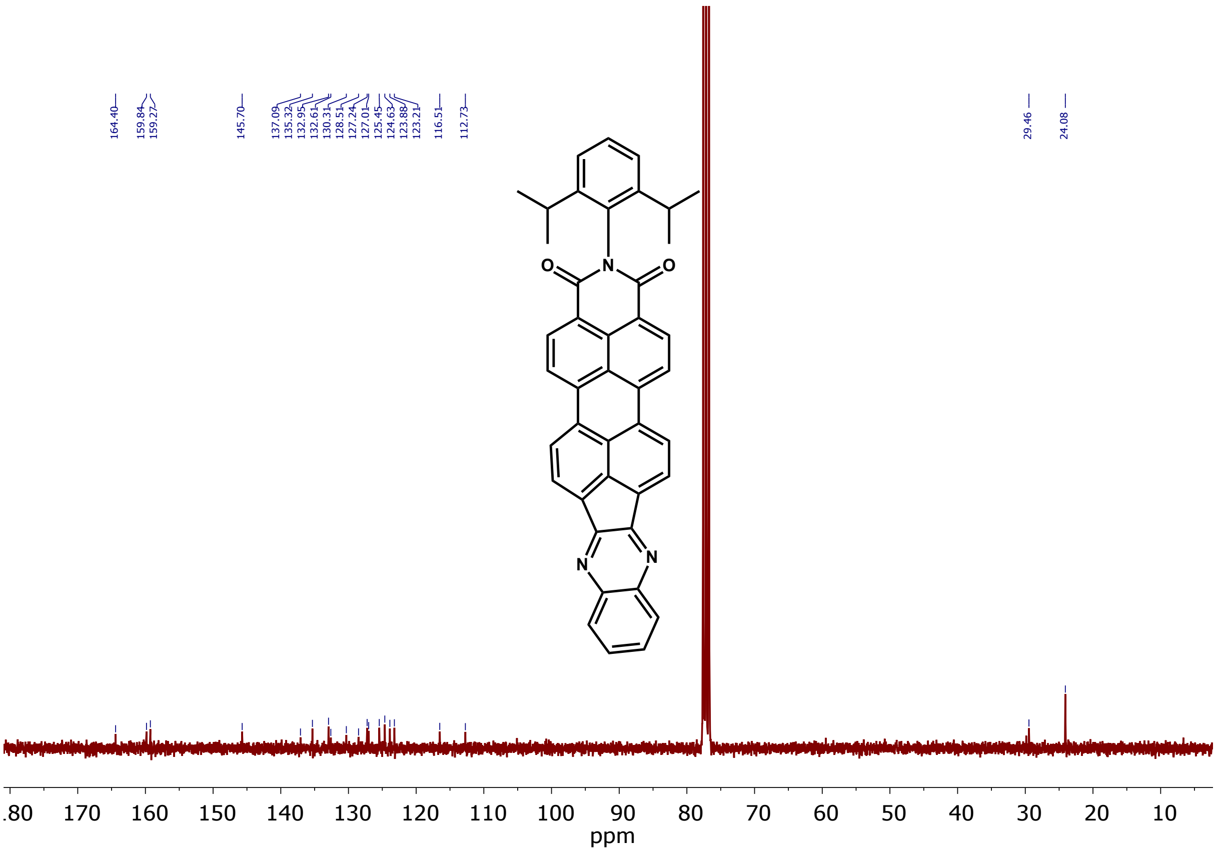


**Figure S11.** $^{13}$C-NMR spectrum (75 MHz) of **PIPB-dipb** in $CDCl_3$

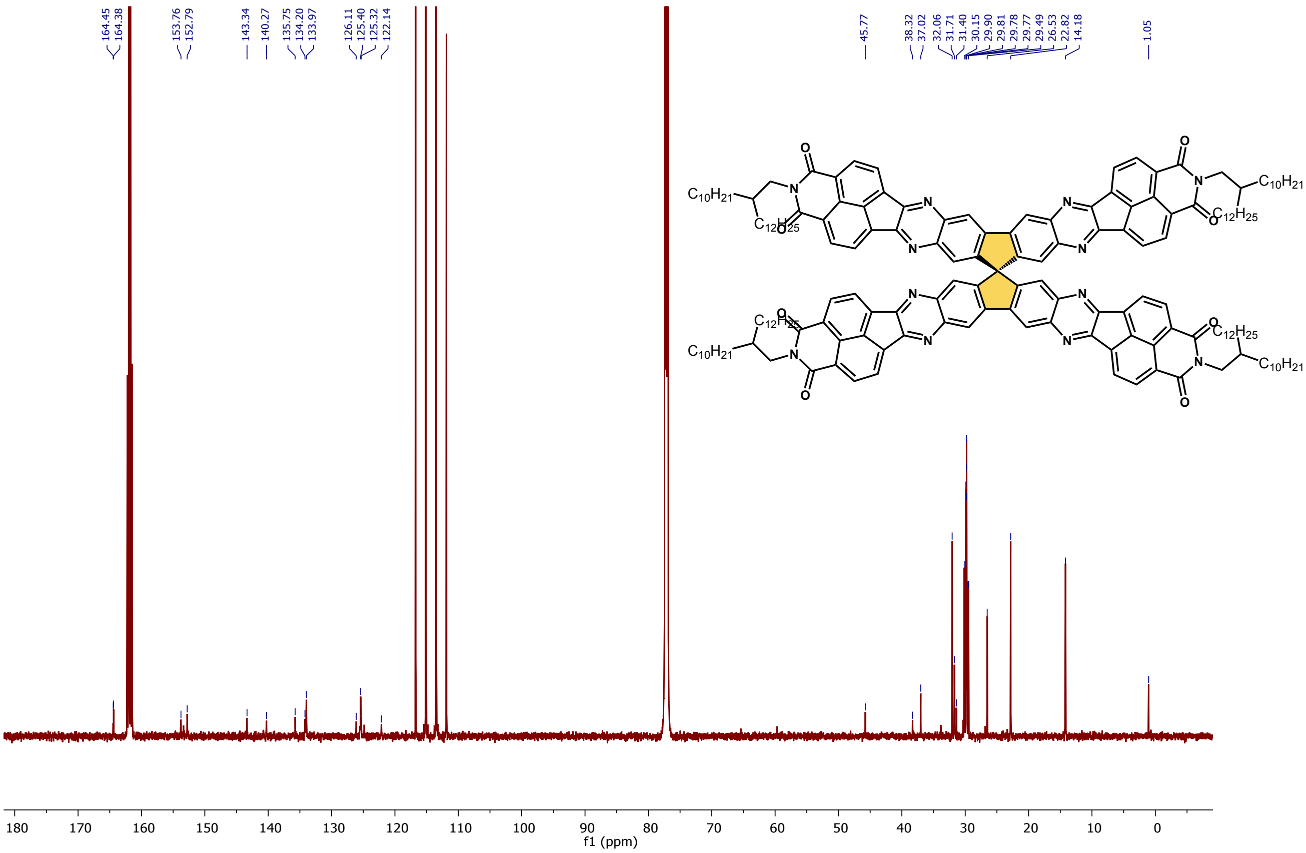


**Figure S12.** $^{13}$C-NMR spectrum (125 MHz) of **4NIPBSP-dt** in $CDCl_3$+TFA-*d*

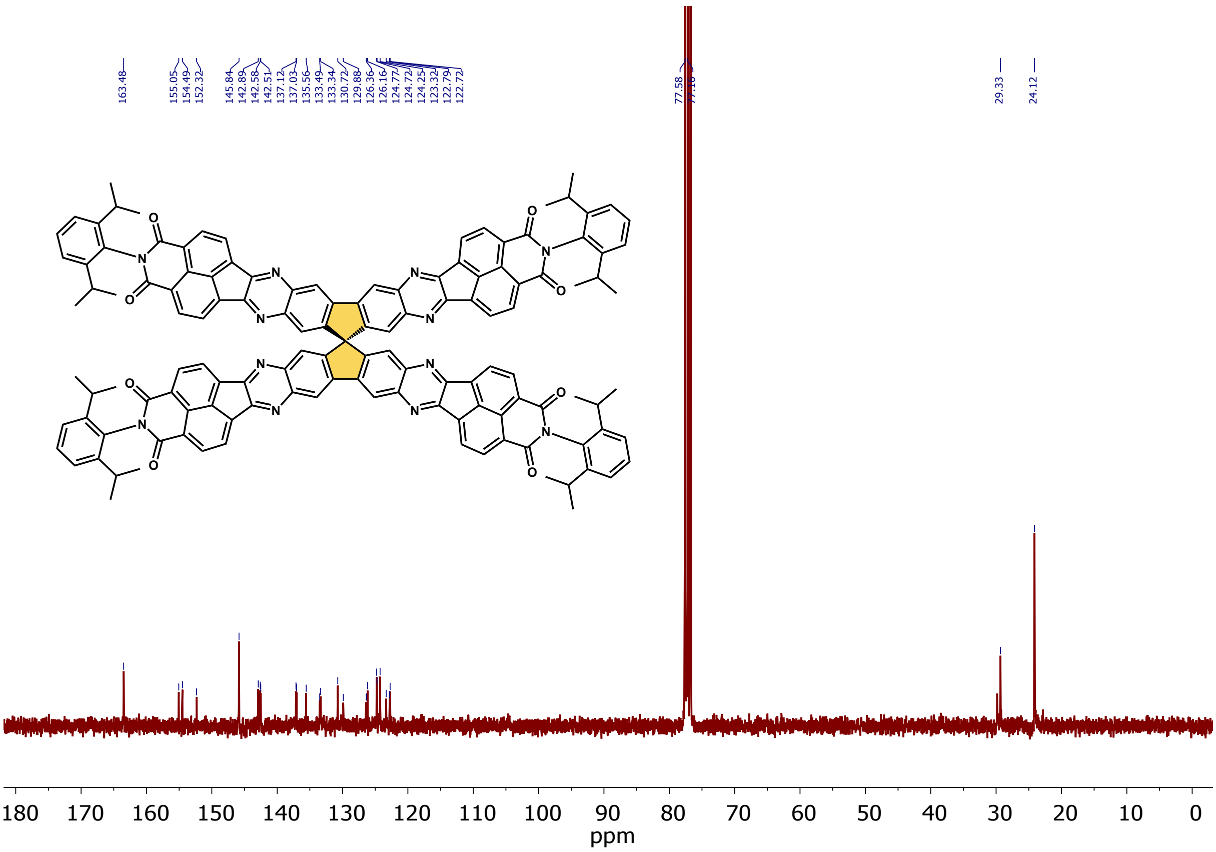


**Figure S13.** $^{13}$C-NMR spectrum (75 MHz) of **4NIPBSP-dipb** in $CDCl_3$

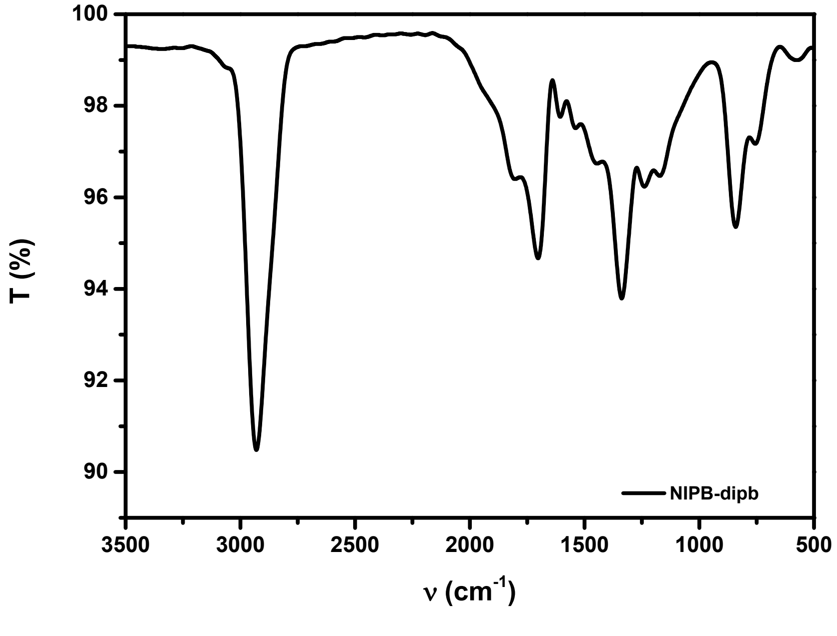


**Figure S14.** IR spectrum of **NIPB-dipb**

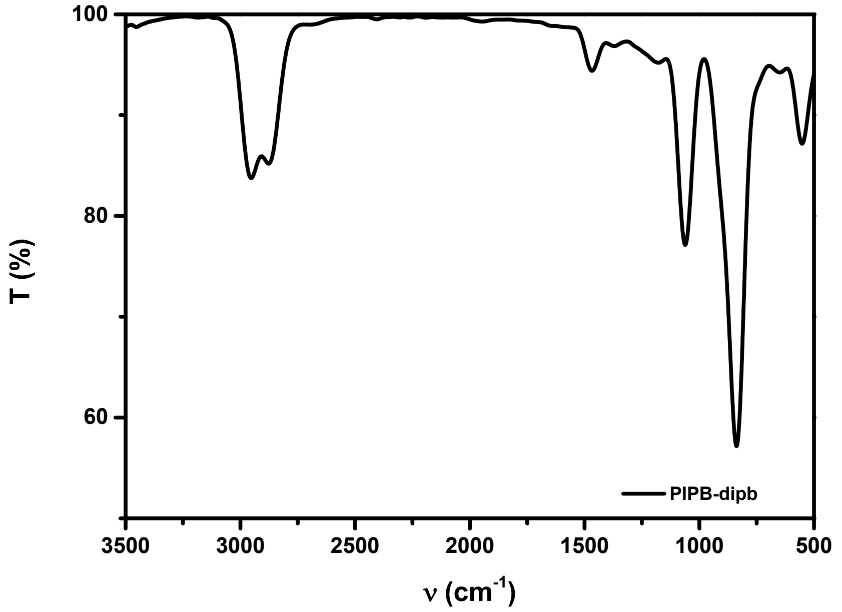


**Figure S15.** IR spectrum of **PIPB-dipb**

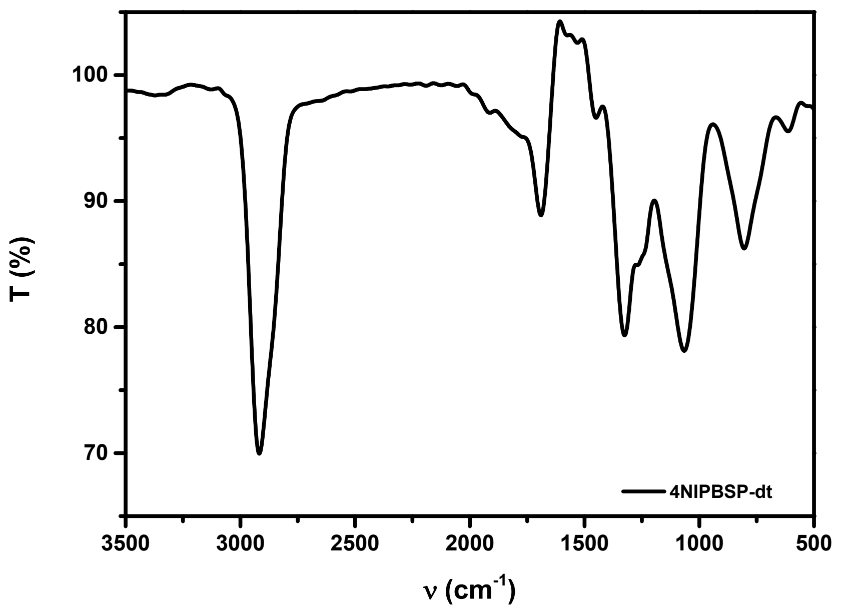


**Figure S16.** IR spectrum of **4NIPBSP-dt**

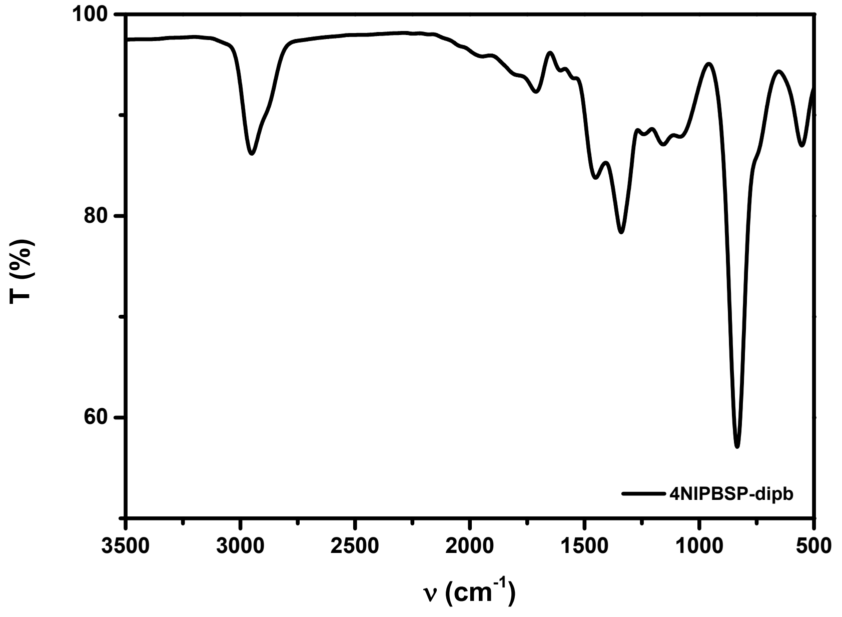


**Figure S17.** IR spectrum of **4NIPBSP-dipb**

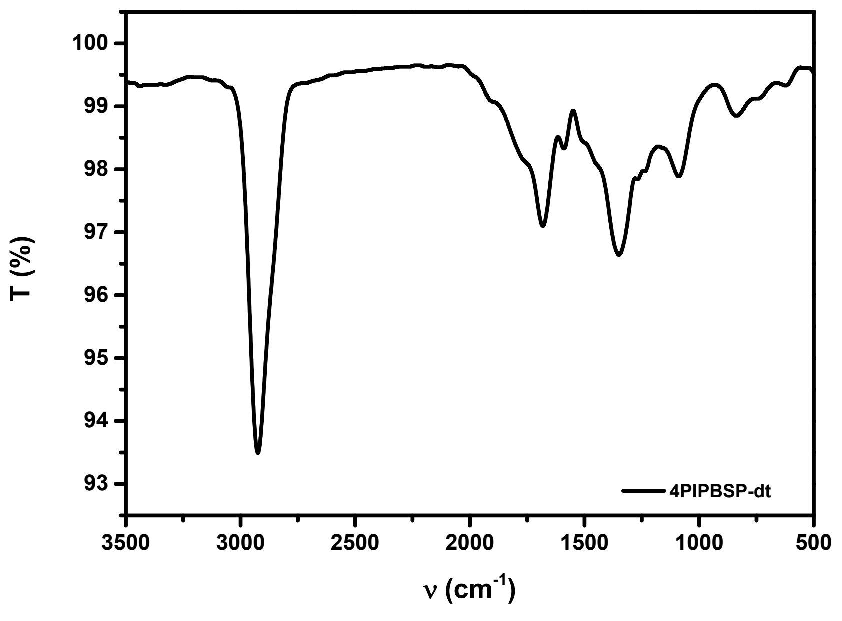


**Figure S18.** IR spectrum of **4PIPBSP-dt**

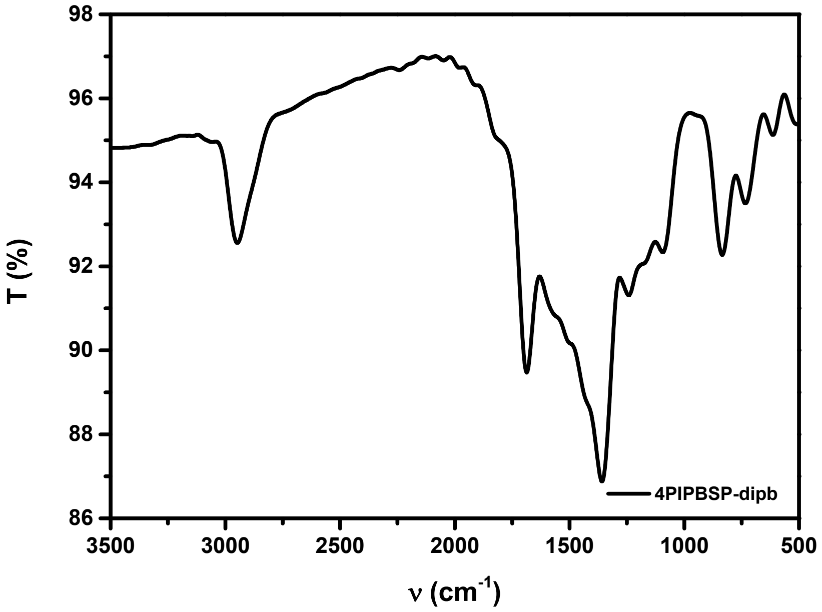


**Figure S19.** IR spectrum of **4PIPBSP-dipb**

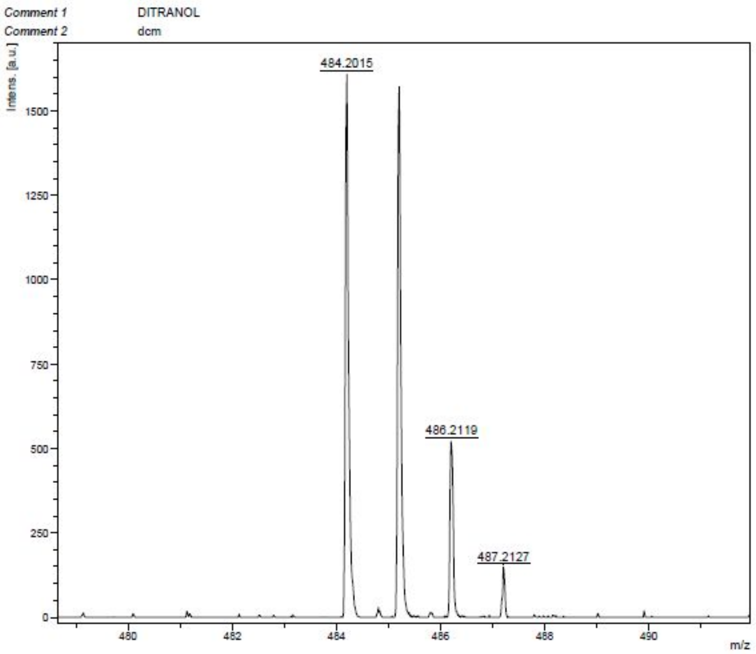


**Figure S20.** MALDI-HRMS (m/z) spectrum of **NIPB-dipb**

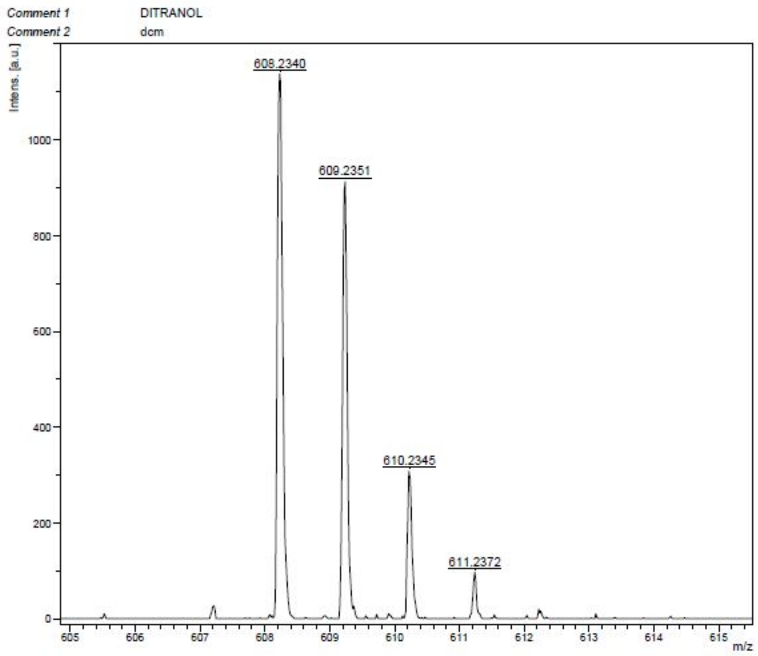


**Figure S21.** MALDI-HRMS (m/z) spectrum of **PIPB-dipb**

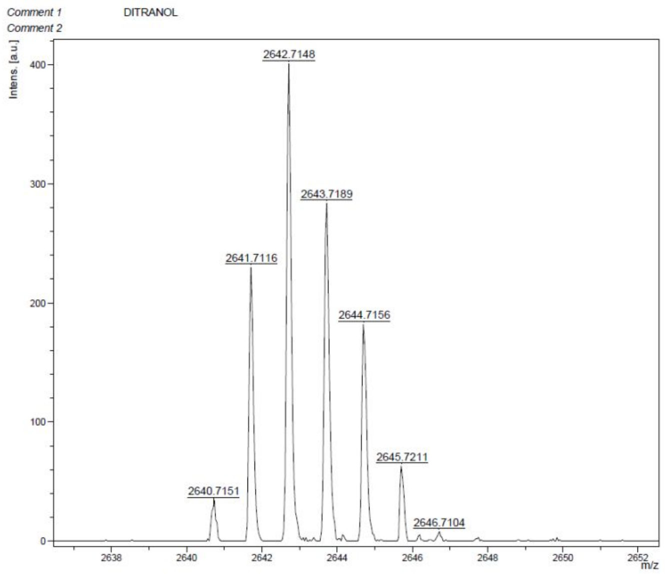


**Figure S22.** MALDI-HRMS (m/z) spectrum of **4NIPBSP-dt**

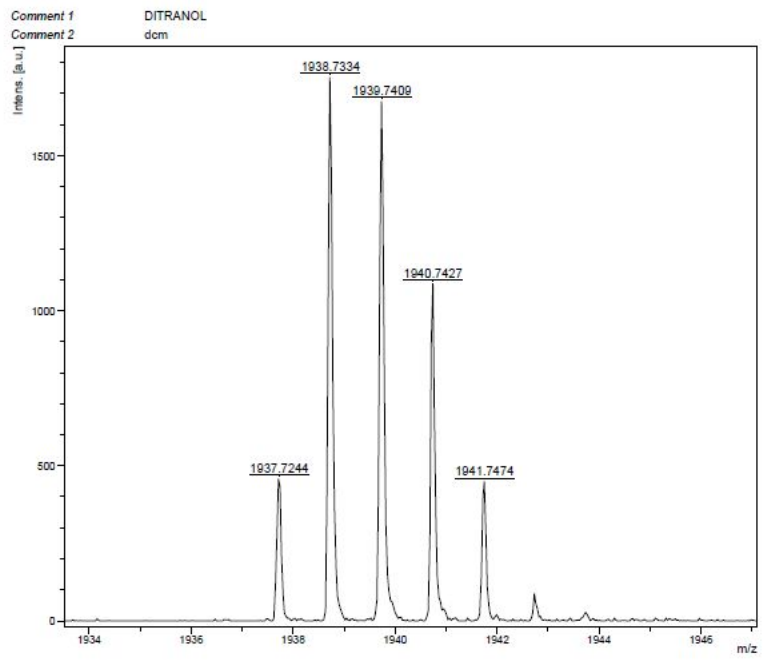


**Figure S23.** MALDI-HRMS (m/z) spectrum of **4NIPBSP-dipb**

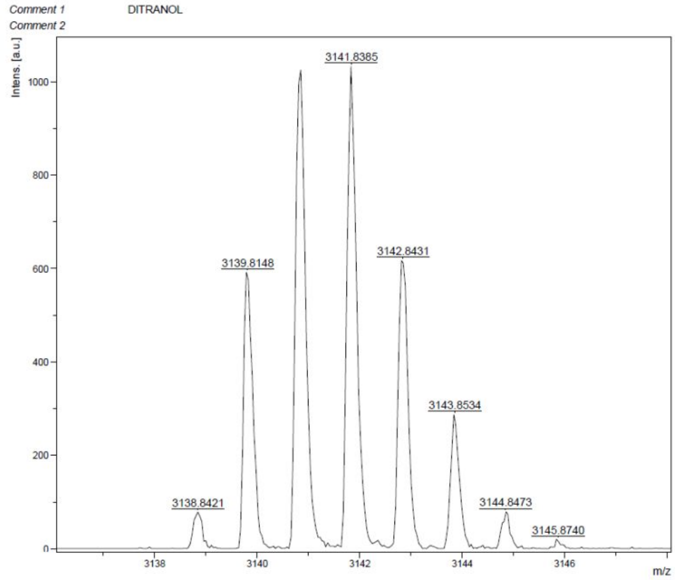


**Figure S24.** MALDI-HRMS (m/z) spectrum of **4PIPBSP-dt**

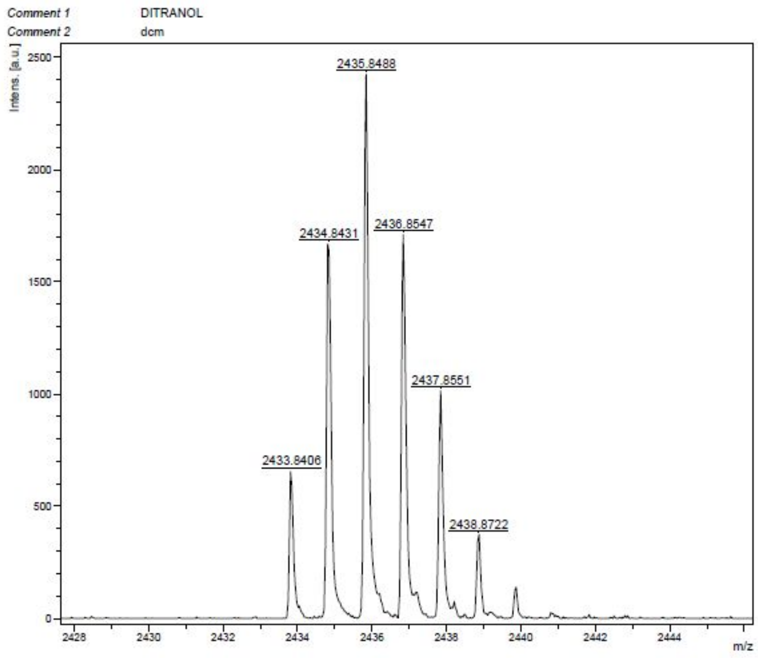


**Figure S25.** MALDI-HRMS (m/z) spectrum of **4PIPBSP-dipb**

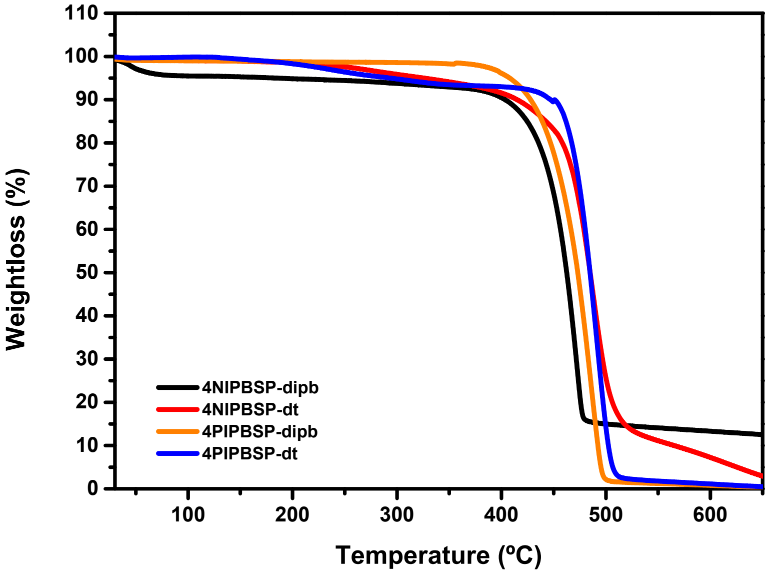


**Figure S26.** Thermogravimetric analysis of 4NIPBSP-dipb (black) and 4NIPBSP-dt (red) and 2) 4PIPBSP-dipb (blue) and 4PIPBSP-dt (green).

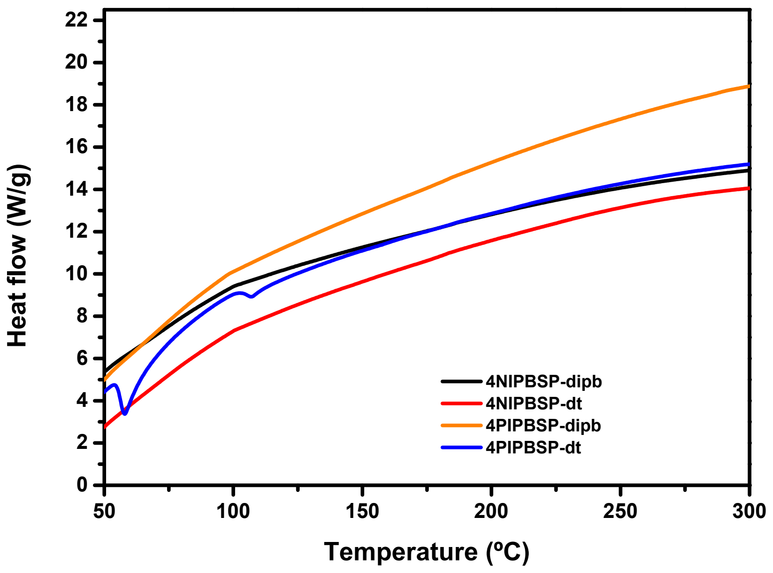


**Figure S27.** differential scanning calorimetry analysis of 4NIPBSP-dipb (black) and 4NIPBSP-dt (red) and 2) 4PIPBSP-dipb (blue) and 4PIPBSP-dt (green).

## 3. DFT calculations

Density functional calculations were carried out utilizing the B3LYP functional[7] and the 6-311G** basis set[8] as implemented in the Gaussian16 atomistic simulation package.[9] Time-dependent DFT (TDDFT) calculations[10-12] at the B3LYP/6-311G** level of theory were subsequently conducted on the optimized geometries to gain insights into their absorption spectra. For all structures under examination, the twenty lowest-energy electronic excited states were computed. The internal reorganization energies for both electron and hole transport in this semiconductor series were determined following previously described methods.[13] The intramolecular reorganization energy, a parameter reflecting the structural adjustments necessary to accommodate added charge, was computed, with an emphasis on achieving small reorganization energies as a prerequisite for efficient charge transport.

<u>XYZ cartesian coordinates</u> (in Å) of the fully optimized NIP-dipb, NIP-dt, PIPB-dipb, PIPB-dt, 4NIPBSP-dipb, 4NIPBSP-dt, 4PIPBSP-dipb and 4PIPBSP-dt molecular systems, and the resulting total energy (in atomic units) at the end of the ESI.

**NOTE:** The computation of normal modes for the optimized structures confirms that the NIP-dipb, NIP-dt, PIPB-dipb, and PIPB-dt configurations exhibit no imaginary frequencies, indicating that these structures correspond to true minima on the potential energy surface. In contrast, the 4NIPBSP-dipb and 4PIPBSP-dipb structures each display a single imaginary frequency, while the largest systems, the 4NIPBSP-dt and 4PIPBSP-dt, present two imaginary frequencies each.

However, in all cases, the imaginary frequencies are shallow, with magnitudes smaller than -50 cm⁻¹. Such low-magnitude imaginary modes are commonly attributed to numerical artifacts or to very shallow distortions along soft vibrational coordinates, such as torsions or collective low-frequency motions. These effects are particularly prevalent in large, flexible molecular systems with reduced symmetry, like the largest systems studied here, where aliphatic side chains, particularly in the 4NIPBSP-dt and 4PIPBSP-dt structures, introduce numerous loosely coupled degrees of freedom. Therefore, the presence of these small imaginary frequencies does not compromise the identification of the optimized structures as near-minima and can be reasonably accepted in the context of high-complexity systems. Computation of normal modes for the dimeric systems was computationally unfeasible (some of the configurations involved more than 800 atoms).

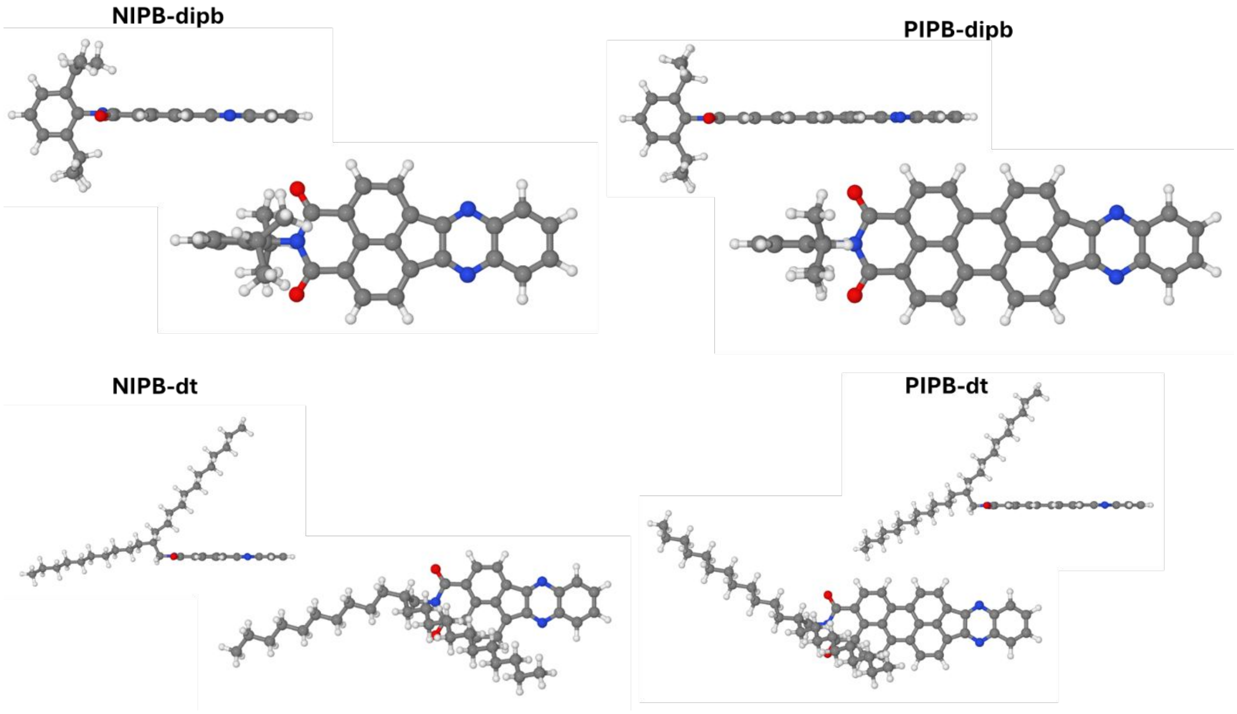


**Figure S28.** All-electron B3LYP/6-311G** optimized structures for NIPB and PIPB derivatives.

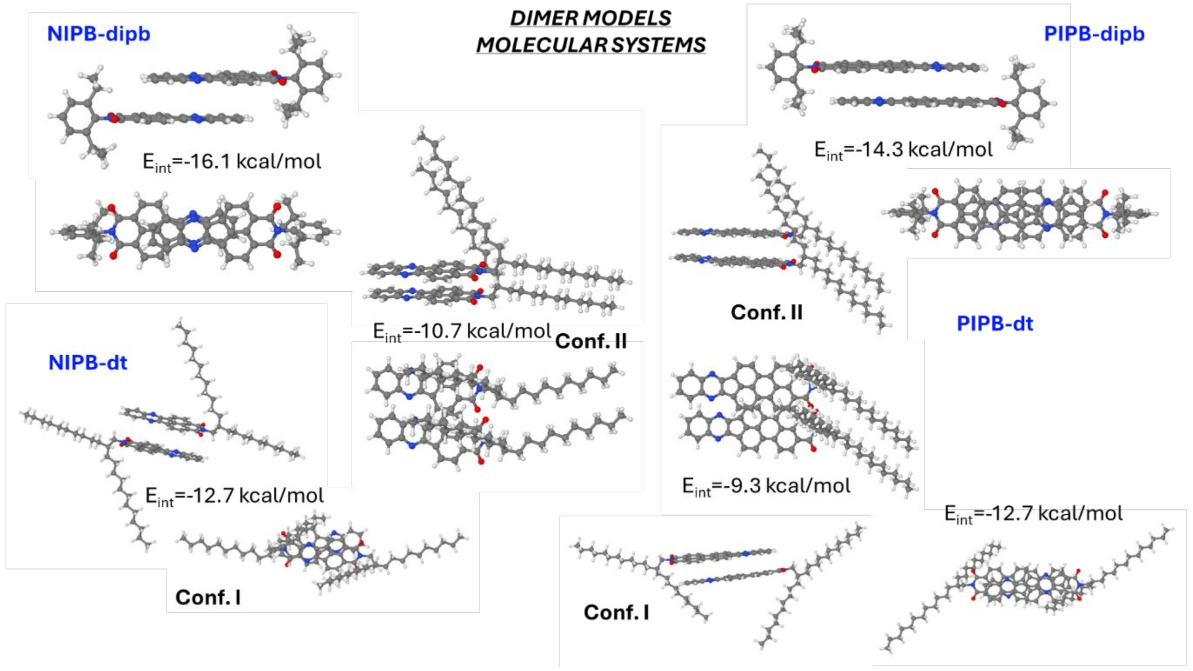


**Figure S29.** All-electron B3LYP/6-311G** dimeric molecular models for NIPB and PIPB derivatives.

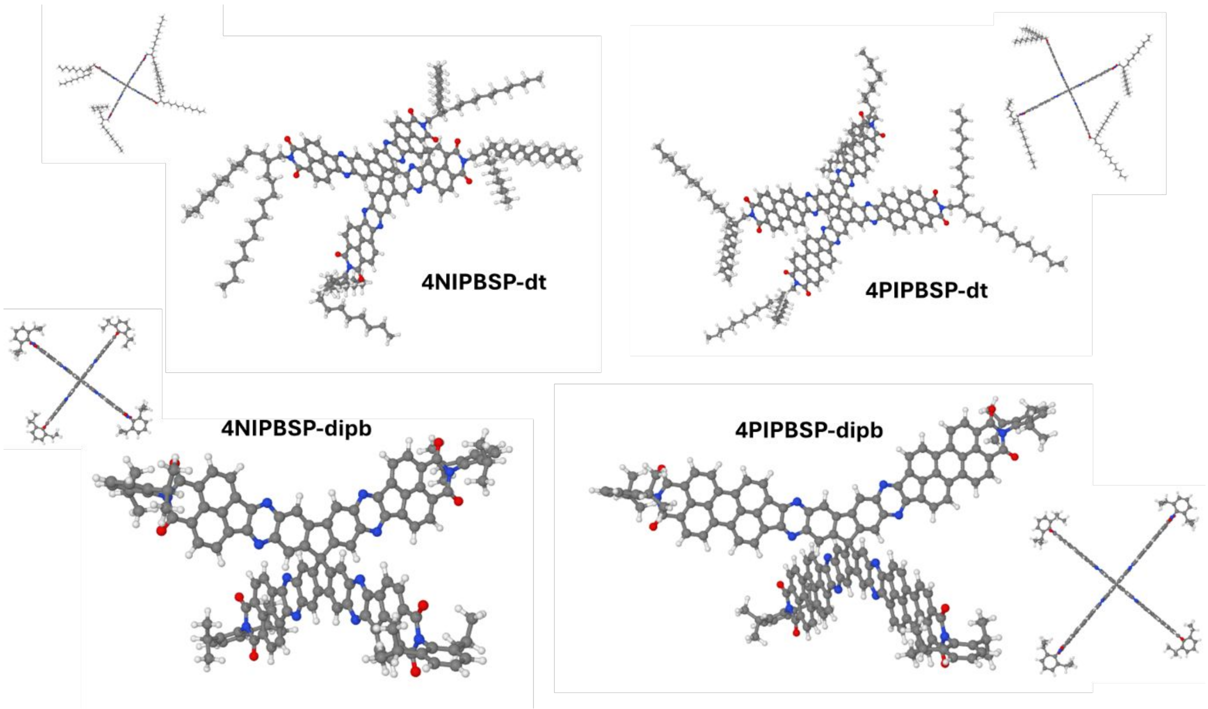


**Figure S30.** All-electron B3LYP/6-311G** optimized structures for 4NIPBSP and 4PIPBSP derivatives.

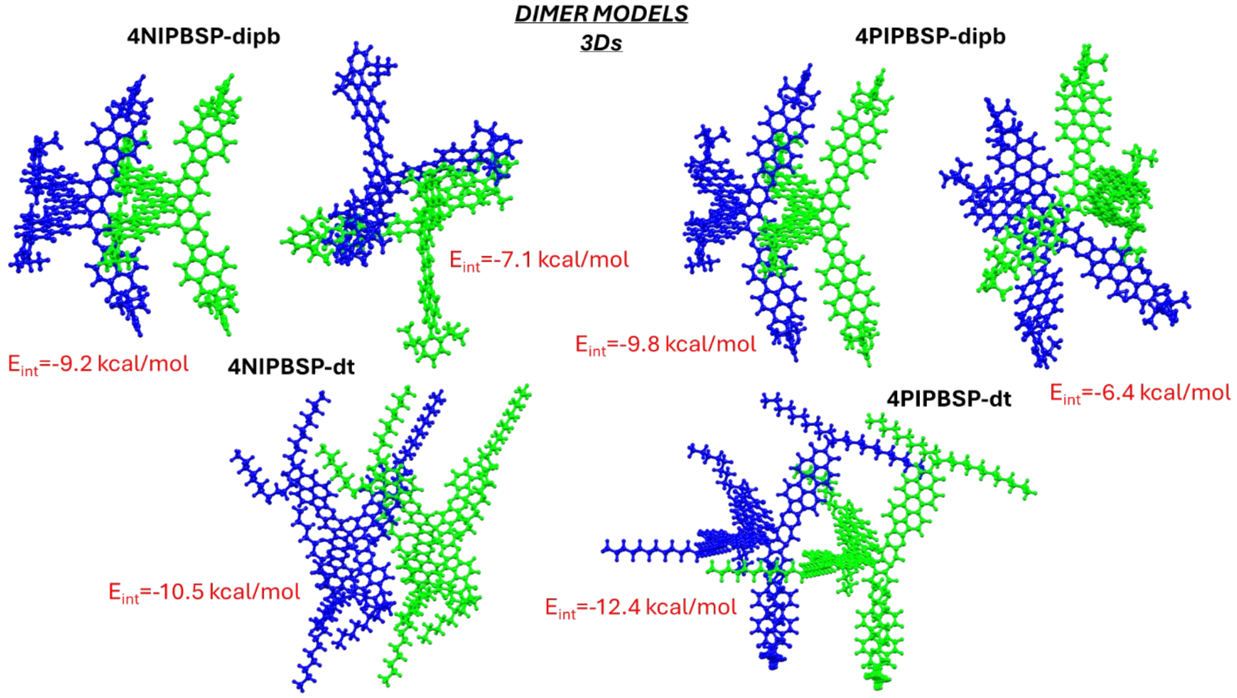


**Figure S31.** All-electron B3LYP/6-311G** dimeric molecular models for 4NIPBSP and 4PIPBSP derivatives.

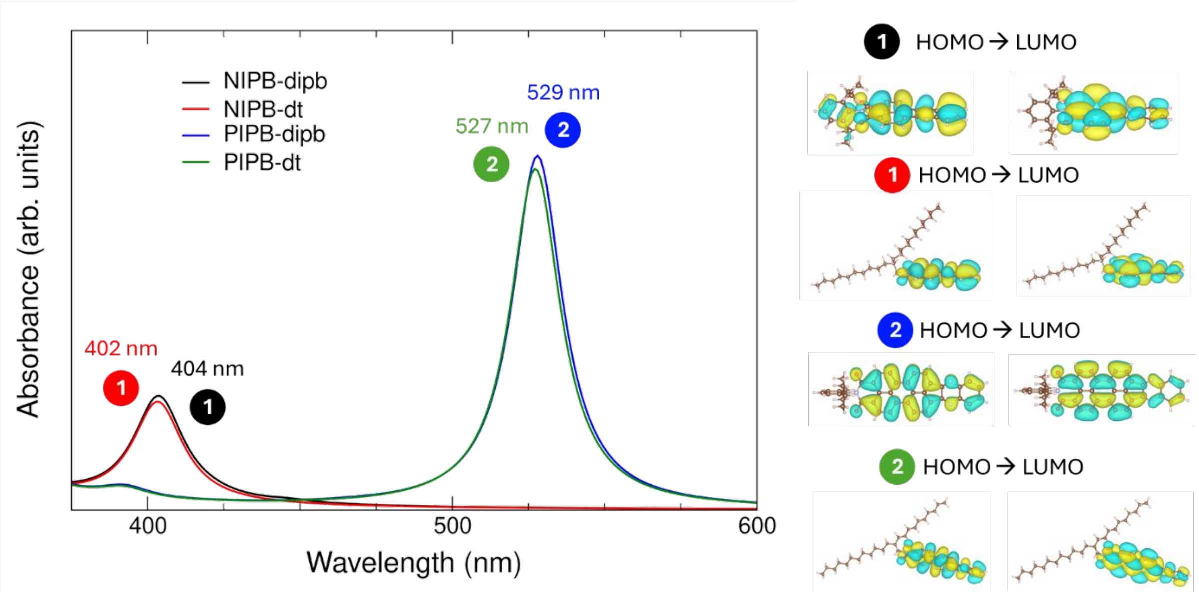


**Figure S32.** Theoretical photoabsorption spectroscopy using CAM-B3LYP/6-311G** for 1) **NIPB-dipb** (black) and **NIPB-dt** (red) and 2) **PIPB-dipb** (blue) and **PIPB-dt** (green).

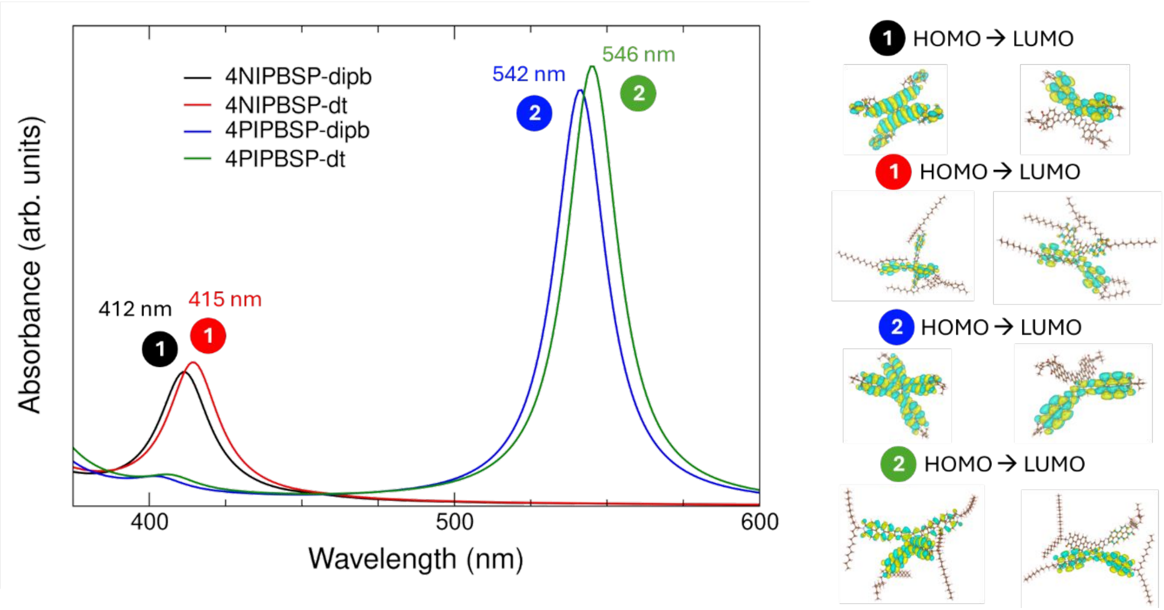


**Figure S33.** Theoretical photoabsorption spectroscopy using CAM-B3LYP/6-311G** for 1) **4NIPBSP-dipb** (black) and **4NIPBSP-dt** (red) and 2) **4PIPBSP-dipb** (blue) and **4PIPBSP-dt** (green).

| | NIP-dipb | NIP-dt | PIPB-dipb | PIPB-dt | 4NIPBSP-dipb | 4NIPBSP-dt | 4PIPBSP-dipb | 4PIPBSP-dt |
|---|---|---|---|---|---|---|---|---|
| $\lambda_e$ (eV) | 0.34 | 0.34 | 0.27 | 0.26 | 0.27 | 0.28 | 0.24 | 0.24 |
| $\lambda_h$ (eV) | 0.13 | 0.11 | 0.11 | 0.13 | 0.11 | 0.10 | 0.09 | 0.10 |

**Table S1.** Calculated reorganization energies for all rylenimide-based semiconductors.

## 4. UV-Vis and Electrochemical data

| | [C] (mol/L) | $\lambda_{abs}^{max,sol}$ (nm) | $\varepsilon_\lambda^{max,\ sol}$ ($M^{-1}\cdot cm^{-1}$) | $\lambda_{abs}^{onset}$ (nm) | $\lambda_{abs}^{max,film}$ (nm) | $\lambda_{film}^{onset}$ (nm) | $\lambda_{em}^{max,sol}$ (nm) | $E_g^{opt}$ (eV) |
|---|---|---|---|---|---|---|---|---|
| **NIPB-dipb** | $10^{-5}$ | 346 | 66844 | 435 | 341 | 469 | 532 | 2.85 |
| **NIPB-dt** | $10^{-5}$ | 346 | 63320 | 434 | 344 | 456 | 498 | 2.85 |
| **PIPB-dipb** | $10^{-5}$ | 535 | 55264 | 561 | 568 | 660 | 549 | 2.21 |
| **PIPB-dt** | $10^{-5}$ | 534 | 65704 | 560 | 585 | 671 | 548 | 2.21 |
| **4NIPBSP-dipb** | $10^{-5}$ | 392 | 94145 | 469 | 408 | 491 | 529 | 2.64 |
| **4NIPBSP-dt** | $10^{-5}$ | 391 | 166311 | 468 | 392 | 514 | 530 | 2.64 |
| **4PIPBSP-dipb** | $10^{-5}$ | 548 | 131335 | 591 | 561 | 624 | 601 | 2.10 |
| **4PIPBSP-dt** | $10^{-5}$ | 554 | 114139 | 605 | 525 | 620 | 626 | 2.05 |

**Table S2.** Physicochemical properties obtained from the UV-vis absorption measurements in chloroform solutions and thin films for **NIPB-dipb**, **NIPB-dt**, **4NIPBSP-dipb**, **4NIPBSP-dt**, **PIPB-dipb**, **PIPB-dt**, **4PIPBSP-dipb** and **4PIPBSP-dt.** The Energy band gap derived from the low-energy absorption edge using the equation $E_g^{opt} = 1240/ \lambda^{onset}$.

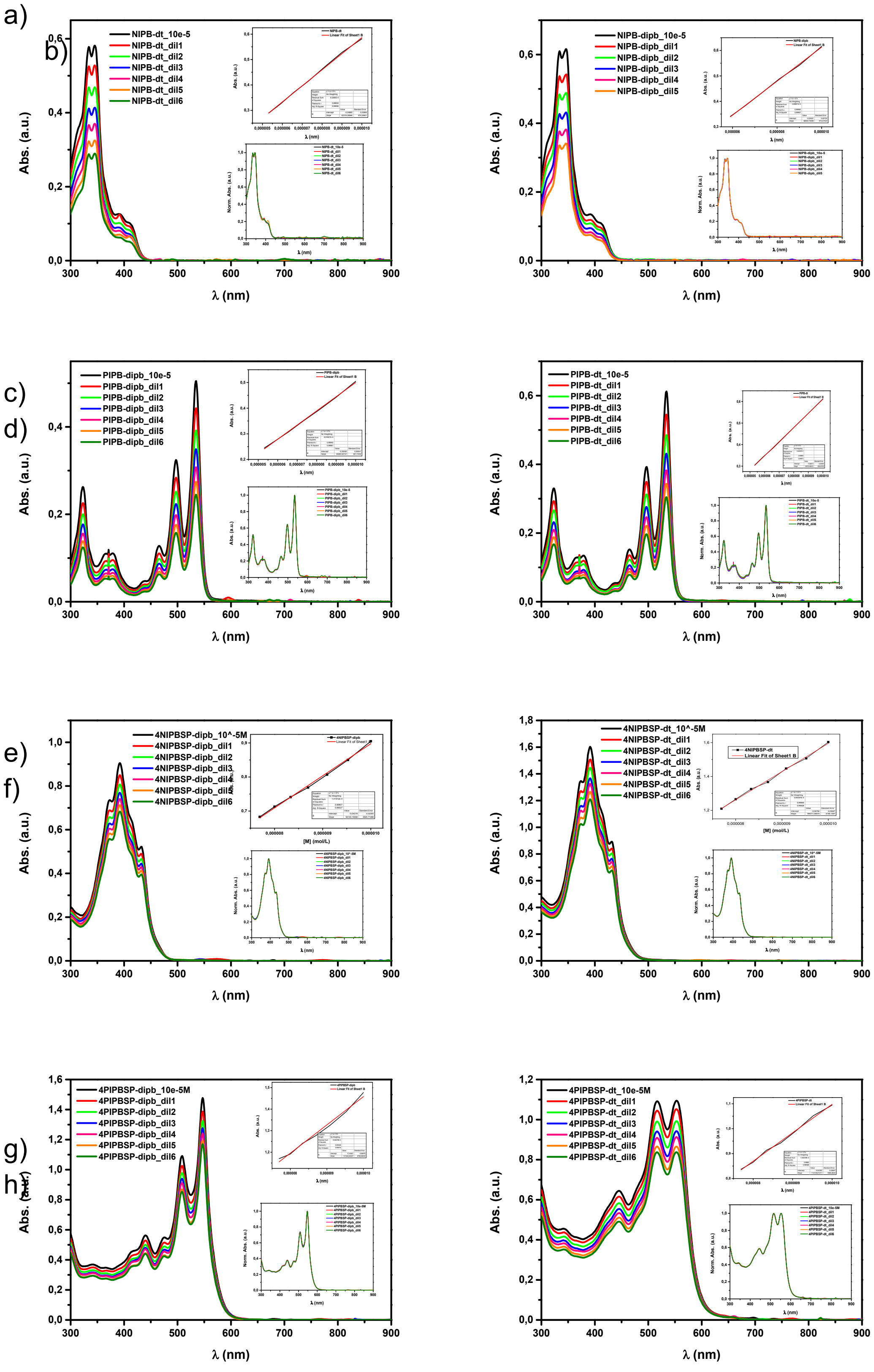


**Figure S34.** concentration-dependent experiments carried out for a) **NIPB-dipb**, b) **NIPB-dt**, c) **PIPB-dipb**, d) **PIPB-dt**, e)**4NIPBSP-dipb**, f)**4NIPBSP-dt**, g)**4PIPBSP-dipb** and h) **4PIPBSP-dt** at the same $1.0\cdot10^{-5}$M-$5.3\cdot10^{-6}$M concentration range for each semiconductor.

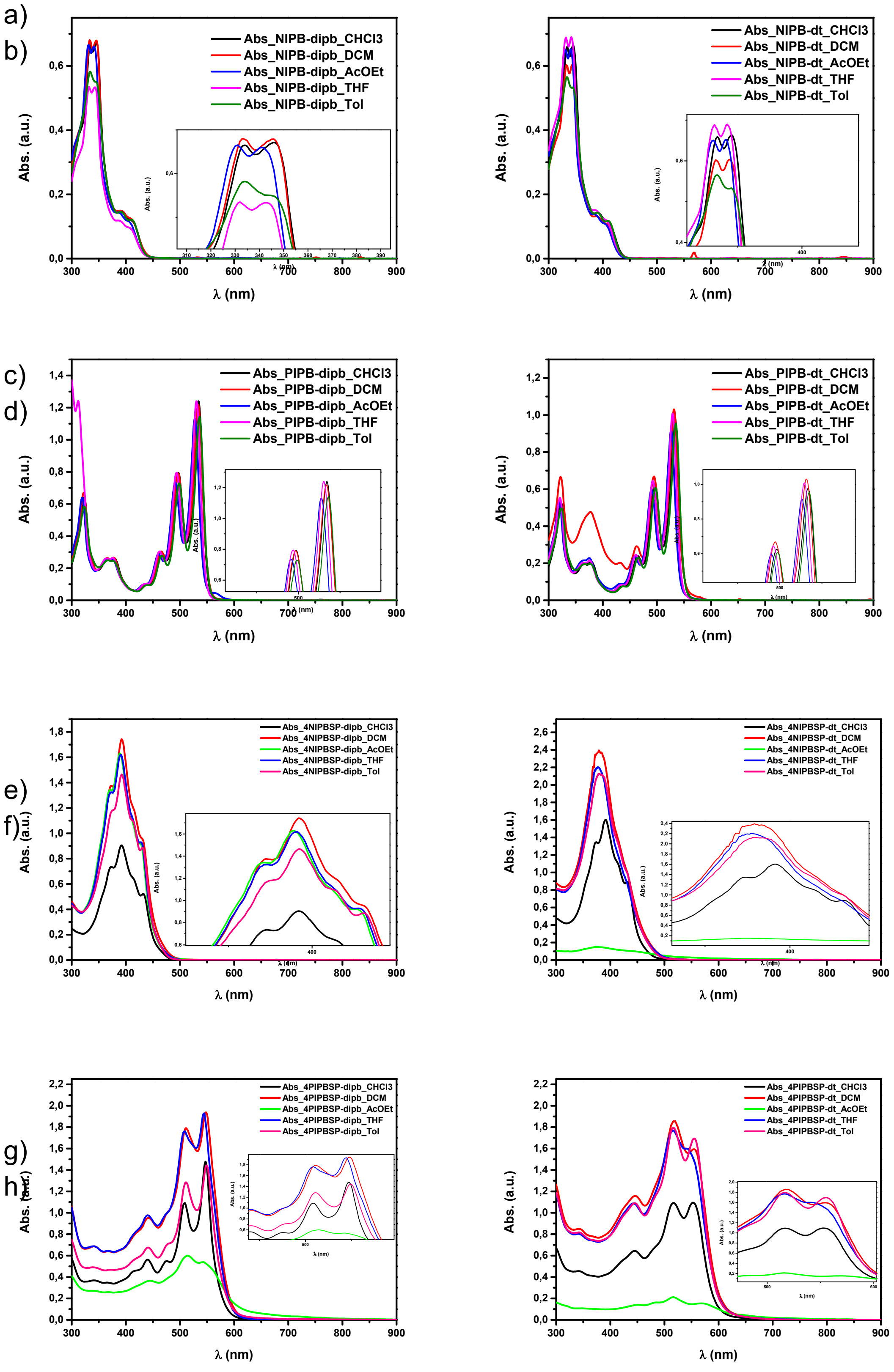


**Figure S35.** Solvent-dependent experiments carried out for a) **NIPB-dipb**, b) **NIPB-dt**, c) **PIPB-dipb**, d) **PIPB-dt**, e) **4NIPBSP-dipb**, f) **4NIPBSP-dt**, g) **4PIPBSP-dipb** and h) **4PIPBSP-dt** at $10^{-5}$M concentration for each semiconductor.

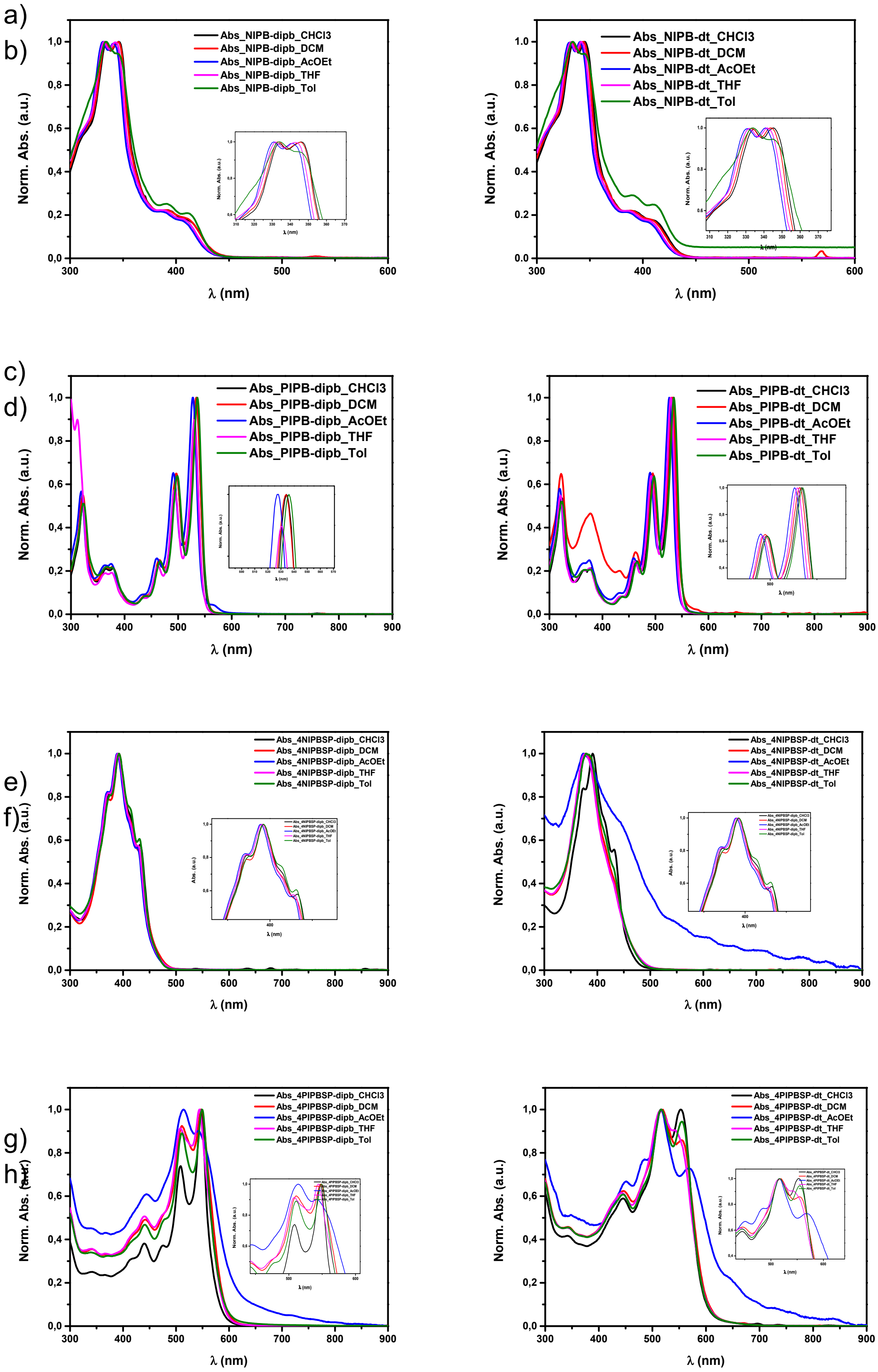


**Figure S36.** Normalized Solvent-dependent normalized experiments carried out for a) **NIPB-dipb**, b) **NIPB-dt**, c) **PIPB-dipb**, d) **PIPB-dt**, e) **4NIPBSP-dipb**, f) **4NIPBSP-dt**, g) **4PIPBSP-dipb** and h) **4PIPBSP-dt** at $10^{-5}$M concentration for each semiconductor.

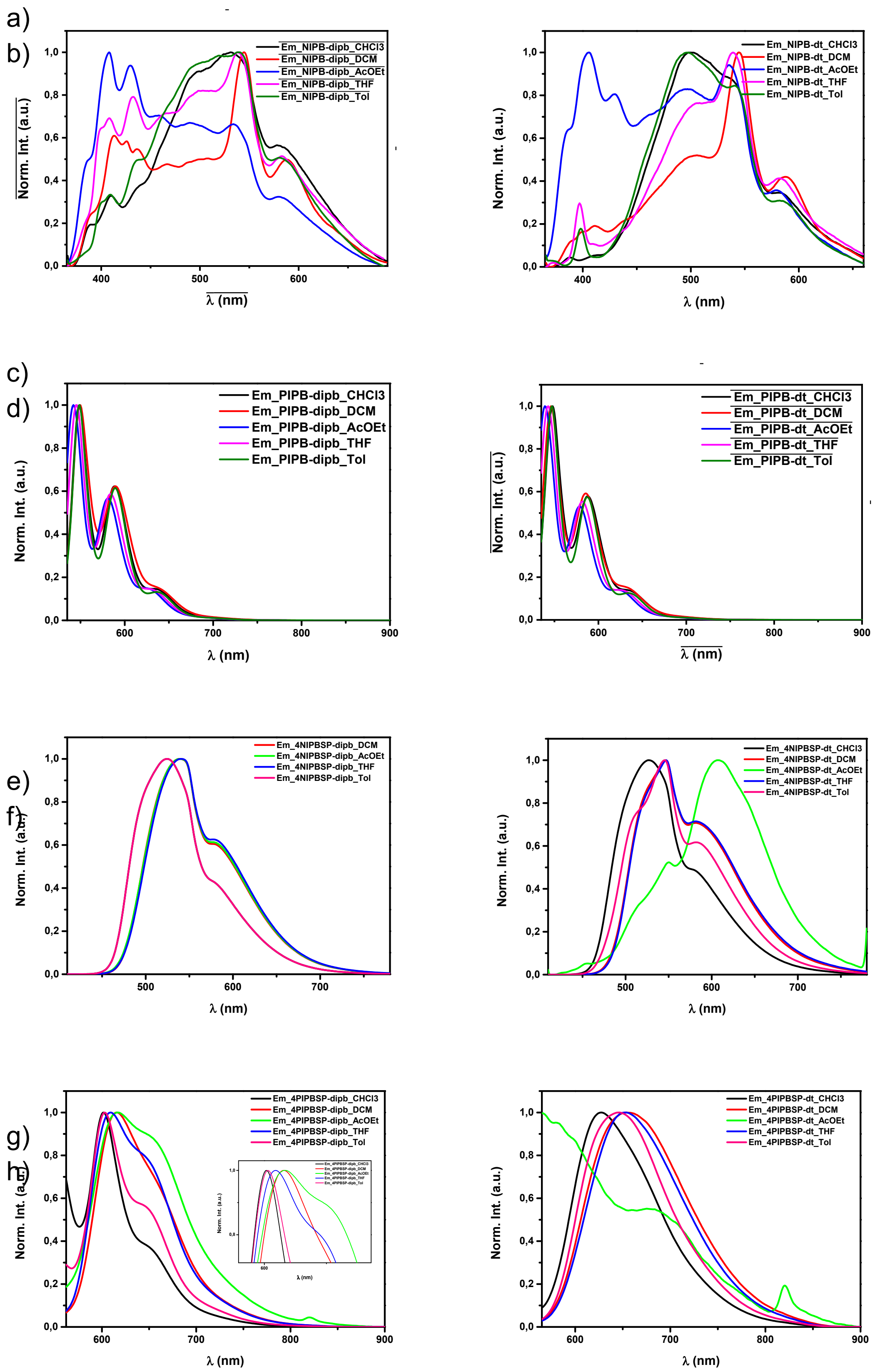


**Figure S37.** Emission experiments carried out for a) **NIPB-dipb**, b) **NIPB-dt**, c) **PIPB-dipb**, d) **PIPB-dt**, e)**4NIPBSP-dipb**, f)**4NIPBSP-dt**, g)**4PIPBSP-dipb** and h) **4PIPBSP-dt** at $10^{-5}$M concentration for each semiconductor.

| | $E_{redI}$[a] (V) | $E_{oxI}$[a] (V) | $LUMO_{exp}$[b] (eV) | $HOMO_{exp}$[c] (eV) | $LUMO_{theo}$[d] (eV) | $HOMO_{theo}$[d] (eV) |
|---|---|---|---|---|---|---|
| **NIPB-dipb** | -1.31 | 0.72 | -3.79 | -6,64 | -3.30 | -6.54 |
| **NIPB-dt** | -1.34 | 0.82 | -3.77 | -6,62 | -3.28 | -6.72 |
| **PIPB-dipb** | -1.13 | 1.02 | -3.97 | -6,18 | -3.46 | -5.97 |
| **PIPB-dt** | -1.17 | 0.91 | -3.94 | -6,15 | -3.44 | -5.95 |
| **4NIPBSP-dipb** | -1.20 | - | -3.91 | -6,55 | -3.50 | -6.60 |
| **4NIPBSP-dt** | -1.21 | - | -3.89 | -6,53 | -3.48 | -6.57 |
| **4PIPBSP-dipb** | -1.14 | 1.01 | -3.96 | -6,06 | -3.57 | -6.00 |
| **4PIPBSP-dt** | -1.19 | - | -3.92 | -5,97 | -3.56 | -5.99 |

**Table S3.** a) Electrochemical data obtained from cyclic voltammetry experiments carried out in DCM/$TBAPF_6$ (0.1 M) solutions at a scan rate of 0.1 V/s, using Pt as working and the counter electrodes, and the $Fc/Fc^+$ redox couple as internal reference. b) Estimated from ELUMO = – 5.1 eV – $E_{redI}$. c) Estimated from $E_{HOMO} = E_g^{opt} + E_{LUMO}$, d) obtained from density functional theory-based experiments using B3LYP/6-311G** set.

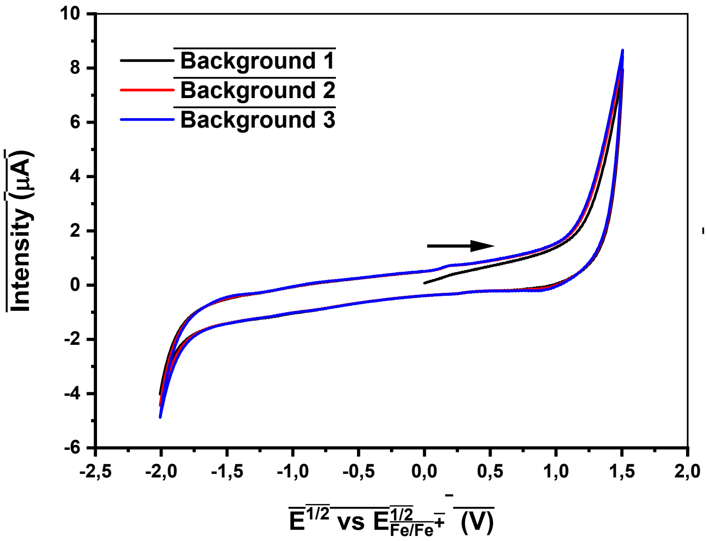


**Figure S38.** Cyclic voltammetry of Pt working electrode (background) and 0.1M TBAHFP in dichloromethane solution.

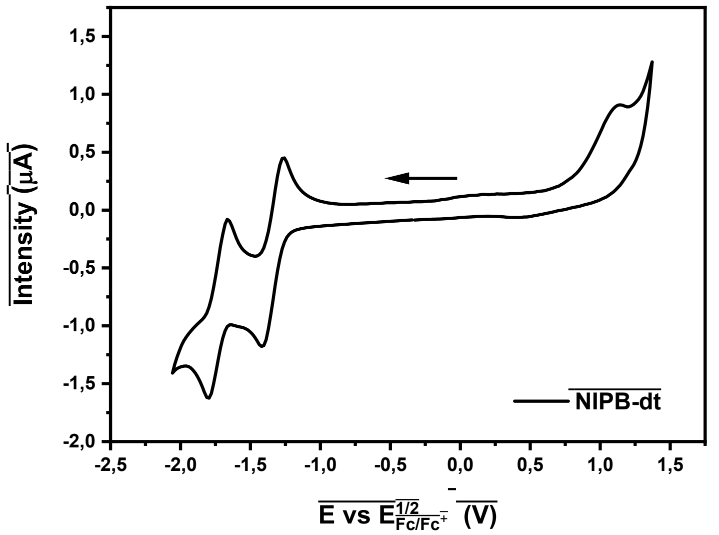


**Figure S39.** Cyclic voltammetry of **NIPB-dt** and TBAHFP 0.1M in dichloromethane solution.

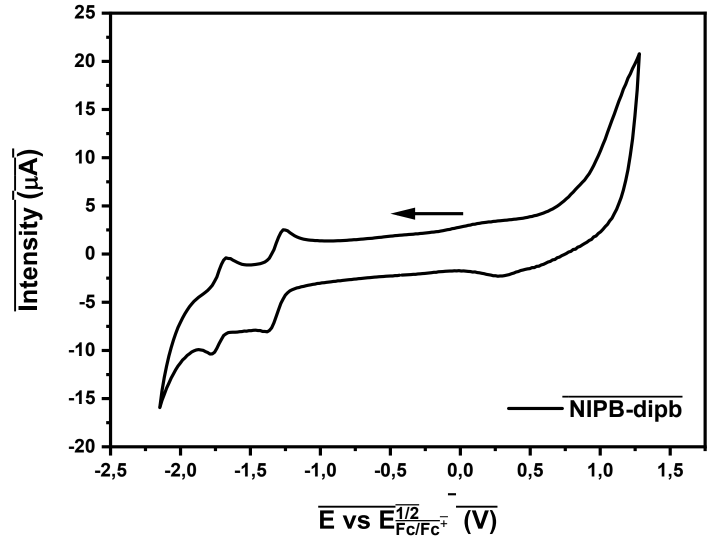


**Figure S40.** Cyclic voltammetry of **NIPB-dipb** and TBAHFP 0.1M in dichloromethane solution.

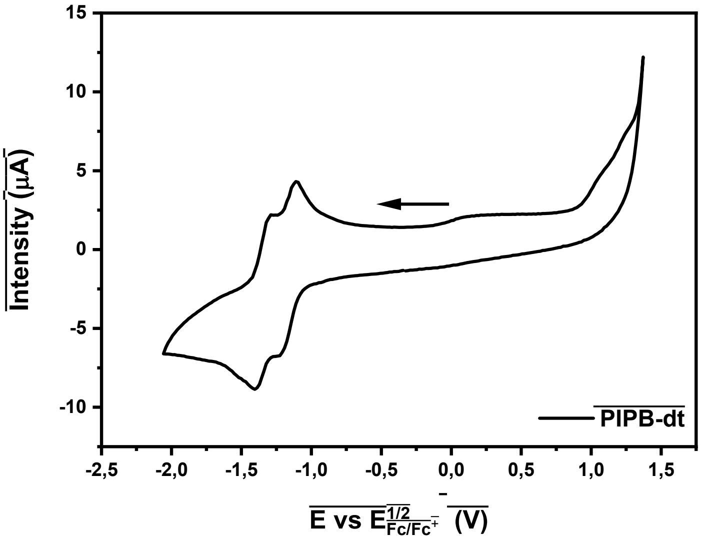


**Figure S41.** Cyclic voltammetry of **PIPB-dt** and TBAHFP 0.1M in dichloromethane solution.

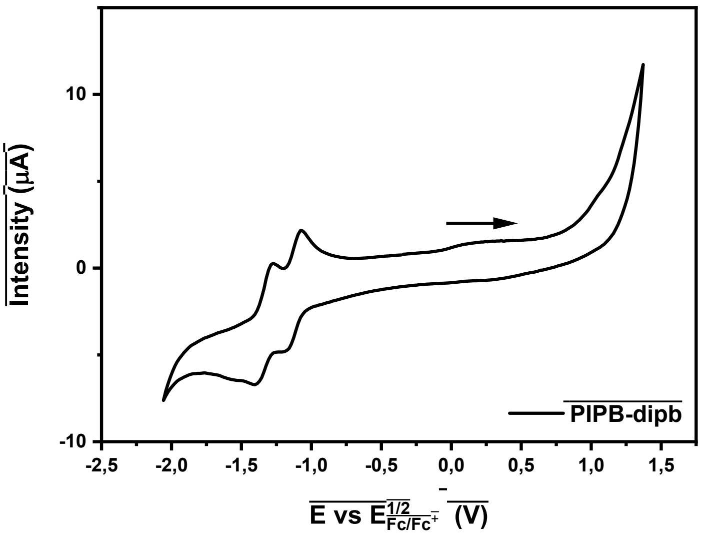


**Figure S42.** Cyclic voltammetry of **PIPB-dipb** and TBAHFP 0.1M in dichloromethane solution.

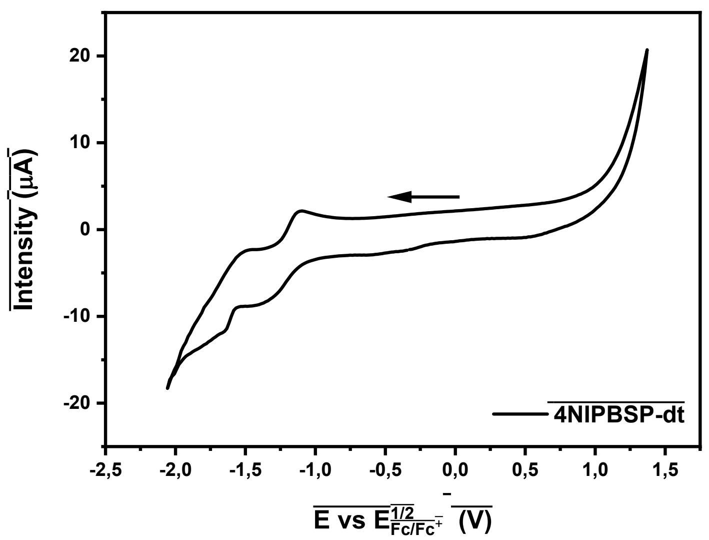


**Figure S43.** Cyclic voltammetry of **4NIPBSP-dt** and TBAHFP 0.1M in dichloromethane solution.

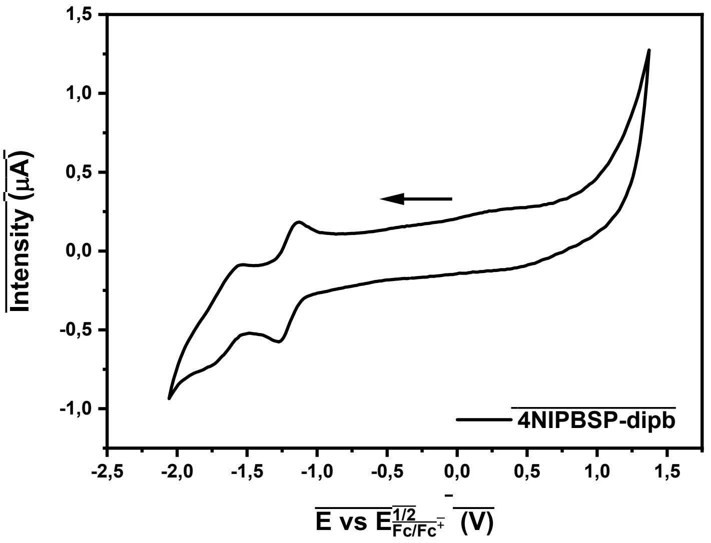


**Figure S44.** Cyclic voltammetry of **4NIPBSP-dipb** and TBAHFP 0.1M in dichloromethane solution.

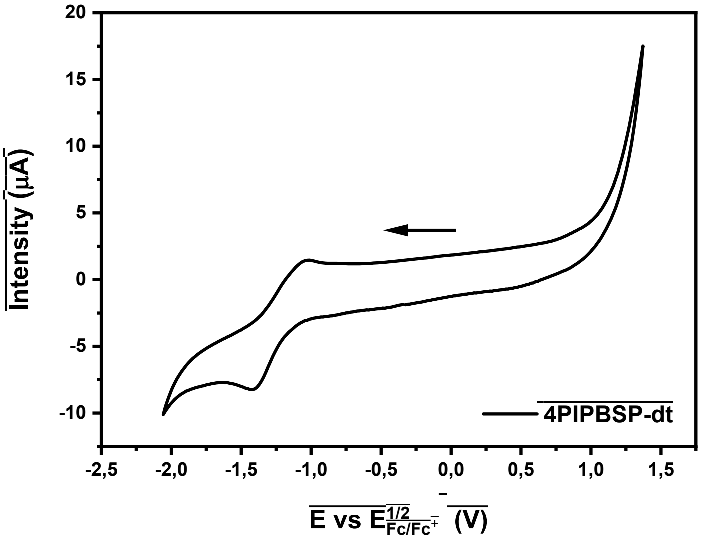


**Figure S45.** Cyclic voltammetry of **4PIPBSP-dt** and TBAHFP 0.1M in dichloromethane solution.

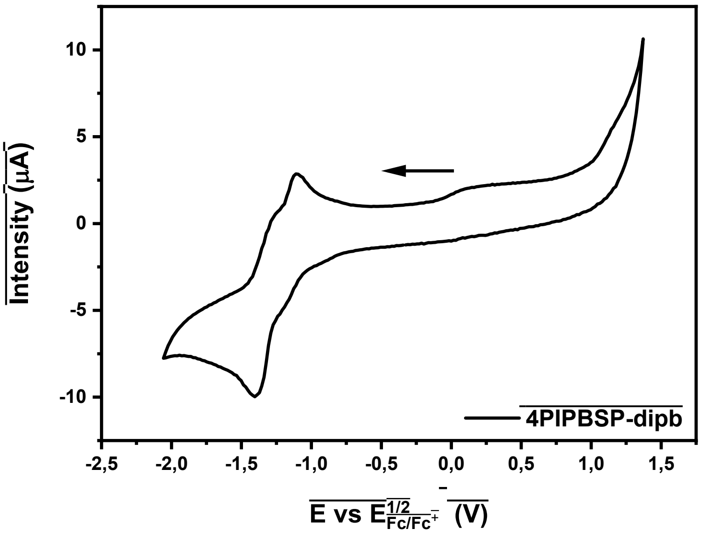


**Figure S46.** Cyclic voltammetry of **4PIPBSP-dipb** and TBAHFP 0.1M in dichloromethane solution.

## XYZ cartesian coordinates (in Å) of the fully optimized NIP-dipb, NIP-dt, PIPB-dipb, PIPB-dt, 4NIPBSP-dipb, 4NIPBSP-dt, 4PIPBSP-dipb and 4PIPBSP-dt molecular systems, and the resulting total energy (in atomic units)

### NIPB-dipb (E=-1549.60765212 A.U.)

```
C      8.53188     0.23540     0.69482
C      8.53830    -0.38443    -0.57433
```

| | | | |
|---|---|---|---|
| C | 7.34275 | 0.55533 | 1.31060 |
| C | 6.11297 | 0.26699 | 0.67876 |
| C | 6.11947 | -0.36254 | -0.60884 |
| C | 7.35551 | -0.67813 | -1.21492 |
| N | 4.94345 | 0.59788 | 1.31617 |
| C | 3.85436 | 0.29821 | 0.66637 |
| C | 3.86091 | -0.34377 | -0.64365 |
| N | 4.95646 | -0.66802 | -1.27042 |
| C | 2.44966 | 0.51263 | 1.05910 |
| C | 1.68221 | 0.00224 | -0.01174 |
| C | 2.46021 | -0.52929 | -1.06454 |
| C | 0.29157 | 0.01563 | -0.02520 |
| C | -0.36219 | -0.53491 | -1.14773 |
| C | 0.39374 | -1.06361 | -2.18704 |
| C | 1.81165 | -1.06586 | -2.16058 |
| C | 1.79033 | 1.04897 | 2.14875 |
| C | 0.37227 | 1.06685 | 2.15127 |
| C | -0.37335 | 0.56583 | 1.09097 |
| C | -1.85661 | 0.58114 | 1.09574 |
| N | -2.48849 | 0.09407 | -0.07535 |
| C | -1.84479 | -0.53597 | -1.17363 |
| O | -2.48867 | -1.03526 | -2.07180 |
| C | -3.94553 | 0.08944 | -0.08633 |
| O | -2.50814 | 0.98888 | 2.03514 |
| C | -4.60243 | -1.04202 | 0.42726 |
| C | -5.99932 | -1.05828 | 0.41174 |
| C | -6.71951 | 0.01041 | -0.09976 |
| C | -6.04664 | 1.11534 | -0.60272 |
| C | -4.64918 | 1.19045 | -0.60896 |
| C | -3.85646 | -2.24410 | 0.99570 |
| C | -4.17881 | -2.45323 | 2.48697 |
| C | -4.12846 | -3.51564 | 0.17024 |
| C | -4.04554 | 2.49316 | -1.14512 |
| C | -3.70001 | 3.47575 | -0.00803 |
| C | -2.88345 | 2.36146 | -2.14493 |
| H | 9.47224 | 0.45957 | 1.18497 |

| | | | |
|---|---|---|---|
| H | 9.48352 | -0.62945 | -1.04476 |
| H | 7.30987 | 1.03079 | 2.28343 |
| H | 7.33248 | -1.15296 | -2.18832 |
| H | -0.13228 | -1.48337 | -3.03607 |
| H | 2.36733 | -1.48580 | -2.99031 |
| H | 2.33771 | 1.45003 | 2.99320 |
| H | -0.16209 | 1.47979 | 2.99846 |
| H | -6.52591 | -1.92121 | 0.80271 |
| H | -7.80354 | -0.01700 | -0.11088 |
| H | -6.61361 | 1.94858 | -1.00402 |
| H | -2.78521 | -2.04656 | 0.92974 |
| H | -3.58296 | -3.27642 | 2.89261 |
| H | -3.95834 | -1.55168 | 3.06276 |
| H | -5.23327 | -2.70140 | 2.63796 |
| H | -3.53254 | -4.35092 | 0.55044 |
| H | -3.87280 | -3.35844 | -0.87974 |
| H | -5.18112 | -3.80831 | 0.22250 |
| H | -4.86462 | 2.95452 | -1.70698 |
| H | -3.38728 | 4.43744 | -0.42673 |
| H | -4.56598 | 3.64913 | 0.63524 |
| H | -2.89525 | 3.10152 | 0.62608 |
| H | -2.75633 | 3.30990 | -2.67491 |
| H | -3.07441 | 1.58253 | -2.88543 |
| H | -1.93400 | 2.14259 | -1.65291 |

**NIPB-dt (E=-2026.34589465 A.U.)**

| | | | |
|---|---|---|---|
| C | 9.37174 | -4.19785 | 1.49300 |
| C | 8.21296 | -3.66146 | 2.00794 |
| C | 7.02139 | -3.67904 | 1.24997 |
| C | 7.03416 | -4.26198 | -0.05921 |
| C | 8.23823 | -4.80485 | -0.55946 |
| N | 5.88211 | -3.13509 | 1.78837 |
| C | 4.82785 | -3.18904 | 1.02439 |
| C | 4.84090 | -3.78207 | -0.30872 |
| N | 5.90801 | -4.30598 | -0.84253 |
| C | 3.46264 | -2.69928 | 1.28931 |
| C | 2.72450 | -3.00925 | 0.12528 |
| C | 3.48401 | -3.66038 | -0.87231 |
| C | 1.37705 | -2.70098 | -0.02526 |
| C | 0.74887 | -3.06110 | -1.23559 |
| C | 1.48546 | -3.70515 | -2.22195 |

| | | | |
|---|---|---|---|
| C | 2.86015 | -4.01189 | -2.05428 |
| C | 2.81650 | -2.06169 | 2.33142 |
| C | 1.44186 | -1.74089 | 2.19256 |
| C | 0.72599 | -2.04717 | 1.04159 |
| C | -0.70918 | -1.70292 | 0.89102 |
| N | -1.30818 | -2.04748 | -0.34117 |
| C | -0.68695 | -2.73402 | -1.40742 |
| O | -1.31408 | -3.02738 | -2.40727 |
| C | -2.74253 | -1.72690 | -0.52027 |
| C | -3.02550 | -0.48756 | -1.39539 |
| O | -1.34201 | -1.14565 | 1.76760 |
| C | 9.38435 | -4.77251 | 0.20286 |
| C | -15.38751 | 0.84864 | 1.96985 |
| C | -14.37260 | 0.67065 | 0.83705 |
| C | -12.92518 | 0.56163 | 1.33012 |
| C | -11.90246 | 0.38464 | 0.20243 |
| C | -10.45445 | 0.27756 | 0.69414 |
| C | -9.43446 | 0.10256 | -0.43658 |
| C | -7.98451 | -0.00146 | 0.05005 |
| C | -6.97002 | -0.17540 | -1.08565 |
| C | -5.51600 | -0.27378 | -0.60780 |
| C | -4.52589 | -0.45733 | -1.76701 |
| C | 8.35677 | 9.79336 | 0.09720 |
| C | 6.91692 | 9.43032 | 0.47152 |
| C | 6.44967 | 8.10113 | -0.13268 |
| C | 5.00933 | 7.72844 | 0.23557 |
| C | 4.54559 | 6.39878 | -0.37036 |
| C | 3.10525 | 6.02256 | -0.00508 |
| C | 2.64734 | 4.69230 | -0.61416 |
| C | 1.20843 | 4.30791 | -0.25190 |
| C | 0.76033 | 2.97612 | -0.86502 |
| C | -0.67440 | 2.57695 | -0.50237 |
| C | -1.10707 | 1.24171 | -1.11901 |
| C | -2.54977 | 0.84755 | -0.77057 |
| H | 10.28309 | -4.18078 | 2.07944 |
| H | 8.17605 | -3.21499 | 2.99425 |
| H | 8.22096 | -5.23917 | -1.55172 |
| H | 0.97973 | -3.97251 | -3.14201 |
| H | 3.40294 | -4.51287 | -2.84675 |
| H | 3.34201 | -1.80515 | 3.24335 |
| H | 0.91916 | -1.23928 | 2.99808 |
| H | -3.19903 | -2.60099 | -0.98541 |
| H | -3.15120 | -1.59713 | 0.47910 |
| H | -2.48714 | -0.63383 | -2.33801 |
| H | 10.30531 | -5.19021 | -0.18722 |
| H | -16.40827 | 0.92329 | 1.58485 |
| H | -15.35614 | 0.00413 | 2.66553 |
| H | -15.18239 | 1.75694 | 2.54515 |
| H | -14.45297 | 1.51331 | 0.13958 |
| H | -14.62462 | -0.22644 | 0.25851 |
| H | -12.84514 | -0.28194 | 2.02824 |
| H | -12.67357 | 1.45911 | 1.91023 |
| H | -11.98497 | 1.22784 | -0.49564 |
| H | -12.15473 | -0.51322 | -0.37657 |
| H | -10.37071 | -0.56627 | 1.39119 |
| H | -10.20196 | 1.17505 | 1.27350 |
| H | -9.52089 | 0.94550 | -1.13448 |
| H | -9.68665 | -0.79579 | -1.01481 |
| H | -7.89641 | -0.84470 | 0.74725 |
| H | -7.73114 | 0.89684 | 0.62770 |
| H | -7.06251 | 0.66598 | -1.78469 |
| H | -7.22286 | -1.07526 | -1.66131 |
| H | -5.43137 | -1.10914 | 0.09722 |
| H | -5.26268 | 0.62968 | -0.04117 |
| H | -4.67311 | 0.35679 | -2.48710 |
| H | -4.77234 | -1.38169 | -2.30421 |

| | | | |
|---|---|---|---|
| H | 8.65829 | 10.74531 | 0.54285 |
| H | 9.05983 | 9.02825 | 0.44139 |
| H | 8.47258 | 9.88297 | -0.98753 |
| H | 6.24204 | 10.23116 | 0.14572 |
| H | 6.82500 | 9.38204 | 1.56341 |
| H | 7.12592 | 7.30006 | 0.19342 |
| H | 6.54273 | 8.14937 | -1.22553 |
| H | 4.33362 | 8.52966 | -0.09072 |
| H | 4.91679 | 7.68030 | 1.32834 |
| H | 5.22187 | 5.59813 | -0.04384 |
| H | 4.63949 | 6.44733 | -1.46298 |
| H | 2.42802 | 6.82230 | -0.33174 |
| H | 3.01054 | 5.97260 | 1.08740 |
| H | 3.32677 | 3.89391 | -0.28855 |
| H | 2.74259 | 4.74354 | -1.70656 |
| H | 0.52715 | 5.10440 | -0.57807 |
| H | 1.11200 | 4.25469 | 0.84022 |
| H | 1.44704 | 2.18273 | -0.54249 |
| H | 0.85442 | 3.03191 | -1.95735 |
| H | -1.36463 | 3.36690 | -0.82616 |
| H | -0.76973 | 2.51738 | 0.58912 |
| H | -0.41065 | 0.46172 | -0.79504 |
| H | -1.00799 | 1.29813 | -2.21095 |
| H | -3.20795 | 1.65060 | -1.12442 |
| H | -2.66560 | 0.81437 | 0.31742 |

**PIPB-dipb (E=-1933.21310493 A.U.)**

| | | | |
|---|---|---|---|
| C | -10.93256 | -0.70279 | 0.07138 |
| C | -10.93256 | 0.70279 | -0.07137 |
| C | 1.59965 | 2.40773 | -0.24157 |
| C | 0.20391 | 2.42113 | -0.24154 |
| C | -0.54388 | 1.25181 | -0.12459 |
| C | 0.14676 | 0.00000 | 0.00000 |
| C | 1.57593 | 0.00000 | 0.00000 |
| C | 2.28896 | 1.21599 | -0.12256 |
| C | -0.54388 | -1.25181 | 0.12458 |
| C | 0.20391 | -2.42114 | 0.24152 |
| C | 1.59965 | -2.40773 | 0.24156 |
| C | 2.28896 | -1.21599 | 0.12255 |
| C | -2.01115 | 1.25562 | -0.12473 |
| C | -2.67359 | 0.00000 | -0.00001 |
| C | -2.01115 | -1.25562 | 0.12472 |
| C | -4.07010 | 0.00000 | -0.00001 |
| C | -4.85489 | -1.17064 | 0.11681 |
| C | -4.20891 | -2.38389 | 0.23709 |
| C | -2.79820 | -2.40733 | 0.23902 |
| C | -2.79820 | 2.40733 | -0.23902 |
| C | -4.20891 | 2.38389 | -0.23709 |
| C | -4.85489 | 1.17064 | -0.11682 |
| C | -6.25368 | 0.72500 | -0.07278 |
| C | -6.25368 | -0.72500 | 0.07278 |
| C | 3.77199 | -1.24045 | 0.12926 |
| N | 4.42386 | 0.00000 | 0.00000 |
| C | 3.77199 | 1.24045 | -0.12926 |
| O | 4.40717 | 2.27128 | -0.23867 |
| O | 4.40717 | -2.27128 | 0.23867 |
| C | -9.74645 | -1.39777 | 0.14165 |
| C | -8.51235 | -0.71318 | 0.07215 |
| C | -8.51235 | 0.71318 | -0.07214 |
| C | -9.74645 | 1.39777 | -0.14164 |
| N | -7.34708 | 1.43315 | -0.14437 |
| N | -7.34708 | -1.43315 | 0.14437 |

| | | | |
|---|---|---|---|
| C | 5.87834 | 0.00000 | 0.00000 |
| C | 6.55450 | 0.15939 | 1.21925 |
| C | 7.95150 | 0.15612 | 1.19244 |
| C | 8.64574 | 0.00000 | 0.00001 |
| C | 7.95151 | -0.15612 | -1.19243 |
| C | 6.55450 | -0.15939 | -1.21924 |
| C | 5.82654 | 0.33094 | 2.54632 |
| C | 6.12835 | -0.83391 | 3.50731 |
| C | 6.13869 | 1.69504 | 3.18920 |
| C | 5.82655 | -0.33094 | -2.54632 |
| C | 6.12836 | 0.83391 | -3.50729 |
| C | 6.13871 | -1.69504 | -3.18920 |
| H | -11.87554 | -1.23461 | 0.12549 |
| H | -11.87554 | 1.23461 | -0.12547 |
| H | 2.16519 | 3.32634 | -0.33515 |
| H | -0.29732 | 3.37506 | -0.33682 |
| H | -0.29732 | -3.37506 | 0.33680 |
| H | 2.16519 | -3.32634 | 0.33513 |
| H | -4.76533 | -3.30907 | 0.32919 |
| H | -2.31841 | -3.37280 | 0.33481 |
| H | -2.31841 | 3.37280 | -0.33481 |
| H | -4.76533 | 3.30907 | -0.32919 |
| H | -9.71846 | -2.47528 | 0.25071 |
| H | -9.71846 | 2.47528 | -0.25070 |
| H | 8.50205 | 0.27789 | 2.11828 |
| H | 9.73028 | 0.00000 | 0.00001 |
| H | 8.50206 | -0.27789 | -2.11826 |
| H | 4.75299 | 0.31078 | 2.34850 |
| H | 5.54754 | -0.72863 | 4.42866 |
| H | 5.87568 | -1.79238 | 3.04826 |
| H | 7.18660 | -0.86038 | 3.78272 |
| H | 5.55342 | 1.82741 | 4.10422 |
| H | 5.89762 | 2.51154 | 2.50496 |
| H | 7.19602 | 1.77875 | 3.45664 |
| H | 4.75300 | -0.31079 | -2.34850 |
| H | 5.54756 | 0.72863 | -4.42865 |
| H | 5.87569 | 1.79238 | -3.04824 |
| H | 7.18662 | 0.86039 | -3.78270 |
| H | 5.55343 | -1.82741 | -4.10422 |
| H | 5.89763 | -2.51154 | -2.50496 |
| H | 7.19603 | -1.77874 | -3.45663 |

**PIPB-dt (E=-2409.94565033 A.U.)**

| | | | |
|---|---|---|---|
| C | 12.89214 | -3.74572 | -0.28112 |
| C | 12.52224 | -4.44408 | -1.45205 |
| C | 0.14123 | -1.87081 | -1.14175 |
| C | 1.46766 | -2.25279 | -1.34970 |
| C | 2.48859 | -1.87325 | -0.48132 |
| C | 2.16064 | -1.06449 | 0.65800 |
| C | 0.79961 | -0.67945 | 0.85811 |
| C | -0.20026 | -1.09151 | -0.05313 |
| C | 3.14705 | -0.63062 | 1.60562 |
| C | 2.74075 | 0.15047 | 2.68519 |
| C | 1.40736 | 0.52131 | 2.86808 |
| C | 0.43799 | 0.11743 | 1.96973 |
| C | 3.88514 | -2.27167 | -0.68928 |
| C | 4.84641 | -1.82788 | 0.26488 |
| C | 4.54556 | -1.02563 | 1.40398 |
| C | 6.17635 | -2.20721 | 0.07085 |
| C | 7.23161 | -1.84059 | 0.93803 |
| C | 6.93544 | -1.06337 | 2.03893 |
| C | 5.59830 | -0.66839 | 2.25443 |

| | | | |
|---|---|---|---|
| C | 4.33179 | -3.05685 | -1.75855 |
| C | 5.68123 | -3.42879 | -1.93457 |
| C | 6.61554 | -3.00240 | -1.01315 |
| C | 8.06426 | -3.16421 | -0.83434 |
| C | 8.44590 | -2.44419 | 0.37436 |
| C | -0.96921 | 0.53054 | 2.19364 |
| N | -1.91141 | 0.10885 | 1.24461 |
| C | -1.61796 | -0.70750 | 0.14355 |
| O | -2.50157 | -1.07511 | -0.61080 |
| O | -1.29711 | 1.20859 | 3.15180 |
| C | -3.33269 | 0.46018 | 1.45467 |
| C | -3.86067 | 1.61798 | 0.58337 |
| C | 11.94648 | -3.07228 | 0.45868 |
| C | 10.59221 | -3.07139 | 0.05604 |
| C | 10.21684 | -3.77990 | -1.13251 |
| C | 11.21082 | -4.46107 | -1.87024 |
| N | 8.91841 | -3.81656 | -1.57378 |
| N | 9.67270 | -2.39292 | 0.81507 |
| C | -18.01677 | -4.19293 | -1.32060 |
| C | -17.18494 | -2.95999 | -0.95566 |
| C | -15.67453 | -3.22192 | -0.97017 |
| C | -14.83190 | -1.99431 | -0.60614 |
| C | -13.32216 | -2.25972 | -0.62343 |
| C | -12.47574 | -1.03477 | -0.25849 |
| C | -10.96708 | -1.30592 | -0.27883 |
| C | -10.11414 | -0.08620 | 0.08845 |
| C | -8.60717 | -0.36636 | 0.06426 |
| C | -7.74555 | 0.84552 | 0.43676 |
| C | -6.24000 | 0.55448 | 0.40051 |
| C | -5.38402 | 1.76490 | 0.79887 |
| C | 5.75723 | 9.71493 | -2.13612 |
| C | 4.53584 | 9.42577 | -1.25876 |
| C | 3.82974 | 8.11271 | -1.61553 |
| C | 2.60555 | 7.81474 | -0.74262 |
| C | 1.90492 | 6.49744 | -1.09436 |
| C | 0.68101 | 6.19957 | -0.22068 |
| C | -0.00976 | 4.87416 | -0.56114 |
| C | -1.23237 | 4.57577 | 0.31420 |
| C | -1.90601 | 3.23710 | -0.00814 |
| C | -3.14365 | 2.96299 | 0.85899 |
| H | 13.92917 | -3.74213 | 0.03431 |
| H | 13.27921 | -4.96934 | -2.02304 |
| H | -0.64032 | -2.17689 | -1.82575 |
| H | 1.69361 | -2.86225 | -2.21444 |
| H | 3.46838 | 0.48707 | 3.41149 |
| H | 1.10953 | 1.12937 | 3.71299 |
| H | 7.70852 | -0.75626 | 2.73326 |
| H | 5.39548 | -0.05963 | 3.12607 |
| H | 3.62065 | -3.40517 | -2.49630 |
| H | 5.96785 | -4.03976 | -2.78229 |
| H | -3.89899 | -0.44461 | 1.24328 |
| H | -3.43436 | 0.71206 | 2.50955 |
| H | -3.70323 | 1.34216 | -0.46573 |
| H | 12.20341 | -2.52932 | 1.36029 |
| H | 10.90039 | -4.98878 | -2.76403 |
| H | -19.08786 | -3.97344 | -1.30215 |
| H | -17.76890 | -4.55363 | -2.32383 |
| H | -17.83402 | -5.01440 | -0.62072 |
| H | -17.47907 | -2.60178 | 0.03840 |
| H | -17.41611 | -2.14479 | -1.65210 |
| H | -15.38068 | -3.58069 | -1.96524 |
| H | -15.44363 | -4.03872 | -0.27389 |
| H | -15.12518 | -1.63667 | 0.38936 |
| H | -15.06390 | -1.17762 | -1.30195 |
| H | -13.02966 | -2.61697 | -1.61928 |
| H | -13.09110 | -3.07760 | 0.07127 |

| | | | |
|---|---|---|---|
| H | -12.76625 | -0.67803 | 0.73813 |
| H | -12.70616 | -0.21612 | -0.95250 |
| H | -10.67755 | -1.66157 | -1.27610 |
| H | -10.73843 | -2.12685 | 0.41307 |
| H | -10.40093 | 0.26885 | 1.08677 |
| H | -10.34142 | 0.73579 | -0.60263 |
| H | -8.32105 | -0.71856 | -0.93511 |
| H | -8.38289 | -1.19248 | 0.75135 |
| H | -8.02456 | 1.19406 | 1.43965 |
| H | -7.97129 | 1.67468 | -0.24618 |
| H | -5.95677 | 0.22800 | -0.60784 |
| H | -6.03371 | -0.29412 | 1.06203 |
| H | -5.57806 | 2.02330 | 1.84906 |
| H | -5.71592 | 2.62970 | 0.21245 |
| H | 6.23837 | 10.65585 | -1.85525 |
| H | 6.50467 | 8.92023 | -2.04730 |
| H | 5.47760 | 9.78738 | -3.19188 |
| H | 3.82151 | 10.25378 | -1.34306 |
| H | 4.84167 | 9.39564 | -0.20588 |
| H | 4.54526 | 7.28431 | -1.53093 |
| H | 3.52486 | 8.14239 | -2.66979 |
| H | 1.88855 | 8.64116 | -0.83146 |
| H | 2.91060 | 7.79078 | 0.31163 |
| H | 2.62243 | 5.67151 | -1.00440 |
| H | 1.60025 | 6.52006 | -2.14869 |
| H | -0.04128 | 7.02038 | -0.31819 |
| H | 0.98417 | 6.18702 | 0.83424 |
| H | 0.71380 | 4.05464 | -0.46152 |
| H | -0.31258 | 4.88418 | -1.61624 |
| H | -1.96400 | 5.38673 | 0.20313 |
| H | -0.93231 | 4.57963 | 1.36963 |
| H | -1.17099 | 2.43423 | 0.10887 |
| H | -2.20071 | 3.22318 | -1.06551 |
| H | -3.85701 | 3.77742 | 0.68545 |
| H | -2.86876 | 3.01984 | 1.91748 |

## 4NIPBSP-dipb (E=-6231.72745604 A.U.)

| | | | |
|---|---|---|---|
| C | -0.58024 | 0.45461 | 2.28996 |
| C | -0.94157 | 0.72638 | 0.93968 |
| C | 0.00206 | 0.00298 | -0.02624 |
| C | 0.94493 | -0.71431 | 0.94479 |
| C | 0.58124 | -0.43599 | 2.29311 |
| C | 0.72334 | 0.94195 | -0.99827 |
| C | 0.44451 | 0.57706 | -2.34614 |
| C | -0.44597 | -0.58441 | -2.34180 |
| C | -0.72033 | -0.94247 | -0.99123 |
| C | -1.29062 | 1.00468 | 3.33621 |
| C | -2.39040 | 1.84296 | 3.05905 |
| C | -2.75736 | 2.11006 | 1.69457 |
| C | -2.00738 | 1.53543 | 0.64108 |
| C | 2.01126 | -1.52485 | 0.65205 |
| C | 2.75890 | -2.09496 | 1.70966 |
| C | 2.38916 | -1.82167 | 3.07216 |
| C | 1.28939 | -0.98143 | 3.34328 |
| C | 1.54085 | 2.00338 | -0.70688 |
| C | 2.11514 | 2.74655 | -1.76545 |
| C | 1.83382 | 2.38108 | -3.12746 |
| C | 0.98937 | 1.28427 | -3.39719 |
| C | -0.99367 | -1.29746 | -3.38747 |
| C | -1.83601 | -2.39383 | -3.10938 |
| C | -2.11190 | -2.75297 | -1.74457 |
| C | -1.53536 | -2.00351 | -0.69172 |

| | | | |
|---|---|---|---|
| N | 3.82436 | -2.89856 | 1.39988 |
| C | 4.46610 | -3.39369 | 2.42174 |
| C | 4.08640 | -3.12312 | 3.80423 |
| N | 3.08105 | -2.35989 | 4.12808 |
| N | -2.37496 | -3.09172 | -4.16097 |
| C | -3.14521 | -4.08961 | -3.83085 |
| C | -3.42533 | -4.45342 | -2.44591 |
| N | -2.92607 | -3.80845 | -1.42811 |
| N | 2.93255 | 3.80197 | -1.45715 |
| C | 3.42932 | 4.44100 | -2.47993 |
| C | 3.14296 | 4.07122 | -3.86202 |
| N | 2.36981 | 3.07305 | -4.18441 |
| N | -3.08539 | 2.38473 | 4.11112 |
| C | -4.09105 | 3.14520 | 3.78176 |
| C | -4.46768 | 3.40978 | 2.39728 |
| N | -3.82288 | 2.91132 | 1.37898 |
| C | 5.64100 | -4.28183 | 2.43962 |
| C | 5.91107 | -4.51256 | 3.80739 |
| C | 5.02178 | -3.84893 | 4.68167 |
| C | -3.87527 | -5.02709 | -4.70250 |
| C | -4.55318 | -5.89969 | -3.82262 |
| C | -4.32946 | -5.61621 | -2.45653 |
| C | 4.33621 | 5.60163 | -2.49979 |
| C | 4.55475 | 5.87826 | -3.86813 |
| C | 3.87117 | 5.00308 | -4.74105 |
| C | -5.03013 | 3.87252 | 4.65401 |
| C | -5.91835 | 4.53117 | 3.77490 |
| C | -5.64415 | 4.29594 | 2.40872 |
| C | 6.95673 | -5.31462 | 4.25165 |
| C | 7.12602 | -5.46588 | 5.64409 |
| C | 6.25876 | -4.81445 | 6.51274 |
| C | 5.19662 | -3.99981 | 6.04418 |
| C | 6.45764 | -4.87489 | 1.49513 |
| C | 7.53323 | -5.68896 | 1.93293 |
| C | 7.78668 | -5.91372 | 3.28066 |
| C | -5.36823 | -6.93794 | -4.26045 |
| C | -5.99667 | -7.74002 | -3.28532 |
| C | -5.78090 | -7.47180 | -1.93873 |
| C | -4.94736 | -6.40868 | -1.50728 |
| C | -4.02503 | -5.21176 | -6.06394 |
| C | -4.85269 | -6.26672 | -6.52556 |
| C | -5.51593 | -7.11945 | -5.65119 |
| C | -5.20926 | 4.02767 | 6.01548 |
| C | -6.27468 | 4.84138 | 6.47816 |
| C | -7.14095 | 5.48779 | 5.60479 |
| C | -6.96717 | 5.33230 | 4.21337 |
| C | -7.79587 | 5.92591 | 3.23795 |
| C | -7.53816 | 5.69690 | 1.89174 |
| C | -6.45955 | 4.88367 | 1.45986 |
| C | 4.96076 | 6.39659 | -1.55698 |
| C | 5.79593 | 7.45496 | -1.99699 |
| C | 6.00551 | 7.71741 | -3.34581 |
| C | 5.37043 | 6.91242 | -4.31446 |
| C | 5.51267 | 7.08650 | -5.70659 |
| C | 4.84275 | 6.23226 | -6.57410 |
| C | 4.01511 | 5.18110 | -6.10397 |
| C | 8.91322 | -6.76307 | 3.73885 |
| N | 9.00964 | -6.96844 | 5.14074 |
| C | 8.23666 | -6.31917 | 6.13467 |
| C | -6.39077 | -8.21554 | -6.13461 |
| N | -7.01059 | -9.00956 | -5.13771 |
| C | -6.86893 | -8.85205 | -3.73608 |
| C | 6.88439 | 8.82131 | -3.80541 |
| N | 7.01367 | 8.97812 | -5.20441 |
| C | 6.38358 | 8.18100 | -6.19906 |
| C | -8.25551 | 6.33949 | 6.08923 |

| | | | |
|---|---|---|---|
| N | -9.02734 | 6.98334 | 5.09088 |
| C | -8.92599 | 6.77380 | 3.68995 |
| O | -8.50189 | 6.49216 | 7.26766 |
| O | -9.72290 | 7.27222 | 2.92424 |
| O | 7.46775 | 9.55617 | -3.03395 |
| O | 6.56184 | 8.39701 | -7.37799 |
| O | 8.47894 | -6.46887 | 7.31433 |
| O | -7.43603 | -9.59416 | -2.96170 |
| C | 10.11500 | -7.79945 | 5.60149 |
| C | -7.87438 | -10.09201 | -5.59797 |
| C | -10.13704 | 7.81191 | 5.54557 |
| O | 9.71113 | -7.26609 | 2.97722 |
| O | -6.57901 | -8.43082 | -7.31374 |
| C | -11.37852 | 7.18775 | 5.75568 |
| C | -12.44940 | 7.97683 | 6.18299 |
| C | -12.29431 | 9.33923 | 6.38872 |
| C | -11.05819 | 9.93188 | 6.17037 |
| C | -9.94809 | 9.19183 | 5.74817 |
| C | -9.25616 | -9.85005 | -5.74050 |
| C | -10.05529 | -10.90822 | -6.18847 |
| C | -9.52288 | -12.15478 | -6.47538 |
| C | -8.16203 | -12.36436 | -6.32161 |
| C | -7.30294 | -11.34801 | -5.88767 |
| C | 11.35799 | -7.17902 | 5.81365 |
| C | 12.42468 | -7.97057 | 6.24685 |
| C | 12.26408 | -9.33175 | 6.45636 |
| C | 11.02658 | -9.92071 | 6.23588 |
| C | 9.92050 | -9.17805 | 5.80777 |
| C | 9.23889 | 9.77658 | -5.92822 |
| C | 7.87638 | 10.05050 | -5.69110 |
| C | 7.31424 | 11.31832 | -5.94610 |
| C | 8.15701 | 12.30582 | -6.46918 |
| C | 9.49669 | 12.06143 | -6.72583 |
| C | 10.02459 | 10.81018 | -6.45149 |
| C | 8.60471 | -9.94918 | 5.65669 |
| C | 7.81483 | -9.72370 | 4.35567 |
| C | 7.70173 | -9.79504 | 6.89694 |
| C | 11.58511 | -5.68722 | 5.59611 |
| C | 11.94164 | -4.97688 | 6.91535 |
| C | 12.64192 | -5.42903 | 4.50616 |
| C | -11.59967 | 5.69449 | 5.54189 |
| C | -11.95880 | 4.98687 | 6.86187 |
| C | -12.65131 | 5.42934 | 4.44863 |
| C | -8.63457 | 9.96712 | 5.59880 |
| C | -7.73443 | 9.81927 | 6.84186 |
| C | -7.84043 | 9.74092 | 4.30050 |
| C | -10.01661 | -8.55604 | -5.41358 |
| C | -9.39151 | -7.22217 | -5.85582 |
| C | -10.44925 | -8.50936 | -3.93339 |
| C | -5.81733 | -11.73513 | -5.83108 |
| C | -5.09803 | -11.42831 | -7.16122 |
| C | -4.99211 | -11.25400 | -4.62574 |
| C | 5.87874 | 11.77567 | -5.64747 |
| C | 4.72392 | 10.85704 | -6.08109 |
| C | 5.72423 | 12.21604 | -4.17703 |
| C | 9.99761 | 8.47978 | -5.60927 |
| C | 9.35098 | 7.14295 | -6.00952 |
| C | 10.46965 | 8.45585 | -4.14046 |
| H | -1.04044 | 0.82076 | 4.37417 |
| H | -2.30787 | 1.75801 | -0.37592 |
| H | 2.31350 | -1.75247 | -0.36332 |
| H | 1.03714 | -0.79274 | 4.37989 |
| H | 1.77259 | 2.30378 | 0.30810 |
| H | 0.79785 | 1.03289 | -4.43348 |
| H | -0.80571 | -1.05123 | -4.42565 |
| H | -1.76326 | -2.29909 | 0.32554 |

| | | | |
|---|---|---|---|
| H | 6.41259 | -4.94330 | 7.57737 |
| H | 4.53980 | -3.50823 | 6.75163 |
| H | 6.28932 | -4.72553 | 0.43546 |
| H | 8.18661 | -6.15816 | 1.20722 |
| H | -6.27269 | -8.10347 | -1.20880 |
| H | -4.80502 | -6.22914 | -0.44848 |
| H | -3.52460 | -4.56637 | -6.77570 |
| H | -4.98438 | -6.42513 | -7.58914 |
| H | -4.55345 | 3.53992 | 6.72652 |
| H | -6.43204 | 4.97336 | 7.54190 |
| H | -8.19070 | 6.16187 | 1.16256 |
| H | -6.28810 | 4.73067 | 0.40121 |
| H | 4.82307 | 6.22207 | -0.49673 |
| H | 6.29468 | 8.08688 | -1.27201 |
| H | 4.96845 | 6.38711 | -7.63892 |
| H | 3.50973 | 4.53373 | -6.81041 |
| H | -13.41613 | 7.51692 | 6.35234 |
| H | -13.13519 | 9.94112 | 6.71521 |
| H | -10.94136 | 10.99840 | 6.33036 |
| H | -11.12013 | -10.74035 | -6.30890 |
| H | -10.16519 | -12.95873 | -6.81720 |
| H | -7.74239 | -13.33934 | -6.54495 |
| H | 13.39249 | -7.51354 | 6.41783 |
| H | 13.10177 | -9.93558 | 6.78745 |
| H | 10.90540 | -10.98630 | 6.39886 |
| H | 7.74224 | 13.28605 | -6.67795 |
| H | 10.12541 | 12.84153 | -7.14047 |
| H | 11.07398 | 10.61762 | -6.64673 |
| H | 8.91339 | -10.99967 | 5.63401 |
| H | 7.23912 | -8.79646 | 4.37461 |
| H | 8.47092 | -9.70335 | 3.48369 |
| H | 7.09861 | -10.53985 | 4.22352 |
| H | 8.23989 | -10.06439 | 7.80880 |
| H | 7.34767 | -8.77073 | 7.02042 |
| H | 6.82946 | -10.45012 | 6.80832 |
| H | 10.65217 | -5.24565 | 5.24118 |
| H | 11.17093 | -5.14845 | 7.66990 |
| H | 12.89376 | -5.33504 | 7.31710 |
| H | 12.03401 | -3.89857 | 6.75413 |
| H | 12.73886 | -4.35552 | 4.31800 |
| H | 13.62559 | -5.80286 | 4.80425 |
| H | 12.36317 | -5.91927 | 3.57089 |
| H | -10.66382 | 5.25510 | 5.19195 |
| H | -12.91363 | 5.34295 | 7.25902 |
| H | -12.04692 | 3.90778 | 6.70350 |
| H | -11.19153 | 5.16324 | 7.61881 |
| H | -13.63747 | 5.80040 | 4.74190 |
| H | -12.37077 | 5.91800 | 3.51307 |
| H | -12.74359 | 4.35497 | 4.26310 |
| H | -8.94679 | 11.01648 | 5.57257 |
| H | -8.27608 | 10.08867 | 7.75163 |
| H | -6.86440 | 10.47744 | 6.75408 |
| H | -7.37681 | 8.79660 | 6.96864 |
| H | -7.12670 | 10.55920 | 4.16802 |
| H | -7.26157 | 8.81574 | 4.32353 |
| H | -8.49411 | 9.71586 | 3.42683 |
| H | -10.94017 | -8.64502 | -5.99534 |
| H | -10.16509 | -6.44888 | -5.84819 |
| H | -8.60164 | -6.88286 | -5.18310 |
| H | -8.98514 | -7.28145 | -6.86682 |
| H | -9.59383 | -8.43829 | -3.26097 |
| H | -11.09805 | -7.64479 | -3.76121 |
| H | -11.00345 | -9.41045 | -3.65995 |
| H | -5.83784 | -12.82668 | -5.74448 |
| H | -4.08206 | -11.83491 | -7.13805 |
| H | -5.62606 | -11.88050 | -8.00412 |

| | | | |
|---|---|---|---|
| H | -5.03652 | -10.35692 | -7.35396 |
| H | -5.53690 | -11.37631 | -3.68817 |
| H | -4.07649 | -11.84910 | -4.56374 |
| H | -4.68384 | -10.21065 | -4.71514 |
| H | 5.76321 | 12.68311 | -6.24925 |
| H | 4.57233 | 10.02024 | -5.39678 |
| H | 3.79460 | 11.43396 | -6.08428 |
| H | 4.87889 | 10.46037 | -7.08555 |
| H | 5.85237 | 11.38225 | -3.48606 |
| H | 6.46967 | 12.97101 | -3.91584 |
| H | 4.73109 | 12.64784 | -4.01811 |
| H | 10.90479 | 8.55129 | -6.21829 |
| H | 8.57449 | 6.82448 | -5.31148 |
| H | 8.92181 | 7.18499 | -7.01168 |
| H | 10.11837 | 6.36347 | -6.00326 |
| H | 11.04590 | 9.35286 | -3.90121 |
| H | 9.63138 | 8.41404 | -3.44412 |
| H | 11.10826 | 7.58404 | -3.96675 |

## 4NIPBSP-dt (E=-8138.67275906 A.U.)

| | | | |
|---|---|---|---|
| C | -0.75952 | -0.36616 | 1.43374 |
| C | -0.97491 | -0.94951 | 0.15240 |
| C | -0.23134 | -0.16251 | -0.92971 |
| C | 0.46582 | 0.91927 | -0.09779 |
| C | 0.13152 | 0.78514 | 1.27961 |
| C | -1.15184 | 0.41656 | -2.00964 |
| C | -0.74258 | -0.01893 | -3.30194 |
| C | 0.41912 | -0.89689 | -3.15312 |
| C | 0.73335 | -1.00517 | -1.76832 |
| C | -1.34674 | -0.90115 | 2.56123 |
| C | -2.16260 | -2.04504 | 2.43857 |
| C | -2.36624 | -2.64022 | 1.14571 |
| C | -1.75876 | -2.06450 | 0.00474 |
| C | 1.30637 | 1.91199 | -0.52992 |
| C | 1.84921 | 2.81790 | 0.41151 |
| C | 1.50832 | 2.69027 | 1.80256 |
| C | 0.64136 | 1.65815 | 2.21813 |
| C | -2.22970 | 1.24847 | -1.85035 |
| C | -2.94174 | 1.68553 | -2.99232 |
| C | -2.52473 | 1.25591 | -4.29963 |
| C | -1.41651 | 0.39377 | -4.43236 |
| C | 1.16688 | -1.56274 | -4.10165 |
| C | 2.25439 | -2.36004 | -3.68867 |
| C | 2.56610 | -2.47158 | -2.28944 |
| C | 1.78198 | -1.77650 | -1.33835 |
| N | 2.69588 | 3.79749 | -0.03603 |
| C | 3.16180 | 4.59584 | 0.88389 |
| C | 2.81141 | 4.47121 | 2.29487 |
| N | 2.01028 | 3.54893 | 2.74859 |
| N | 2.99240 | -3.01127 | -4.64530 |
| C | 3.98274 | -3.72733 | -4.19309 |
| C | 4.29712 | -3.84215 | -2.77282 |
| N | 3.61263 | -3.23500 | -1.84337 |
| N | -4.01593 | 2.51742 | -2.81580 |
| C | -4.61977 | 2.88979 | -3.91020 |
| C | -4.19145 | 2.46124 | -5.23769 |
| N | -3.17785 | 1.66639 | -5.43477 |
| N | -2.74130 | -2.56432 | 3.56968 |
| C | -3.47261 | -3.62674 | 3.38375 |
| C | -3.66915 | -4.23718 | 2.07312 |
| N | -3.13692 | -3.76099 | 0.98184 |
| C | 4.08735 | 5.73283 | 0.74456 |

| | | | |
|---|---|---|---|
| C | 4.25231 | 6.23933 | 2.05328 |
| C | 3.51596 | 5.53560 | 3.03214 |
| C | 4.96158 | -4.53659 | -4.94095 |
| C | 5.80916 | -5.09423 | -3.95810 |
| C | 5.46938 | -4.72344 | -2.63750 |
| C | -5.78852 | 3.77165 | -4.07337 |
| C | -6.00972 | 3.83926 | -5.46720 |
| C | -5.08955 | 3.08321 | -6.22670 |
| C | -4.22321 | -4.42248 | 4.37152 |
| C | -4.82377 | -5.46348 | 3.62920 |
| C | -4.53350 | -5.41702 | 2.24704 |
| C | 5.06370 | 7.32874 | 2.34983 |
| C | 5.14544 | 7.74457 | 3.69506 |
| C | 4.42224 | 7.06167 | 4.66543 |
| C | 3.60093 | 5.94960 | 4.34797 |
| C | 4.76344 | 6.34719 | -0.29272 |
| C | 5.59618 | 7.45873 | -0.00518 |
| C | 5.75215 | 7.94766 | 1.28591 |
| C | 6.87851 | -5.92942 | -4.26169 |
| C | 7.65271 | -6.42541 | -3.19248 |
| C | 7.32608 | -6.06945 | -1.88975 |
| C | 6.23335 | -5.21441 | -1.59546 |
| C | 5.20337 | -4.83396 | -6.26884 |
| C | 6.29122 | -5.68409 | -6.59401 |
| C | 7.12020 | -6.22591 | -5.61928 |
| C | -4.44053 | -4.37227 | 5.73548 |
| C | -5.25715 | -5.36403 | 6.33674 |
| C | -5.84146 | -6.38511 | 5.59736 |
| C | -5.62117 | -6.44586 | 4.20567 |
| C | -6.15526 | -7.44147 | 3.36175 |
| C | -5.87457 | -7.40680 | 2.00142 |
| C | -5.06384 | -6.39491 | 1.42654 |
| C | -6.62754 | 4.48341 | -3.23642 |
| C | -7.67440 | 5.25278 | -3.80610 |
| C | -7.88247 | 5.31222 | -5.17889 |
| C | -7.02968 | 4.59115 | -6.03908 |
| C | -7.13760 | 4.59691 | -7.44419 |
| C | -6.23529 | 3.86021 | -8.20168 |
| C | -5.20363 | 3.09194 | -7.60401 |
| C | 6.62092 | 9.10993 | 1.59147 |
| N | 6.70585 | 9.49350 | 2.94778 |
| C | 6.00623 | 8.90763 | 4.02555 |
| C | 8.26072 | -7.11295 | -5.95865 |
| N | 9.03313 | -7.58092 | -4.87240 |
| C | 8.79141 | -7.32262 | -3.50515 |
| C | -8.97065 | 6.12294 | -5.77918 |
| N | -9.10798 | 6.05632 | -7.18605 |
| C | -8.20739 | 5.41388 | -8.06750 |
| C | -6.69496 | -7.42391 | 6.22557 |
| N | -7.22859 | -8.40272 | 5.35807 |
| C | -6.99138 | -8.50448 | 3.97014 |
| O | -6.93104 | -7.43884 | 7.41846 |
| O | -7.45790 | -9.42363 | 3.32455 |
| O | -9.71624 | 6.80804 | -5.10582 |
| O | -8.31128 | 5.53088 | -9.27305 |
| O | 6.11605 | 9.33619 | 5.15828 |
| O | 9.49307 | -7.81841 | -2.64487 |
| C | 7.55109 | 10.67460 | 3.23797 |
| C | 10.15557 | -8.49823 | -5.17230 |
| C | -10.16189 | 6.90850 | -7.79767 |
| C | -8.04492 | -9.48312 | 5.95958 |
| C | 9.06501 | 10.38761 | 3.32040 |
| C | 11.56286 | -7.86801 | -5.13290 |
| C | -11.54729 | 6.27276 | -8.08358 |
| C | -9.56769 | -9.34160 | 5.76801 |
| O | 7.23631 | 9.71395 | 0.73424 |

| | | | |
|---|---|---|---|
| O | 8.52755 | -7.42073 | -7.10445 |
| C | 13.17543 | 23.66119 | 10.04727 |
| C | 13.37725 | 22.70339 | 8.86955 |
| C | 12.45548 | 21.47942 | 8.91803 |
| C | 12.65098 | 20.51444 | 7.74330 |
| C | 11.72996 | 19.29000 | 7.79327 |
| C | 11.92725 | 18.32427 | 6.61922 |
| C | 11.01018 | 17.09701 | 6.67175 |
| C | 11.21075 | 16.12969 | 5.49955 |
| C | 10.30227 | 14.89633 | 5.55772 |
| C | 10.50852 | 13.92678 | 4.38847 |
| C | 9.61778 | 12.68030 | 4.45865 |
| C | 9.84078 | 11.72449 | 3.27710 |
| C | 10.87293 | 4.68618 | -7.86196 |
| C | 11.26041 | 3.28882 | -8.35422 |
| C | 11.02399 | 2.19094 | -7.31075 |
| C | 11.40291 | 0.78725 | -7.79587 |
| C | 11.16704 | -0.30892 | -6.75048 |
| C | 11.53412 | -1.71482 | -7.23888 |
| C | 11.29742 | -2.80995 | -6.19260 |
| C | 11.64495 | -4.21867 | -6.68708 |
| C | 11.40659 | -5.31360 | -5.64073 |
| C | 11.75460 | -6.71897 | -6.15258 |
| C | -22.44371 | 3.64152 | 1.23798 |
| C | -20.91475 | 3.57696 | 1.18204 |
| C | -20.34852 | 3.80587 | -0.22405 |
| C | -18.81848 | 3.74476 | -0.29094 |
| C | -18.25664 | 3.97240 | -1.69899 |
| C | -16.72641 | 3.91789 | -1.77101 |
| C | -16.17298 | 4.14887 | -3.18189 |
| C | -14.64287 | 4.10775 | -3.26372 |
| C | -14.10161 | 4.34599 | -4.67848 |
| C | -12.56987 | 4.32459 | -4.75930 |
| C | -11.99715 | 4.49827 | -6.17629 |
| C | -12.31954 | 5.86798 | -6.80097 |
| C | -8.26217 | -2.85256 | 14.98695 |
| C | -8.29356 | -3.17770 | 13.49081 |
| C | -9.71257 | -3.26905 | 12.91838 |
| C | -9.75181 | -3.59491 | 11.42110 |
| C | -11.16989 | -3.68879 | 10.84688 |
| C | -11.20347 | -4.01401 | 9.34839 |
| C | -12.62699 | -4.12125 | 8.78694 |
| C | -12.74987 | -4.29938 | 7.26357 |
| C | -12.18996 | -5.61354 | 6.67789 |
| C | -10.71812 | -5.55893 | 6.23799 |
| C | -10.21142 | -6.83016 | 5.53754 |
| C | -10.15424 | -8.07412 | 6.43902 |
| C | -4.64850 | -20.75125 | 6.47161 |
| C | -5.08352 | -19.45046 | 7.15265 |
| C | -5.94498 | -18.55362 | 6.25627 |
| C | -6.38118 | -17.24803 | 6.93055 |
| C | -7.24698 | -16.35126 | 6.03835 |
| C | -7.67318 | -15.04345 | 6.71520 |
| C | -8.54729 | -14.14590 | 5.83188 |
| C | -8.95736 | -12.83458 | 6.51130 |
| C | -9.86467 | -11.94986 | 5.64409 |
| C | -10.27646 | -10.60953 | 6.29972 |
| C | -22.13593 | 7.87449 | -7.02170 |
| C | -21.12752 | 6.73734 | -7.20894 |
| C | -19.78676 | 7.20388 | -7.78777 |
| C | -18.77155 | 6.07139 | -7.97734 |
| C | -17.43062 | 6.53555 | -8.55764 |
| C | -16.41950 | 5.39905 | -8.74760 |
| C | -15.07919 | 5.85512 | -9.33442 |
| C | -14.08803 | 4.70372 | -9.55808 |
| C | -12.72211 | 5.13231 | -10.14701 |

| | | | |
|---|---|---|---|
| C | -11.50584 | 5.20032 | -9.20136 |
| C | 11.21754 | -1.28646 | 8.27778 |
| C | 11.16298 | 0.22379 | 8.52518 |
| C | 10.78570 | 1.03054 | 7.27738 |
| C | 10.73242 | 2.54370 | 7.51597 |
| C | 10.35321 | 3.35152 | 6.26948 |
| C | 10.30584 | 4.86474 | 6.51017 |
| C | 9.91816 | 5.67535 | 5.26813 |
| C | 9.87797 | 7.18819 | 5.51258 |
| C | 9.47263 | 8.00260 | 4.27897 |
| C | 9.47495 | 9.51717 | 4.53399 |
| C | 26.00579 | -13.01320 | -3.54266 |
| C | 24.61507 | -13.26413 | -4.13273 |
| C | 23.61573 | -12.14185 | -3.83014 |
| C | 22.21944 | -12.38642 | -4.41325 |
| C | 21.22100 | -11.26303 | -4.11189 |
| C | 19.82146 | -11.51442 | -4.68464 |
| C | 18.82314 | -10.39005 | -4.38660 |
| C | 17.41818 | -10.65352 | -4.94028 |
| C | 16.41986 | -9.52737 | -4.64900 |
| C | 15.00715 | -9.81024 | -5.17254 |
| C | 14.00914 | -8.68045 | -4.89249 |
| C | 12.58512 | -9.00385 | -5.36126 |
| H | -1.20668 | -0.48012 | 3.54962 |
| H | -1.93607 | -2.53365 | -0.95578 |
| H | 1.58135 | 2.03572 | -1.57063 |
| H | 0.40748 | 1.58463 | 3.27346 |
| H | -2.56545 | 1.59390 | -0.87976 |
| H | -1.12973 | 0.08432 | -5.43026 |
| H | 0.95619 | -1.50023 | -5.16253 |
| H | 2.04411 | -1.87841 | -0.29194 |
| H | 4.49964 | 7.40147 | 5.69119 |
| H | 3.05372 | 5.44064 | 5.13226 |
| H | 4.66713 | 5.99319 | -1.31201 |
| H | 6.13455 | 7.95274 | -0.80509 |
| H | 7.93509 | -6.46556 | -1.08608 |
| H | 6.01034 | -4.95670 | -0.56715 |
| H | 4.57953 | -4.42930 | -7.05666 |
| H | 6.49656 | -5.92788 | -7.62940 |
| H | -3.99993 | -3.59264 | 6.34509 |
| H | -5.44042 | -5.33945 | 7.40417 |
| H | -6.29455 | -8.18466 | 1.37512 |
| H | -4.86790 | -6.39955 | 0.36112 |
| H | -6.49596 | 4.46298 | -2.16133 |
| H | -8.33942 | 5.81851 | -3.16482 |
| H | -6.33546 | 3.88578 | -9.28002 |
| H | -4.51913 | 2.52879 | -8.22682 |
| H | 7.36840 | 11.38729 | 2.43345 |
| H | 7.18416 | 11.08768 | 4.17468 |
| H | 10.10550 | -9.29706 | -4.43263 |
| H | 9.95832 | -8.91138 | -6.16029 |
| H | -10.29566 | 7.74764 | -7.11677 |
| H | -9.74363 | 7.27427 | -8.73459 |
| H | -7.70305 | -10.40866 | 5.50037 |
| H | -7.80084 | -9.49725 | 7.02064 |
| H | 9.33900 | 9.85158 | 2.40526 |
| H | 11.72073 | -7.47828 | -4.12091 |
| H | -12.09411 | 7.13134 | -8.49967 |
| H | -9.76202 | -9.29198 | 4.69049 |
| H | 13.84680 | 24.52191 | 9.98280 |
| H | 12.14961 | 24.04188 | 10.07611 |
| H | 13.36797 | 23.16071 | 11.00146 |
| H | 14.42111 | 22.36758 | 8.84690 |
| H | 13.21175 | 23.24212 | 7.92862 |
| H | 11.41071 | 21.81593 | 8.94087 |
| H | 12.62058 | 20.94099 | 9.86040 |

| | | | |
|---|---|---|---|
| H | 13.69611 | 20.17948 | 7.72081 |
| H | 12.48536 | 21.05345 | 6.80152 |
| H | 10.68472 | 19.62451 | 7.81499 |
| H | 11.89528 | 18.75163 | 8.73545 |
| H | 12.97332 | 17.99234 | 6.59620 |
| H | 11.75925 | 18.86161 | 5.67693 |
| H | 9.96378 | 17.42786 | 6.69326 |
| H | 11.17775 | 16.56091 | 7.61483 |
| H | 12.25889 | 15.80448 | 5.47502 |
| H | 11.03718 | 16.66348 | 4.55625 |
| H | 9.25356 | 15.21959 | 5.58017 |
| H | 10.47549 | 14.36452 | 6.50218 |
| H | 11.56060 | 13.61494 | 4.35904 |
| H | 10.32268 | 14.45370 | 3.44361 |
| H | 8.56847 | 12.99609 | 4.49516 |
| H | 9.81119 | 12.15659 | 5.40199 |
| H | 9.59387 | 12.24547 | 2.34374 |
| H | 10.90937 | 11.48583 | 3.21496 |
| H | 11.05458 | 5.44525 | -8.62781 |
| H | 9.81236 | 4.73134 | -7.59491 |
| H | 11.44834 | 4.96636 | -6.97401 |
| H | 10.69295 | 3.05117 | -9.26229 |
| H | 12.31713 | 3.28529 | -8.64779 |
| H | 9.96708 | 2.19819 | -7.01359 |
| H | 11.59481 | 2.42763 | -6.40330 |
| H | 12.45908 | 0.78058 | -8.09477 |
| H | 10.83031 | 0.55139 | -8.70226 |
| H | 11.74531 | -0.07689 | -5.84674 |
| H | 10.11233 | -0.29668 | -6.44627 |
| H | 10.95421 | -1.94664 | -8.14161 |
| H | 12.58824 | -1.72834 | -7.54459 |
| H | 11.88624 | -2.58490 | -5.29390 |
| H | 10.24566 | -2.78718 | -5.87891 |
| H | 12.69600 | -4.24299 | -7.00297 |
| H | 11.05362 | -4.44375 | -7.58369 |
| H | 10.36147 | -5.27208 | -5.31673 |
| H | 12.00690 | -5.09901 | -4.74718 |
| H | 11.17028 | -6.93712 | -7.05310 |
| H | 12.80296 | -6.70324 | -6.47000 |
| H | -22.81449 | 3.47026 | 2.25235 |
| H | -22.81087 | 4.61950 | 0.91126 |
| H | -22.89539 | 2.88589 | 0.58755 |
| H | -20.57721 | 2.60107 | 1.55192 |
| H | -20.49267 | 4.32287 | 1.86648 |
| H | -20.68828 | 4.78185 | -0.59466 |
| H | -20.77115 | 3.05875 | -0.90851 |
| H | -18.47899 | 2.76934 | 0.08098 |
| H | -18.39653 | 4.49315 | 0.39233 |
| H | -18.60022 | 4.94628 | -2.07120 |
| H | -18.67724 | 3.22231 | -2.38119 |
| H | -16.38068 | 2.94407 | -1.40069 |
| H | -16.30514 | 4.66840 | -1.08972 |
| H | -16.52674 | 5.11984 | -3.55226 |
| H | -16.59140 | 3.39511 | -3.86145 |
| H | -14.28550 | 3.13696 | -2.89627 |
| H | -14.22368 | 4.86203 | -2.58537 |
| H | -14.47960 | 5.30800 | -5.04303 |
| H | -14.50918 | 3.58203 | -5.35406 |
| H | -12.15975 | 5.11033 | -4.11315 |
| H | -12.21023 | 3.37224 | -4.35021 |
| H | -12.38276 | 3.69553 | -6.81554 |
| H | -10.91542 | 4.34570 | -6.13229 |
| H | -13.38848 | 5.89877 | -7.02493 |
| H | -12.14371 | 6.64167 | -6.04679 |
| H | -7.23686 | -2.79801 | 15.36324 |
| H | -8.74287 | -1.89107 | 15.19344 |

| | | | |
|---|---|---|---|
| H | -8.78880 | -3.61574 | 15.56854 |
| H | -7.77175 | -4.12578 | 13.31249 |
| H | -7.73126 | -2.41393 | 12.93975 |
| H | -10.23515 | -2.32018 | 13.09744 |
| H | -10.27520 | -4.03323 | 13.47037 |
| H | -9.22712 | -4.54246 | 11.24355 |
| H | -9.18939 | -2.82955 | 10.87048 |
| H | -11.73236 | -4.45535 | 11.39564 |
| H | -11.69630 | -2.74189 | 11.02416 |
| H | -10.66219 | -4.95153 | 9.18017 |
| H | -10.65316 | -3.23951 | 8.79829 |
| H | -13.17885 | -3.21506 | 9.06719 |
| H | -13.14529 | -4.95227 | 9.28331 |
| H | -12.27853 | -3.44650 | 6.75795 |
| H | -13.81539 | -4.23804 | 7.01620 |
| H | -12.78791 | -5.88796 | 5.80007 |
| H | -12.34065 | -6.41968 | 7.40521 |
| H | -10.59872 | -4.71296 | 5.54992 |
| H | -10.07144 | -5.34446 | 7.09491 |
| H | -10.85126 | -7.04681 | 4.67169 |
| H | -9.21779 | -6.62290 | 5.12925 |
| H | -9.58390 | -7.84253 | 7.34536 |
| H | -11.17148 | -8.30529 | 6.77043 |
| H | -4.03839 | -21.36729 | 7.13797 |
| H | -5.51469 | -21.34787 | 6.16855 |
| H | -4.05729 | -20.54888 | 5.57293 |
| H | -4.19586 | -18.89207 | 7.47392 |
| H | -5.64053 | -19.68637 | 8.06760 |
| H | -6.83461 | -19.11180 | 5.93650 |
| H | -5.38871 | -18.31971 | 5.33912 |
| H | -5.49036 | -16.69011 | 7.24705 |
| H | -6.93323 | -17.48332 | 7.84976 |
| H | -8.14134 | -16.90620 | 5.72687 |
| H | -6.69800 | -16.11922 | 5.11661 |
| H | -6.77749 | -14.48725 | 7.02079 |
| H | -8.21514 | -15.27611 | 7.64104 |
| H | -9.44895 | -14.69721 | 5.53506 |
| H | -8.01126 | -13.91881 | 4.90144 |
| H | -8.05462 | -12.28146 | 6.79733 |
| H | -9.47828 | -13.06146 | 7.45048 |
| H | -10.76759 | -12.52275 | 5.40618 |
| H | -9.37773 | -11.75434 | 4.68126 |
| H | -11.34960 | -10.45392 | 6.15319 |
| H | -10.13341 | -10.66999 | 7.38690 |
| H | -23.07892 | 7.50896 | -6.60585 |
| H | -21.74763 | 8.63934 | -6.34175 |
| H | -22.36107 | 8.36427 | -7.97429 |
| H | -21.55758 | 5.97302 | -7.86761 |
| H | -20.95132 | 6.24295 | -6.24589 |
| H | -19.96310 | 7.69841 | -8.75194 |
| H | -19.35706 | 7.96979 | -7.12894 |
| H | -19.20336 | 5.30586 | -8.63504 |
| H | -18.59604 | 5.57790 | -7.01276 |
| H | -16.99735 | 7.30027 | -7.90004 |
| H | -17.60568 | 7.02928 | -9.52226 |
| H | -16.85605 | 4.63338 | -9.40219 |
| H | -16.24322 | 4.90661 | -7.78265 |
| H | -15.25724 | 6.35809 | -10.29369 |
| H | -14.63529 | 6.61302 | -8.67755 |
| H | -13.93886 | 4.16004 | -8.61874 |
| H | -14.55956 | 3.98610 | -10.23926 |
| H | -12.83619 | 6.10599 | -10.64091 |
| H | -12.45260 | 4.43347 | -10.94601 |
| H | -11.33144 | 4.21013 | -8.76656 |
| H | -10.63373 | 5.39914 | -9.82684 |
| H | 11.48981 | -1.83123 | 9.18599 |

| | | | |
|---|---|---|---|
| H | 10.24847 | -1.66741 | 7.94030 |
| H | 11.95525 | -1.53479 | 7.50825 |
| H | 12.13607 | 0.56824 | 8.89583 |
| H | 10.44202 | 0.43717 | 9.32370 |
| H | 9.81085 | 0.68706 | 6.90712 |
| H | 11.50614 | 0.81466 | 6.47762 |
| H | 11.70805 | 2.88561 | 7.88495 |
| H | 10.01375 | 2.75827 | 8.31749 |
| H | 9.37568 | 3.01257 | 5.90258 |
| H | 11.06967 | 3.13495 | 5.46656 |
| H | 11.28532 | 5.20368 | 6.87159 |
| H | 9.59437 | 5.08052 | 7.31768 |
| H | 8.93584 | 5.34018 | 4.91062 |
| H | 10.62609 | 5.45725 | 4.45806 |
| H | 10.86369 | 7.52432 | 5.85989 |
| H | 9.17798 | 7.40506 | 6.32920 |
| H | 8.48341 | 7.67212 | 3.94567 |
| H | 10.15936 | 7.77853 | 3.45231 |
| H | 8.82912 | 9.74096 | 5.38882 |
| H | 10.49072 | 9.79965 | 4.83663 |
| H | 26.69337 | -13.83066 | -3.77655 |
| H | 26.44176 | -12.08974 | -3.93673 |
| H | 25.96171 | -12.91784 | -2.45317 |
| H | 24.22022 | -14.21175 | -3.74661 |
| H | 24.69709 | -13.39039 | -5.21915 |
| H | 24.01025 | -11.19382 | -4.21865 |
| H | 23.53637 | -12.01338 | -2.74273 |
| H | 21.82583 | -13.33379 | -4.02260 |
| H | 22.29953 | -12.51721 | -5.50020 |
| H | 21.61060 | -10.31688 | -4.50939 |
| H | 21.14688 | -11.12712 | -3.02516 |
| H | 19.43167 | -12.45892 | -4.28337 |
| H | 19.89486 | -11.65452 | -5.77087 |
| H | 19.20544 | -9.44787 | -4.80031 |
| H | 18.76038 | -10.24062 | -3.30095 |
| H | 17.03544 | -11.59252 | -4.51978 |
| H | 17.47993 | -10.81067 | -6.02489 |
| H | 16.79042 | -8.59251 | -5.08915 |
| H | 16.37452 | -9.35528 | -3.56590 |
| H | 14.63623 | -10.73994 | -4.72193 |
| H | 15.05101 | -9.99433 | -6.25372 |
| H | 14.36670 | -7.76181 | -5.37228 |
| H | 13.98811 | -8.47322 | -3.81516 |
| H | 12.59748 | -9.26576 | -6.42767 |
| H | 12.24959 | -9.90392 | -4.83162 |

**4PIPBSP-dipb (E=-7766.12610198 A.U.)**

| | | | |
|---|---|---|---|
| C | 0.58860 | -0.48380 | -2.33217 |
| C | 0.93175 | -0.78103 | -0.98215 |
| C | 0.02151 | -0.01837 | -0.01454 |
| C | -0.88687 | 0.74432 | -0.98435 |
| C | -0.53106 | 0.45868 | -2.33350 |
| C | 0.78373 | 0.89402 | 0.95194 |
| C | 0.48651 | 0.55286 | 2.30236 |
| C | -0.45625 | -0.56655 | 2.30508 |
| C | -0.74267 | -0.92375 | 0.95636 |
| C | 10.46130 | -8.73691 | -3.32797 |
| C | 9.44813 | -7.89143 | -2.87292 |
| C | 8.61387 | -7.20237 | -3.75035 |
| C | 8.80852 | -7.36950 | -5.16217 |
| C | 9.84830 | -8.23888 | -5.61440 |
| C | 10.66711 | -8.91831 | -4.68264 |

| | | | |
|---|---|---|---|
| C | 7.99859 | -6.69728 | -6.13731 |
| C | 8.25516 | -6.91793 | -7.48863 |
| C | 9.27342 | -7.77106 | -7.91714 |
| C | 10.06903 | -8.42920 | -6.99830 |
| C | 7.54540 | -6.31275 | -3.28249 |
| C | 6.75559 | -5.65502 | -4.27005 |
| C | 6.93072 | -5.80295 | -5.67679 |
| C | 5.73898 | -4.80581 | -3.82830 |
| C | 4.88268 | -4.08953 | -4.69634 |
| C | 5.05636 | -4.23570 | -6.05742 |
| C | 6.07662 | -5.08969 | -6.52606 |
| C | 7.25280 | -6.06923 | -1.93529 |
| C | 6.21961 | -5.20750 | -1.51134 |
| C | 5.45289 | -4.56705 | -2.46361 |
| C | 4.32746 | -3.62587 | -2.44657 |
| C | 3.97487 | -3.32866 | -3.82912 |
| C | 11.13611 | -9.33380 | -7.48990 |
| N | 11.92224 | -9.97840 | -6.51745 |
| C | 11.75423 | -9.82269 | -5.12942 |
| O | 12.47342 | -10.40653 | -4.34177 |
| O | 11.32984 | -9.51996 | -8.67576 |
| C | 7.74933 | 9.26841 | 7.87207 |
| C | 6.90127 | 8.24505 | 7.44530 |
| C | 6.68537 | 7.98230 | 6.09449 |
| C | 7.35917 | 8.78929 | 5.11778 |
| C | 8.22205 | 9.83546 | 5.56786 |
| C | 8.40507 | 10.06413 | 6.95194 |
| C | 7.20215 | 8.58316 | 3.70628 |
| C | 7.90188 | 9.40628 | 2.82690 |
| C | 8.74654 | 10.42124 | 3.27960 |
| C | 8.91056 | 10.64428 | 4.63348 |
| C | 5.79553 | 6.90969 | 5.63602 |
| C | 5.65239 | 6.72780 | 4.22966 |
| C | 6.31292 | 7.51336 | 3.24059 |
| C | 4.80488 | 5.70890 | 3.78987 |
| C | 4.56897 | 5.41791 | 2.42575 |
| C | 5.21087 | 6.18154 | 1.47201 |
| C | 6.07180 | 7.21618 | 1.89398 |
| C | 5.08089 | 6.05836 | 6.48688 |
| C | 4.22965 | 5.03486 | 6.02021 |
| C | 4.08714 | 4.85541 | 4.65949 |
| C | 3.32859 | 3.94401 | 3.79392 |
| C | 3.62783 | 4.29249 | 2.41078 |
| C | 9.81591 | 11.73130 | 5.07833 |
| N | 9.89630 | 11.96037 | 6.46643 |
| C | 9.29869 | 11.14220 | 7.44166 |
| O | 9.50725 | 11.32467 | 8.62570 |
| O | 10.45757 | 12.40384 | 4.29576 |
| C | -8.70457 | -10.43961 | 3.33964 |
| C | -7.85441 | -9.43216 | 2.88046 |
| C | -7.17711 | -8.58454 | 3.75421 |
| C | -7.36086 | -8.75959 | 5.16655 |
| C | -8.23619 | -9.79260 | 5.62285 |
| C | -8.90224 | -10.62609 | 4.69466 |
| C | -6.69974 | -7.93643 | 6.13818 |
| C | -6.93677 | -8.17377 | 7.49024 |
| C | -7.79670 | -9.18453 | 7.92282 |
| C | -8.44554 | -9.99184 | 7.00742 |
| C | -6.28395 | -7.52088 | 3.28206 |
| C | -5.63676 | -6.71828 | 4.26628 |
| C | -5.79932 | -6.87553 | 5.67351 |
| C | -4.78377 | -5.70658 | 3.82054 |
| C | -4.07649 | -4.83941 | 4.68514 |
| C | -4.23585 | -4.99660 | 6.04674 |
| C | -5.09447 | -6.01110 | 6.51934 |
| C | -6.02792 | -7.24401 | 1.93386 |

| | | | |
|---|---|---|---|
| C | -5.16332 | -6.21488 | 1.50581 |
| C | -4.53243 | -5.43632 | 2.45487 |
| C | -3.59261 | -4.30979 | 2.43345 |
| C | -3.30839 | -3.94106 | 3.81453 |
| C | -9.35626 | -11.05218 | 7.50370 |
| N | -9.99118 | -11.84841 | 6.53800 |
| C | -9.81650 | -11.70281 | 5.14547 |
| O | -10.39499 | -12.43021 | 4.36390 |
| O | -9.55174 | -11.23019 | 8.69190 |
| C | -9.21363 | 7.73588 | -7.93559 |
| C | -8.18927 | 6.89111 | -7.50483 |
| C | -7.95120 | 6.65143 | -6.15342 |
| C | -8.78624 | 7.29561 | -5.18031 |
| C | -9.83181 | 8.15701 | -5.63454 |
| C | -10.03177 | 8.36849 | -7.01894 |
| C | -8.61340 | 7.10464 | -3.76853 |
| C | -9.48000 | 7.75529 | -2.89326 |
| C | -10.50404 | 8.58660 | -3.35028 |
| C | -10.68433 | 8.79667 | -4.70423 |
| C | -6.87994 | 5.76210 | -5.69086 |
| C | -6.72176 | 5.59673 | -4.28407 |
| C | -7.53437 | 6.22904 | -3.29841 |
| C | -5.69969 | 4.75518 | -3.84026 |
| C | -5.42679 | 4.50367 | -2.47519 |
| C | -6.21288 | 5.12286 | -1.52466 |
| C | -7.25354 | 5.97448 | -1.95077 |
| C | -6.00721 | 5.06949 | -6.53825 |
| C | -4.98379 | 4.22032 | -6.06764 |
| C | -4.82496 | 4.05919 | -4.70644 |
| C | -3.91984 | 3.29699 | -3.83750 |
| C | -4.29070 | 3.57549 | -2.45584 |
| C | -11.78182 | 9.68787 | -5.15337 |
| N | -11.88074 | 9.92174 | -6.53655 |
| C | -11.10959 | 9.25945 | -7.51237 |
| O | -11.32492 | 9.42473 | -8.69681 |
| O | -12.55960 | 10.20338 | -4.37358 |
| C | -1.91802 | 1.59816 | -0.69023 |
| C | -2.63875 | 2.20634 | -1.74596 |
| C | -2.27668 | 1.92559 | -3.10988 |
| C | -1.21189 | 1.04094 | -3.38182 |
| C | 1.27687 | -1.05980 | -3.37905 |
| C | 2.33457 | -1.95217 | -3.10483 |
| C | 2.68097 | -2.24834 | -1.74010 |
| C | 1.95496 | -1.64369 | -0.68588 |
| C | 1.64660 | 1.91652 | 0.65401 |
| C | 2.25093 | 2.64437 | 1.70707 |
| C | 1.95392 | 2.30089 | 3.07236 |
| C | 1.06202 | 1.24318 | 3.34820 |
| C | -1.03944 | -1.24488 | 3.35445 |
| C | -1.92791 | -2.30685 | 3.08415 |
| C | -2.21191 | -2.66856 | 1.72082 |
| C | -1.60053 | -1.95211 | 0.66393 |
| N | 3.70190 | -3.10492 | -1.42558 |
| N | 3.00828 | -2.51650 | -4.15809 |
| N | -2.94322 | 2.49605 | -4.16442 |
| N | -3.67073 | 3.05034 | -1.43361 |
| N | -2.50042 | -2.96974 | 4.13984 |
| N | -3.06372 | -3.69468 | 1.41018 |
| N | 2.51664 | 2.97776 | 4.12449 |
| N | 3.10796 | 3.66435 | 1.39084 |
| C | -10.89764 | -12.89866 | 6.98847 |
| C | -10.38545 | -14.19148 | 7.22068 |
| C | -11.29239 | -15.18758 | 7.60070 |
| C | -12.64454 | -14.92489 | 7.75196 |
| C | -13.11703 | -13.64149 | 7.52964 |
| C | -12.26576 | -12.59798 | 7.14791 |

| | | | |
|---|---|---|---|
| C | -8.91445 | -14.63021 | 7.17406 |
| C | -8.19476 | -14.32603 | 8.50449 |
| C | -8.06797 | -14.19012 | 5.96771 |
| C | -12.93670 | -11.22221 | 7.02059 |
| C | -12.81964 | -10.42016 | 8.33351 |
| C | -12.58089 | -10.35436 | 5.80186 |
| C | 12.97948 | -10.86944 | -6.98080 |
| C | 14.24779 | -10.32984 | -7.27769 |
| C | 15.23932 | -11.21366 | -7.71902 |
| C | 14.99389 | -12.56854 | -7.87361 |
| C | 13.73628 | -13.06945 | -7.57846 |
| C | 12.70190 | -12.24430 | -7.12184 |
| C | 14.67098 | -8.85447 | -7.22064 |
| C | 14.21541 | -8.02187 | -6.01042 |
| C | 14.37224 | -8.12602 | -8.54756 |
| C | 11.39312 | -12.97281 | -6.78222 |
| C | 11.36366 | -13.41833 | -5.30521 |
| C | 10.06844 | -12.30725 | -7.19180 |
| C | 10.79374 | 13.01474 | 6.91850 |
| C | 10.29761 | 14.31086 | 7.15108 |
| C | 11.21800 | 15.28133 | 7.56312 |
| C | 12.56251 | 14.98723 | 7.74304 |
| C | 13.02118 | 13.69950 | 7.51097 |
| C | 12.14978 | 12.68998 | 7.09444 |
| C | 8.83744 | 14.76390 | 7.05057 |
| C | 8.04434 | 14.41879 | 8.32722 |
| C | 8.06714 | 14.35425 | 5.78311 |
| C | 12.70353 | 11.29103 | 6.84782 |
| C | 13.31809 | 10.69358 | 8.12725 |
| C | 13.70402 | 11.28275 | 5.67690 |
| C | -12.95985 | 10.77970 | -7.00633 |
| C | -12.74488 | 12.16136 | -7.16275 |
| C | -13.81948 | 12.92461 | -7.63327 |
| C | -15.04900 | 12.35395 | -7.93153 |
| C | -15.23174 | 10.98948 | -7.76502 |
| C | -14.19441 | 10.17631 | -7.30196 |
| C | -11.46489 | 12.93341 | -6.82619 |
| C | -10.13965 | 12.33700 | -7.33161 |
| C | -11.38889 | 13.28410 | -5.32641 |
| C | -14.44332 | 8.68074 | -7.14064 |
| C | -15.55709 | 8.40182 | -6.11440 |
| C | -14.73655 | 8.01185 | -8.49662 |
| H | 11.10134 | -9.26355 | -2.63139 |
| H | 9.31966 | -7.77815 | -1.80480 |
| H | 7.65563 | -6.42160 | -8.23995 |
| H | 9.45691 | -7.93264 | -8.97189 |
| H | 4.42586 | -3.70841 | -6.76345 |
| H | 6.19008 | -5.18539 | -7.59809 |
| H | 7.83790 | -6.55761 | -1.16685 |
| H | 6.04006 | -5.05828 | -0.45327 |
| H | 7.90751 | 9.45691 | 8.92650 |
| H | 6.40499 | 7.64678 | 8.19765 |
| H | 7.79929 | 9.26629 | 1.75915 |
| H | 9.28577 | 11.04947 | 2.58188 |
| H | 5.06319 | 5.99864 | 0.41430 |
| H | 6.56072 | 7.79931 | 1.12439 |
| H | 5.17274 | 6.17699 | 7.55868 |
| H | 3.70109 | 4.40683 | 6.72749 |
| H | -9.22335 | -11.08933 | 2.64615 |
| H | -7.72830 | -9.31870 | 1.81207 |
| H | -6.44843 | -7.56452 | 8.23896 |
| H | -7.97031 | -9.35336 | 8.97810 |
| H | -3.71568 | -4.35731 | 6.75014 |
| H | -5.20098 | -6.11132 | 7.59166 |
| H | -6.50877 | -7.83843 | 1.16783 |
| H | -5.00461 | -6.04752 | 0.44713 |

| | | | |
|---|---|---|---|
| H | -9.38518 | 7.90993 | -8.99039 |
| H | -7.57085 | 6.41585 | -8.25447 |
| H | -9.37040 | 7.62065 | -1.82551 |
| H | -11.17199 | 9.08042 | -2.65579 |
| H | -6.04326 | 4.96409 | -0.46635 |
| H | -7.85399 | 6.44604 | -1.18366 |
| H | -6.10824 | 5.17773 | -7.61033 |
| H | -4.33918 | 3.70851 | -6.77232 |
| H | -2.21332 | 1.83207 | 0.32579 |
| H | -0.96498 | 0.84707 | -4.41884 |
| H | 1.04020 | -0.85636 | -4.41661 |
| H | 2.23916 | -1.88824 | 0.33080 |
| H | 1.89149 | 2.19889 | -0.36309 |
| H | 0.85807 | 1.00850 | 4.38612 |
| H | -0.84459 | -0.99714 | 4.39108 |
| H | -1.83661 | -2.24722 | -0.35164 |
| H | -10.91872 | -16.19026 | 7.77812 |
| H | -13.32714 | -15.71734 | 8.03819 |
| H | -14.17424 | -13.43157 | 7.65118 |
| H | -8.96961 | -15.72151 | 7.10205 |
| H | -7.18728 | -14.75374 | 8.49035 |
| H | -8.11226 | -13.25415 | 8.68725 |
| H | -8.73625 | -14.75875 | 9.34919 |
| H | -7.17294 | -14.81686 | 5.91583 |
| H | -8.61361 | -14.30037 | 5.02951 |
| H | -7.72489 | -13.15706 | 6.04935 |
| H | -13.99921 | -11.45939 | 6.90300 |
| H | -13.39615 | -9.49266 | 8.25844 |
| H | -13.20835 | -10.99509 | 9.17757 |
| H | -11.78434 | -10.16628 | 8.56377 |
| H | -12.55036 | -10.94128 | 4.88275 |
| H | -13.34391 | -9.57933 | 5.68429 |
| H | -11.62515 | -9.83970 | 5.91669 |
| H | 16.22321 | -10.81835 | -7.94782 |
| H | 15.77880 | -13.23063 | -8.22202 |
| H | 13.54054 | -14.12960 | -7.69864 |
| H | 15.76228 | -8.90121 | -7.14056 |
| H | 14.83646 | -7.12384 | -5.94470 |
| H | 13.18110 | -7.68397 | -6.09664 |
| H | 14.32267 | -8.57517 | -5.07599 |
| H | 13.30126 | -8.04738 | -8.73612 |
| H | 14.79576 | -7.11695 | -8.52283 |
| H | 14.81311 | -8.65920 | -9.39333 |
| H | 11.44641 | -13.89176 | -7.37564 |
| H | 10.47865 | -14.03474 | -5.11807 |
| H | 12.24858 | -14.01016 | -5.05868 |
| H | 11.34323 | -12.56617 | -4.62514 |
| H | 10.12004 | -11.88883 | -8.19830 |
| H | 9.27584 | -13.06108 | -7.18016 |
| H | 9.76166 | -11.51822 | -6.50277 |
| H | 10.86250 | 16.28991 | 7.74542 |
| H | 13.25058 | 15.76287 | 8.06076 |
| H | 14.07167 | 13.47236 | 7.65114 |
| H | 8.89912 | 15.85684 | 7.01575 |
| H | 7.94577 | 13.34159 | 8.46728 |
| H | 7.04064 | 14.85225 | 8.27378 |
| H | 8.54084 | 14.81720 | 9.21526 |
| H | 7.17716 | 14.98266 | 5.68554 |
| H | 7.72327 | 13.31911 | 5.82194 |
| H | 8.67296 | 14.48142 | 4.88402 |
| H | 11.87584 | 10.63928 | 6.56317 |
| H | 13.64335 | 9.66450 | 7.94715 |
| H | 14.19123 | 11.26341 | 8.45741 |
| H | 12.59035 | 10.68734 | 8.94166 |
| H | 14.58723 | 11.88708 | 5.90285 |
| H | 13.24365 | 11.68242 | 4.77093 |

| | | | |
|---|---|---|---|
| H | 14.04270 | 10.26254 | 5.47229 |
| H | -13.67929 | 13.99202 | -7.76669 |
| H | -15.86109 | 12.97289 | -8.29680 |
| H | -16.19224 | 10.54571 | -8.00024 |
| H | -11.58028 | 13.88466 | -7.35675 |
| H | -9.36864 | 13.11282 | -7.31857 |
| H | -9.78191 | 11.52282 | -6.69881 |
| H | -10.22892 | 11.96429 | -8.35361 |
| H | -10.52571 | 13.92954 | -5.13545 |
| H | -12.28849 | 13.81331 | -5.00301 |
| H | -11.29596 | 12.39313 | -4.70400 |
| H | -13.53408 | 8.22081 | -6.74963 |
| H | -15.67308 | 7.32438 | -5.96315 |
| H | -16.52086 | 8.79466 | -6.45063 |
| H | -15.32233 | 8.86107 | -5.15158 |
| H | -13.92695 | 8.20051 | -9.20463 |
| H | -14.84331 | 6.92988 | -8.37236 |
| H | -15.66521 | 8.38875 | -8.93467 |

## 4PIPBSP-dt (E=-9673.09478270 A.U.)

| | | | |
|---|---|---|---|
| C | -0.13175 | 0.72061 | 2.23516 |
| C | -0.12216 | 1.16186 | 0.88099 |
| C | -0.67970 | 0.07545 | -0.04385 |
| C | -1.01024 | -1.03786 | 0.95514 |
| C | -0.67748 | -0.63673 | 2.28064 |
| C | 0.29654 | -0.36154 | -1.14156 |
| C | -0.28016 | -0.16748 | -2.42915 |
| C | -1.62300 | 0.38838 | -2.26200 |
| C | -1.88387 | 0.53960 | -0.87022 |
| C | 4.37208 | 12.79640 | 2.87274 |
| C | 3.89470 | 11.55306 | 2.45440 |
| C | 3.65096 | 10.51708 | 3.35320 |
| C | 3.90091 | 10.74039 | 4.74864 |
| C | 4.39562 | 12.01497 | 5.16223 |
| C | 4.62501 | 13.03478 | 4.21002 |
| C | 3.66819 | 9.73428 | 5.74484 |
| C | 3.93936 | 10.03601 | 7.07745 |
| C | 4.42683 | 11.28479 | 7.46771 |
| C | 4.65700 | 12.27187 | 6.52842 |
| C | 3.14359 | 9.20911 | 2.92467 |
| C | 2.91454 | 8.22814 | 3.93308 |
| C | 3.15012 | 8.42747 | 5.32471 |
| C | 2.41974 | 6.98618 | 3.52984 |
| C | 2.13709 | 5.92661 | 4.42278 |
| C | 2.36660 | 6.12447 | 5.76908 |
| C | 2.87014 | 7.37033 | 6.19849 |
| C | 2.86600 | 8.86602 | 1.59613 |
| C | 2.36711 | 7.60412 | 1.21105 |
| C | 2.13776 | 6.65281 | 2.18431 |
| C | 1.63104 | 5.27633 | 2.20797 |
| C | 1.62863 | 4.82637 | 3.59420 |
| C | 5.17310 | 13.58846 | 6.97802 |
| N | 5.40389 | 14.55142 | 5.98565 |
| C | 5.12644 | 14.36678 | 4.62371 |
| O | 5.29658 | 15.27243 | 3.82655 |
| O | 5.38744 | 13.82719 | 8.15391 |
| C | 5.89909 | 15.88469 | 6.39234 |
| C | 7.38900 | 16.12206 | 6.06926 |
| C | 9.34038 | -4.63369 | -9.29387 |
| C | 8.19678 | -4.08252 | -8.71328 |
| C | 8.05039 | -3.97479 | -7.33226 |
| C | 9.11260 | -4.43979 | -6.48711 |

| | | | |
|---|---|---|---|
| C | 10.27310 | -5.01025 | -7.09398 |
| C | 10.37319 | -5.09844 | -8.50219 |
| C | 9.05110 | -4.35922 | -5.05558 |
| C | 10.12433 | -4.84316 | -4.31091 |
| C | 11.24916 | -5.40663 | -4.91640 |
| C | 11.33317 | -5.49442 | -6.29270 |
| C | 6.84794 | -3.40756 | -6.71223 |
| C | 6.81195 | -3.33436 | -5.28925 |
| C | 7.85885 | -3.77719 | -4.42921 |
| C | 5.66816 | -2.79623 | -4.69589 |
| C | 5.49584 | -2.66472 | -3.29781 |
| C | 6.51582 | -3.08798 | -2.46985 |
| C | 7.67983 | -3.63674 | -3.04788 |
| C | 5.73734 | -2.94074 | -7.42503 |
| C | 4.58977 | -2.40490 | -6.80426 |
| C | 4.55102 | -2.33052 | -5.42703 |
| C | 3.58034 | -1.86783 | -4.42824 |
| C | 4.16619 | -2.07284 | -3.10954 |
| C | 12.53163 | -6.11553 | -6.90592 |
| N | 12.58573 | -6.15353 | -8.30609 |
| C | 11.56825 | -5.68981 | -9.15242 |
| O | 11.66648 | -5.77328 | -10.36416 |
| O | 13.43753 | -6.57939 | -6.23628 |
| C | 13.75710 | -6.82148 | -8.91269 |
| C | 15.05038 | -5.98421 | -8.97332 |
| C | -13.47071 | 5.49280 | -1.83585 |
| C | -12.22324 | 4.94747 | -1.52630 |
| C | -11.34460 | 4.50911 | -2.51442 |
| C | -11.74094 | 4.62256 | -3.88891 |
| C | -13.02210 | 5.17696 | -4.19096 |
| C | -13.87774 | 5.60965 | -3.15131 |
| C | -10.89733 | 4.20575 | -4.97225 |
| C | -11.36071 | 4.35217 | -6.27776 |
| C | -12.61598 | 4.89425 | -6.55910 |
| C | -13.44587 | 5.30524 | -5.53370 |
| C | -10.02891 | 3.94064 | -2.20089 |
| C | -9.20713 | 3.53863 | -3.29403 |
| C | -9.57681 | 3.64481 | -4.66649 |
| C | -7.95001 | 3.00669 | -2.99988 |
| C | -7.03122 | 2.57637 | -3.98477 |
| C | -7.39326 | 2.68150 | -5.31204 |
| C | -8.66004 | 3.21273 | -5.63197 |
| C | -9.52840 | 3.77256 | -0.90436 |
| C | -8.25480 | 3.23105 | -0.63020 |
| C | -7.45312 | 2.84434 | -1.68515 |
| C | -6.11150 | 2.26694 | -1.82988 |
| C | -5.84957 | 2.10356 | -3.25419 |
| C | -14.76906 | 5.88482 | -5.86290 |
| N | -15.59023 | 6.26107 | -4.79209 |
| C | -15.20528 | 6.20345 | -3.44456 |
| O | -15.93262 | 6.61804 | -2.55856 |
| O | -15.14415 | 6.03597 | -7.01283 |
| C | -16.89239 | 6.87076 | -5.14355 |
| C | -17.93747 | 5.88322 | -5.74120 |
| C | -4.27759 | -11.21904 | 8.18107 |
| C | -3.82606 | -9.98333 | 7.71400 |
| C | -3.85409 | -9.65189 | 6.36137 |
| C | -4.36273 | -10.61339 | 5.42535 |
| C | -4.82007 | -11.87450 | 5.91641 |
| C | -4.77023 | -12.16253 | 7.30004 |
| C | -4.43367 | -10.35518 | 4.01564 |
| C | -4.94486 | -11.34423 | 3.17836 |
| C | -5.38936 | -12.57230 | 3.67161 |
| C | -5.33191 | -12.84584 | 5.02487 |
| C | -3.38471 | -8.35504 | 5.86078 |
| C | -3.46616 | -8.12185 | 4.45711 |

| | | | |
|---|---|---|---|
| C | -3.96805 | -9.05974 | 3.50830 |
| C | -3.02252 | -6.88763 | 3.97755 |
| C | -3.04573 | -6.52185 | 2.61133 |
| C | -3.53240 | -7.43354 | 1.69668 |
| C | -3.98530 | -8.68685 | 2.15914 |
| C | -2.86531 | -7.33706 | 6.66919 |
| C | -2.42177 | -6.09742 | 6.16260 |
| C | -2.49965 | -5.86640 | 4.80444 |
| C | -2.15879 | -4.75590 | 3.90653 |
| C | -2.49767 | -5.16245 | 2.54868 |
| C | -5.81737 | -14.15918 | 5.51523 |
| N | -5.71542 | -14.39771 | 6.89563 |
| C | -5.24844 | -13.46287 | 7.82705 |
| O | -5.24209 | -13.71924 | 9.01950 |
| O | -6.28186 | -14.99666 | 4.76344 |
| C | -6.22110 | -15.68177 | 7.42646 |
| C | -5.12500 | -16.69581 | 7.81268 |
| C | -1.54814 | -2.27309 | 0.70331 |
| C | -1.77650 | -3.16358 | 1.77969 |
| C | -1.44128 | -2.76162 | 3.11995 |
| C | -0.88871 | -1.48367 | 3.34830 |
| C | 0.31867 | 1.54069 | 3.24827 |
| C | 0.78842 | 2.83363 | 2.93543 |
| C | 0.79442 | 3.27732 | 1.56685 |
| C | 0.33227 | 2.41110 | 0.54713 |
| C | 1.55714 | -0.88217 | -1.00241 |
| C | 2.29511 | -1.23369 | -2.15854 |
| C | 1.71553 | -1.03478 | -3.46035 |
| C | 0.41726 | -0.49500 | -3.57241 |
| C | -2.56737 | 0.74486 | -3.20111 |
| C | -3.80514 | 1.26865 | -2.77349 |
| C | -4.06533 | 1.42503 | -1.36709 |
| C | -3.07760 | 1.04667 | -0.42578 |
| N | 1.22945 | 4.52738 | 1.21657 |
| N | 1.22210 | 3.64069 | 3.95649 |
| N | -1.64509 | -3.59136 | 4.19327 |
| N | -2.31455 | -4.39334 | 1.50951 |
| N | -4.73186 | 1.61987 | -3.72162 |
| N | -5.24905 | 1.93709 | -0.90638 |
| N | 2.39085 | -1.36325 | -4.60805 |
| N | 3.54770 | -1.76454 | -2.00142 |
| C | 6.62984 | 28.22405 | 1.81401 |
| C | 7.20910 | 27.27270 | 2.86497 |
| C | 6.78478 | 25.81370 | 2.66254 |
| C | 7.35703 | 24.85359 | 3.71137 |
| C | 6.93091 | 23.39531 | 3.50760 |
| C | 7.49580 | 22.43538 | 4.56086 |
| C | 7.06895 | 20.97767 | 4.35477 |
| C | 7.62071 | 20.01823 | 5.41541 |
| C | 7.19532 | 18.56057 | 5.20280 |
| C | 7.74446 | 17.61002 | 6.27590 |
| C | 14.00075 | 11.46314 | 19.22742 |
| C | 13.83624 | 11.23507 | 17.72212 |
| C | 12.88260 | 12.23438 | 17.05729 |
| C | 12.71198 | 12.01439 | 15.55012 |
| C | 11.75920 | 13.01474 | 14.88563 |
| C | 11.59208 | 12.79661 | 13.37761 |
| C | 10.64201 | 13.79863 | 12.71180 |
| C | 10.48152 | 13.58463 | 11.20247 |
| C | 9.53444 | 14.58834 | 10.53493 |
| C | 9.38669 | 14.38134 | 9.02353 |
| C | 8.43540 | 15.38286 | 8.35679 |
| C | 8.33958 | 15.17935 | 6.83846 |
| C | 30.09051 | -8.82060 | -10.86295 |
| C | 28.65375 | -9.27376 | -11.13756 |
| C | 27.59724 | -8.33031 | -10.55125 |

| | | | |
|---|---|---|---|
| C | 26.15591 | -8.77637 | -10.82106 |
| C | 25.09895 | -7.83406 | -10.23379 |
| C | 23.65848 | -8.28288 | -10.50470 |
| C | 22.59874 | -7.34443 | -9.91631 |
| C | 21.16007 | -7.79806 | -10.18871 |
| C | 20.09576 | -6.86602 | -9.59836 |
| C | 18.66001 | -7.32756 | -9.87275 |
| C | 17.58974 | -6.40424 | -9.27829 |
| C | 16.16088 | -6.89401 | -9.54346 |
| C | -21.72894 | -0.22136 | 4.93142 |
| C | -22.13280 | 0.61887 | 3.71648 |
| C | -20.94770 | 1.00128 | 2.82236 |
| C | -21.34300 | 1.84294 | 1.60380 |
| C | -20.15918 | 2.22482 | 0.70782 |
| C | -20.55865 | 3.06765 | -0.50882 |
| C | -19.37901 | 3.45057 | -1.40973 |
| C | -19.78670 | 4.29470 | -2.62275 |
| C | -18.61093 | 4.68245 | -3.52797 |
| C | -19.04769 | 5.52340 | -4.73455 |
| C | -25.96879 | 3.94585 | -18.76874 |
| C | -25.11199 | 4.89139 | -17.92191 |
| C | -24.63173 | 4.26321 | -16.60867 |
| C | -23.77487 | 5.20306 | -15.75302 |
| C | -23.29850 | 4.57521 | -14.43826 |
| C | -22.44382 | 5.51592 | -13.58105 |
| C | -21.97331 | 4.88967 | -12.26344 |
| C | -21.12153 | 5.83089 | -11.40390 |
| C | -20.65990 | 5.20699 | -10.08202 |
| C | -19.81138 | 6.14893 | -9.22019 |
| C | -19.36050 | 5.52648 | -7.89291 |
| C | -18.51036 | 6.48086 | -7.04539 |
| C | 7.48856 | -23.94552 | 2.77621 |
| C | 6.25597 | -23.12067 | 2.39505 |
| C | 5.38497 | -22.74107 | 3.59824 |
| C | 4.14751 | -21.91629 | 3.22733 |
| C | 3.27950 | -21.53551 | 4.43225 |
| C | 2.03884 | -20.71469 | 4.06250 |
| C | 1.17580 | -20.33004 | 5.26975 |
| C | -0.07077 | -19.51720 | 4.90240 |
| C | -0.92516 | -19.12502 | 6.11340 |
| C | -2.18142 | -18.32583 | 5.74944 |
| C | -3.02172 | -17.92072 | 6.96668 |
| C | -4.28329 | -17.12982 | 6.59835 |
| C | -9.79630 | -13.26954 | 16.75294 |
| C | -10.21762 | -13.28438 | 15.28075 |
| C | -9.61098 | -14.44704 | 14.48630 |
| C | -10.03420 | -14.46095 | 13.01192 |
| C | -9.50863 | -15.65426 | 12.19825 |
| C | -7.98767 | -15.67848 | 11.99923 |
| C | -7.50800 | -16.83242 | 11.11010 |
| C | -5.98454 | -16.85608 | 10.92665 |
| C | -5.46238 | -18.00231 | 10.04355 |
| C | -5.78848 | -17.89835 | 8.54242 |
| C | 12.73157 | 6.66539 | -9.22345 |
| C | 13.07905 | 5.40996 | -10.02869 |
| C | 13.15358 | 4.14103 | -9.17177 |
| C | 13.50449 | 2.87945 | -9.96846 |
| C | 13.58083 | 1.61113 | -9.11085 |
| C | 13.93903 | 0.35132 | -9.90746 |
| C | 14.02284 | -0.91648 | -9.04981 |
| C | 14.39208 | -2.17303 | -9.84657 |
| C | 14.48945 | -3.43960 | -8.98855 |
| C | 14.89986 | -4.68056 | -9.79478 |
| H | 4.55141 | 13.59250 | 2.16110 |
| H | 3.71065 | 11.40594 | 1.39858 |
| H | 3.77198 | 9.29137 | 7.84417 |

| | | | |
|---|---|---|---|
| H | 4.63140 | 11.50154 | 8.50858 |
| H | 2.16542 | 5.34396 | 6.49324 |
| H | 3.03965 | 7.49799 | 7.25978 |
| H | 3.03458 | 9.59314 | 0.81239 |
| H | 2.16833 | 7.39712 | 0.16622 |
| H | 5.28959 | 16.61498 | 5.86237 |
| H | 5.71071 | 15.97208 | 7.46015 |
| H | 7.51712 | 15.90481 | 5.00362 |
| H | 9.43726 | -4.70967 | -10.36954 |
| H | 7.40953 | -3.73388 | -9.36823 |
| H | 10.09798 | -4.79270 | -3.23059 |
| H | 12.07059 | -5.78569 | -4.32147 |
| H | 6.43365 | -3.00813 | -1.39239 |
| H | 8.46184 | -3.96247 | -2.37426 |
| H | 5.73994 | -2.98876 | -8.50623 |
| H | 3.75803 | -2.06219 | -7.40825 |
| H | 13.94265 | -7.71575 | -8.31742 |
| H | 13.45433 | -7.11350 | -9.91726 |
| H | 15.32535 | -5.72881 | -7.94391 |
| H | -14.13825 | 5.83337 | -1.05449 |
| H | -11.94368 | 4.87300 | -0.48390 |
| H | -10.74053 | 4.04293 | -7.10842 |
| H | -12.95981 | 5.00331 | -7.57998 |
| H | -6.72359 | 2.36548 | -6.10307 |
| H | -8.91792 | 3.28387 | -6.68063 |
| H | -10.13438 | 4.06820 | -0.05770 |
| H | -7.92129 | 3.12708 | 0.39547 |
| H | -16.67852 | 7.65619 | -5.86945 |
| H | -17.26117 | 7.33339 | -4.23109 |
| H | -17.40971 | 4.96234 | -6.01314 |
| H | -4.25123 | -11.45893 | 9.23655 |
| H | -3.44749 | -9.27225 | 8.43600 |
| H | -5.00644 | -11.17011 | 2.11245 |
| H | -5.78619 | -13.32875 | 3.00634 |
| H | -3.57028 | -7.20150 | 0.63897 |
| H | -4.36159 | -9.38188 | 1.41977 |
| H | -2.79298 | -7.49073 | 7.73806 |
| H | -2.02856 | -5.34680 | 6.83787 |
| H | -6.86699 | -16.09939 | 6.65568 |
| H | -6.81968 | -15.44347 | 8.30522 |
| H | -4.45568 | -16.19315 | 8.51868 |
| H | -1.81020 | -2.60530 | -0.29426 |
| H | -0.64720 | -1.20990 | 4.36846 |
| H | 0.32288 | 1.23651 | 4.28816 |
| H | 0.34881 | 2.77514 | -0.47335 |
| H | 2.02023 | -1.04389 | -0.03619 |
| H | 0.00752 | -0.36001 | -4.56631 |
| H | -2.40016 | 0.64185 | -4.26663 |
| H | -3.30379 | 1.17599 | 0.62605 |
| H | 6.94939 | 29.25525 | 1.98805 |
| H | 6.95083 | 27.94284 | 0.80607 |
| H | 5.53547 | 28.20896 | 1.82738 |
| H | 6.90090 | 27.60236 | 3.86468 |
| H | 8.30406 | 27.33528 | 2.84964 |
| H | 5.68870 | 25.75228 | 2.67546 |
| H | 7.09422 | 25.48395 | 1.66211 |
| H | 7.04696 | 25.18452 | 4.71108 |
| H | 8.45295 | 24.91553 | 3.69838 |
| H | 7.24516 | 23.06283 | 2.50978 |
| H | 5.83491 | 23.33468 | 3.51538 |
| H | 7.17967 | 22.76735 | 5.55833 |
| H | 8.59185 | 22.49605 | 4.55489 |
| H | 7.39261 | 20.64275 | 3.36090 |
| H | 5.97288 | 20.91905 | 4.35119 |
| H | 7.29230 | 20.35113 | 6.40868 |
| H | 8.71686 | 20.07874 | 5.42324 |

| | | | |
|---|---|---|---|
| H | 7.53466 | 18.22211 | 4.21624 |
| H | 6.10099 | 18.51148 | 5.17477 |
| H | 8.83739 | 17.70101 | 6.29315 |
| H | 7.40155 | 17.94142 | 7.26418 |
| H | 14.68661 | 10.73555 | 19.67014 |
| H | 14.39730 | 12.46204 | 19.43487 |
| H | 13.04190 | 11.37484 | 19.74791 |
| H | 14.81712 | 11.29410 | 17.23484 |
| H | 13.47100 | 10.21611 | 17.54499 |
| H | 11.90113 | 12.17521 | 17.54578 |
| H | 13.24798 | 13.25420 | 17.23553 |
| H | 13.69408 | 12.07297 | 15.06311 |
| H | 12.34660 | 10.99453 | 15.37271 |
| H | 10.77639 | 12.95507 | 15.37096 |
| H | 12.12375 | 14.03450 | 15.06493 |
| H | 11.22634 | 11.77727 | 13.19792 |
| H | 12.57547 | 12.85494 | 12.89318 |
| H | 11.00595 | 14.81790 | 12.89528 |
| H | 9.65709 | 13.73792 | 13.19264 |
| H | 10.11638 | 12.56591 | 11.01829 |
| H | 11.46734 | 13.64407 | 10.72290 |
| H | 8.54567 | 14.52290 | 11.00638 |
| H | 9.89562 | 15.60723 | 10.72651 |
| H | 10.37613 | 14.44775 | 8.55163 |
| H | 9.02535 | 13.36281 | 8.83241 |
| H | 7.44398 | 15.27885 | 8.80837 |
| H | 8.78140 | 16.40008 | 8.57438 |
| H | 8.05026 | 14.14073 | 6.63779 |
| H | 9.34041 | 15.29475 | 6.40311 |
| H | 30.81832 | -9.51379 | -11.29368 |
| H | 30.28139 | -7.83108 | -11.29018 |
| H | 30.28705 | -8.75863 | -9.78795 |
| H | 28.50394 | -10.28055 | -10.72897 |
| H | 28.49884 | -9.36000 | -12.21994 |
| H | 27.74760 | -7.32252 | -10.96003 |
| H | 27.75326 | -8.24364 | -9.46798 |
| H | 26.00731 | -9.78459 | -10.41306 |
| H | 26.00109 | -8.86248 | -11.90440 |
| H | 25.24678 | -6.82574 | -10.64175 |
| H | 25.25339 | -7.74804 | -9.15043 |
| H | 23.51215 | -9.29209 | -10.09838 |
| H | 23.50415 | -8.36772 | -11.58819 |
| H | 22.74348 | -6.33503 | -10.32272 |
| H | 22.75241 | -7.25965 | -8.83274 |
| H | 21.00645 | -7.88092 | -11.27245 |
| H | 21.01782 | -8.80886 | -9.78493 |
| H | 20.23546 | -5.85498 | -10.00244 |
| H | 20.24840 | -6.78319 | -8.51456 |
| H | 18.52465 | -8.34088 | -9.47300 |
| H | 18.50682 | -7.40700 | -10.95676 |
| H | 17.72563 | -5.39428 | -9.68260 |
| H | 17.74273 | -6.32217 | -8.19487 |
| H | 16.05991 | -7.89752 | -9.11173 |
| H | 16.00703 | -7.01179 | -10.62453 |
| H | -22.59515 | -0.47602 | 5.54836 |
| H | -21.25352 | -1.15816 | 4.62397 |
| H | -21.01693 | 0.31699 | 5.56489 |
| H | -22.63748 | 1.53176 | 4.05547 |
| H | -22.87072 | 0.06837 | 3.12032 |
| H | -20.44252 | 0.08730 | 2.48347 |
| H | -20.20889 | 1.55194 | 3.41920 |
| H | -21.84753 | 2.75652 | 1.94406 |
| H | -22.08307 | 1.29208 | 1.00897 |
| H | -19.65522 | 1.31144 | 0.36597 |
| H | -19.41851 | 2.77553 | 1.30197 |
| H | -21.06138 | 3.98111 | -0.16560 |

| | | | |
|---|---|---|---|
| H | -21.30166 | 2.51751 | -1.10076 |
| H | -18.87726 | 2.53748 | -1.75589 |
| H | -18.63527 | 4.00142 | -0.82027 |
| H | -20.28697 | 5.20728 | -2.27358 |
| H | -20.53398 | 3.74535 | -3.21049 |
| H | -17.87020 | 5.22675 | -2.93539 |
| H | -18.11743 | 3.76933 | -3.88727 |
| H | -19.51699 | 6.45179 | -4.38088 |
| H | -19.83344 | 4.97353 | -5.26288 |
| H | -26.29599 | 4.42437 | -19.69594 |
| H | -25.41194 | 3.04328 | -19.03965 |
| H | -26.86416 | 3.62957 | -18.22439 |
| H | -25.68391 | 5.80023 | -17.69847 |
| H | -24.24210 | 5.21664 | -18.50540 |
| H | -24.05898 | 3.35364 | -16.83231 |
| H | -25.50277 | 3.93643 | -16.02578 |
| H | -24.34775 | 6.11303 | -15.53212 |
| H | -22.90320 | 5.52808 | -16.33571 |
| H | -22.72475 | 3.66570 | -14.65872 |
| H | -24.17053 | 4.24939 | -13.85657 |
| H | -21.56965 | 5.83924 | -14.16088 |
| H | -23.01667 | 6.42676 | -13.36374 |
| H | -21.39940 | 3.97934 | -12.48009 |
| H | -22.84809 | 4.56546 | -11.68496 |
| H | -21.69388 | 6.74333 | -11.19201 |
| H | -20.24344 | 6.15108 | -11.97943 |
| H | -20.08621 | 4.29522 | -10.29306 |
| H | -21.53895 | 4.88581 | -9.50826 |
| H | -20.38360 | 7.06291 | -9.01416 |
| H | -18.92864 | 6.46547 | -9.78993 |
| H | -18.77842 | 4.61893 | -8.09968 |
| H | -20.24768 | 5.20294 | -7.33761 |
| H | -17.66465 | 6.81655 | -7.65333 |
| H | -19.10007 | 7.37629 | -6.80329 |
| H | 8.08837 | -24.19894 | 1.89772 |
| H | 8.13219 | -23.39626 | 3.47051 |
| H | 7.20189 | -24.88253 | 3.26402 |
| H | 5.64931 | -23.68177 | 1.67385 |
| H | 6.57375 | -22.20715 | 1.87787 |
| H | 5.99241 | -22.17904 | 4.31962 |
| H | 5.06865 | -23.65563 | 4.11677 |
| H | 4.46426 | -21.00304 | 2.70705 |
| H | 3.53984 | -22.47976 | 2.50748 |
| H | 2.96588 | -22.44867 | 4.95450 |
| H | 3.88679 | -20.96948 | 5.15041 |
| H | 1.42908 | -21.28269 | 3.34804 |
| H | 2.35144 | -19.80326 | 3.53656 |
| H | 1.78472 | -19.75692 | 5.98090 |
| H | 0.86920 | -21.24126 | 5.79955 |
| H | -0.68411 | -20.09383 | 4.19801 |
| H | 0.23400 | -18.60929 | 4.36586 |
| H | -0.31347 | -18.53951 | 6.81201 |
| H | -1.21965 | -20.03243 | 6.65651 |
| H | -1.89005 | -17.42463 | 5.19460 |
| H | -2.80046 | -18.91688 | 5.06245 |
| H | -3.29226 | -18.82388 | 7.52686 |
| H | -2.40520 | -17.31704 | 7.64540 |
| H | -3.97798 | -16.24277 | 6.03259 |
| H | -4.90707 | -17.72083 | 5.91608 |
| H | -10.24576 | -12.42944 | 17.28973 |
| H | -10.10197 | -14.18960 | 17.26105 |
| H | -8.70963 | -13.18384 | 16.85250 |
| H | -9.92876 | -12.33711 | 14.80921 |
| H | -11.31139 | -13.33442 | 15.21441 |
| H | -9.90552 | -15.39594 | 14.95434 |
| H | -8.51866 | -14.39655 | 14.56244 |

```
H    -9.70803  -13.52818   12.53337
H   -11.13011  -14.45833   12.96292
H    -9.82717  -16.58803   12.68022
H    -9.99104  -15.64400   11.21308
H    -7.48447  -15.74718   12.97053
H    -7.66587  -14.72744   11.55581
H    -8.00615  -16.75870   10.13567
H    -7.83537  -17.78674   11.54388
H    -5.51947  -16.94624   11.91601
H    -5.65134  -15.89242   10.52579
H    -5.85790  -18.95263   10.42410
H    -4.37312  -18.06303   10.15357
H    -5.47326  -18.82922    8.06171
H    -6.87518  -17.86021    8.40361
H    12.68494    7.55127   -9.86283
H    11.76075    6.56158   -8.72859
H    13.47868    6.85562   -8.44651
H    12.33446    5.26636  -10.82115
H    14.03901    5.55752  -10.53824
H    13.89709    4.28603   -8.37716
H    12.19206    3.99286   -8.66288
H    14.46553    3.02958  -10.47725
H    12.76086    2.73483  -10.76288
H    12.61883    1.45808   -8.60468
H    14.32195    1.75766   -8.31449
H    13.19683    0.20218  -10.70229
H    14.89947    0.50697  -10.41572
H    13.06095   -1.07665   -8.54565
H    14.76139   -0.76456   -8.25204
H    15.35081   -2.00820  -10.35553
H    13.65079   -2.33108  -10.64009
H    15.22055   -3.27825   -8.18591
H    13.52863   -3.60793   -8.49060
H    14.18286   -4.84608  -10.60613
H    15.85669   -4.45670  -10.27884
```

**Energies used to compute the reorganization energies; corresponding to the optimized ground-state neutral structures (RELAX_GS), to the optimized ground-state cationic structures (RELAX_CATION), to the optimized ground-state anionic structures (RELAX_ANION), to the cationic form with the neutral ground-state structure (CATIONwithGS), to the anionic form with the neutral ground-state structure (ANIONwithGS), to the neutral form with cationic structure (GSwithCATION), and to the neutral form with anionic structure (GSwithANION).**

| System | Etot (in A.U.) | Etot (in A.U.) | Etot (in A.U.) | Etot (in A.U.) | In eV | In eV | In eV |
|---|---|---|---|---|---|---|---|
| NIP-dipb | RELAX_GS_1 | CATIONwithGS_2 | RELAX_CATION_3 | GSwithCATION_4 | l1(4-1) | l2(2-3) | l_h=l1+l2 |
| | -1549,607652 | -1549,322617 | -1549,324788 | -1549,605039 | 0,071107 | 0,059074 | 0,130180 |
| | RELAX_GS_1 | ANIONwithGS_2 | RELAX_ANION_3 | GSwithANION_4 | l1(4-1) | l2(2-3) | l_e=l1+l2 |
| | -1549,607652 | -1549,681156 | -1549,687458 | -1549,601355 | 0,171346 | 0,171466 | 0,342812 |
| NIP-dt | RELAX_GS_1 | CATIONwithGS_2 | RELAX_CATION_3 | GSwithCATION_4 | l1(4-1) | l2(2-3) | l_h=l1+l2 |
| | -2026,345895 | -2026,051877 | -2026,053907 | -2026,343768 | 0,057865 | 0,055250 | 0,113116 |
| | RELAX_GS_1 | ANIONwithGS_2 | RELAX_ANION_3 | GSwithANION_4 | l1(4-1) | l2(2-3) | l_e=l1+l2 |
| | -2026,345895 | -2026,417948 | -2026,424164 | -2026,339796 | 0,165966 | 0,169161 | 0,335127 |

| | | | | | | | |
|---|---|---|---|---|---|---|---|
| **PIPB-dipb** | **RELAX_GS_1** | **CATIONwithGS_2** | **RELAX_CATION_3** | **GSwithCATION_4** | **I1(4-1)** | **I2(2-3)** | **I_h=I1+I2** |
| | -1933,213105 | -1932,953330 | -1932,955225 | -1933,211078 | 0,055152 | 0,051569 | **0,106721** |
| | **RELAX_GS_1** | **ANIONwithGS_2** | **RELAX_ANION_3** | **GSwithANION_4** | **I1(4-1)** | **I2(2-3)** | **I_e=I1+I2** |
| | -1933,213105 | -1933,298837 | -1933,303788 | -1933,208224 | 0,132809 | 0,134716 | **0,267525** |
| **PIPB-dt** | **RELAX_GS_1** | **CATIONwithGS_2** | **RELAX_CATION_3** | **GSwithCATION_4** | **I1(4-1)** | **I2(2-3)** | **I_h=I1+I2** |
| | -2409,945650 | -2409,685648 | -2409,688042 | -2409,943193 | 0,066873 | 0,065142 | **0,132014** |
| | **RELAX_GS_1** | **ANIONwithGS_2** | **RELAX_ANION_3** | **GSwithANION_4** | **I1(4-1)** | **I2(2-3)** | **I_e=I1+I2** |
| | -2409,945650 | -2410,030452 | -2410,035297 | -2409,940840 | 0,130899 | 0,131824 | **0,262723** |
| **4NIPBSP-dipb** | **RELAX_GS_1** | **CATIONwithGS_2** | **RELAX_CATION_3** | **GSwithCATION_4** | **I1(4-1)** | **I2(2-3)** | **I_h=I1+I2** |
| | -6231,727456 | -6231,442900 | -6231,444456 | -6231,725085 | 0,064527 | 0,042345 | **0,106872** |
| | **RELAX_GS_1** | **ANIONwithGS_2** | **RELAX_ANION_3** | **GSwithANION_4** | **I1(4-1)** | **I2(2-3)** | **I_e=I1+I2** |
| | -6231,727456 | -6231,802573 | -6231,807456 | -6231,722327 | 0,139562 | 0,132886 | **0,272448** |
| **4NIPBSP-dt** | **RELAX_GS_1** | **CATIONwithGS_2** | **RELAX_CATION_3** | **GSwithCATION_4** | **I1(4-1)** | **I2(2-3)** | **I_h=I1+I2** |
| | -8138,672759 | -8138,380775 | -8138,382659 | -8138,670835 | 0,052345 | 0,051274 | **0,103619** |
| | **RELAX_GS_1** | **ANIONwithGS_2** | **RELAX_ANION_3** | **GSwithANION_4** | **I1(4-1)** | **I2(2-3)** | **I_e=I1+I2** |
| | -8138,672759 | -8138,748759 | -8138,753989 | -8138,667536 | 0,142129 | 0,142313 | **0,284442** |
| **4PIPBSP-dipb** | **RELAX_GS_1** | **CATIONwithGS_2** | **RELAX_CATION_3** | **GSwithCATION_4** | **I1(4-1)** | **I2(2-3)** | **I_h=I1+I2** |
| | -7766,126102 | -7765,837837 | -7765,839532 | -7766,124398 | 0,046366 | 0,046134 | **0,092501** |
| | **RELAX_GS_1** | **ANIONwithGS_2** | **RELAX_ANION_3** | **GSwithANION_4** | **I1(4-1)** | **I2(2-3)** | **I_e=I1+I2** |
| | -7766,126102 | -7766,213285 | -7766,217637 | -7766,121715 | 0,119366 | 0,118435 | **0,237802** |
| **4PIPBSP-dt** | **RELAX_GS_1** | **CATIONwithGS_2** | **RELAX_CATION_3** | **GSwithCATION_4** | **I1(4-1)** | **I2(2-3)** | **I_h=I1+I2** |
| | -9673,094783 | -9672,803644 | -9672,805483 | -9673,092940 | 0,050153 | 0,050024 | **0,100176** |
| | **RELAX_GS_1** | **ANIONwithGS_2** | **RELAX_ANION_3** | **GSwithANION_4** | **I1(4-1)** | **I2(2-3)** | **I_e=I1+I2** |
| | -9673,094783 | -9673,171811 | -9673,176235 | -9673,090318 | 0,121479 | 0,120379 | **0,241857** |

## 5. References


1. Alonso-Navarro, M. J.; Harbuzaru, A.; de Echegaray, P.; Arrechea-Marcos, I.; Harillo-Baños, A.; de la Peña, A.; Ramos, M. M.; López Navarrete, J. T.; Campoy-Quiles, M.; Ponce Ortiz, R.; Segura, J. L., Effective interplay of donor and acceptor groups for tuning optoelectronic properties in oligothiophene–naphthalimide assemblies. *Journal of Materials Chemistry C* **2020,** *8* (43), 15277-15289.
2. de Echegaray, P.; Mancheño, M. J.; Arrechea-Marcos, I.; Juárez, R.; López-Espejo, G.; López Navarrete, J. T.; Ramos, M. M.; Seoane, C.; Ortiz, R. P.; Segura, J. L., Synthesis of Perylene Imide Diones as Platforms for the Development of Pyrazine Based Organic Semiconductors. *The Journal of Organic Chemistry* **2016,** *81* (22), 11256-11267.
3. Adel, R.; Gala, E.; Alonso-Navarro, M. J.; Gutierrez-Fernandez, E.; Martín, J.; Stella, M.; Martinez-Ferrero, E.; de la Peña, A.; Harbuzaru, A.; Ramos, M. M.; Ortiz, R. P.; Segura, J. L.; Campoy-Quiles, M., Comparing the microstructure and photovoltaic performance of 3 perylene imide acceptors with similar energy levels but different packing tendencies. *Journal of Materials Chemistry C* **2022,** *10* (5), 1698-1710.

4. Ahmad, I.; Mahmood, J.; Baek, J.-B., Scalable Synthesis of Tetrapodal Octaamine. *European Journal of Organic Chemistry* **2019,** *2019* (13), 2335-2338.
5. Pyka, I.; Ryvlin, D.; Waldvogel, S. R., Application of Rigidity-Controlled Supramolecular Affinity Materials for the Gravimetric Detection of Hazardous and Illicit Compounds. *ChemPlusChem* **2016,** *81* (9), 926-929.
6. Suárez-Blas, F.; Li, J.; Alonso-Navarro, M. J.; Harbuzaru, A.; Ponce Ortiz, R.; Ramos, M. M.; Shalom, M.; Barrio, J.; Segura, J. L., Naphthalimide-Based 3D Organic Semiconductors: Synthesis and Application as Photo-Electrocatalysts for Organic Dyes Degradation and Water Splitting. *Advanced Sustainable Systems* **2023,** *7* (2), 2200339.
7. Becke, A. D., Density-functional thermochemistry. III. The role of exact exchange. *The Journal of Chemical Physics* **1993,** *98* (7), 5648-5652.
8. Curtiss, L. A.; McGrath, M. P.; Blaudeau, J. P.; Davis, N. E.; Binning, R. C., Jr.; Radom, L., Extension of Gaussian-2 theory to molecules containing third-row atoms Ga–Kr. *The Journal of Chemical Physics* **1995,** *103* (14), 6104-6113.
9. G. W. S. M. J. T. Frisch, H. B. S., G. E.; Robb, M. A.; Cheeseman, J. R.; Scalmani, G.; Barone, V.; Petersson, G. A.; Nakatsuji, H.; Li, X.; Caricato, M.; Marenich, A. V.; Bloino, J.; Janesko, B. G.; Gomperts, R.; Mennucci, B.; Hratchian, H. P.; Ortiz, J. V.; Izmaylov, A. F.; Sonnenberg, J. L.; Williams-Young, D.; Ding, F.; Lipparini, F.; Egidi, F.; Goings, J.; Peng, B.; Petrone, A.; Henderson, T.; Ranasinghe, D.; Zakrzewski, V. G.; Gao, J.; Rega, N.; Zheng, G.; Liang, W.; Hada, M.; Ehara, M.; Toyota, K.; Fukuda, R.; Hasegawa, J.; Ishida, M.; Nakajima, T.; Honda, Y.; Kitao, O.; Nakai, H.; Vreven, T.; Throssell, K.; Montgomery, J. A., Jr.; Peralta, J. E.; Ogliaro, F.; Bearpark, M. J.; Heyd, J. J.; Brothers, E. N.; Kudin, K. N.; Staroverov, V. N.; Keith, T. A.; Kobayashi, R.; Normand, J.; Raghavachari, K.; Rendell, A. P.; Burant, J. C.; Iyengar, S. S.; Tomasi, J.; Cossi, M.; Millam, J. M.; Klene, M.; Adamo, C.; Cammi, R.; Ochterski, J. W.; Martin, R. L.; Morokuma, K.; Farkas, O.; Foresman, J. B.; Fox, D. J., Gaussian, Inc., Wallingford CT. **2016**.
10. Runge, E.; Gross, E. K. U., Density-Functional Theory for Time-Dependent Systems. *Physical Review Letters* **1984,** *52* (12), 997-1000.
11. Petersilka, M.; Gossmann, U. J.; Gross, E. K. U., Excitation Energies from Time-Dependent Density-Functional Theory. *Physical Review Letters* **1996,** *76* (8), 1212-1215.
12. Casida, M. E.; Jamorski, C.; Casida, K. C.; Salahub, D. R., Molecular excitation energies to high-lying bound states from time-dependent density-functional response theory: Characterization and correction of the time-dependent local density approximation ionization threshold. *The Journal of Chemical Physics* **1998,** *108* (11), 4439-4449.
13. Malagoli, M.; Brédas, J., Density functional theory study of the geometric structure and energetics of triphenylamine-based hole-transporting molecules. *Chemical Physics Letters - CHEM PHYS LETT* **2000,** *327*, 13-17.
